# Glycosylated cyclophellitol-derived activity-based probes and inhibitors for cellulases†

**DOI:** 10.1039/d0cb00045k

**Published:** 2020-07-28

**Authors:** Casper de Boer, Nicholas G. S. McGregor, Evert Peterse, Sybrin P. Schröder, Bogdan I. Florea, Jianbing Jiang, Jos Reijngoud, Arthur F. J. Ram, Gilles P. van Wezel, Gijsbert A. van der Marel, Jeroen D. C. Codée, Herman S. Overkleeft, Gideon J. Davies

**Affiliations:** Leiden Institute of Chemistry, Leiden University Einsteinweg 55 2300 RA Leiden The Netherlands h.s.overkleeft@chem.leidenuniv.nl; York Structural Biology Laboratory, Department of Chemistry, The University of York Heslington York YO10 5DD UK Gideon.davies@york.ac.uk; Institute of Biology Leiden, Leiden University Sylviusweg 72 2333 BE Leiden The Netherlands

## Abstract

Cellulases and related β-1,4-glucanases are essential components of lignocellulose-degrading enzyme mixtures. The detection of β-1,4-glucanase activity typically relies on monitoring the breakdown of purified lignocellulose-derived substrates or synthetic chromogenic substrates, limiting the activities which can be detected and complicating the tracing of activity back to specific components within complex enzyme mixtures. As a tool for the rapid detection and identification of β-1,4-glucanases, a series of glycosylated cyclophellitol inhibitors mimicking β-1,4-glucan oligosaccharides have been synthesised. These compounds are highly efficient inhibitors of HiCel7B, a well-known GH7 *endo*-β-1,4-glucanase. An elaborated activity-based probe facilitated the direct detection and identification of β-1,4-glucanases within a complex fungal secretome without any detectable cross-reactivity with β-d-glucosidases. These probes and inhibitors add valuable new capacity to the growing toolbox of cyclophellitol-derived probes for the activity-based profiling of biomass-degrading enzymes.

## Abstract 2

New cyclophellitol-derived activity-based probes enable the sensitive detection and identification of cellulases.

## Graphical abstract

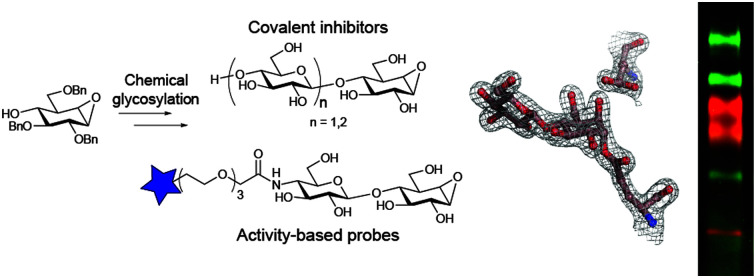

## Introduction

Activity-based protein profiling (ABPP) is a powerful technique used to detect and identify active enzymes using modified covalent inhibitors. Following early successes in the detection and identification of proteases,^1^ this technique has been extended to glycoside hydrolases using a variety of strategies,^2,3^ including labelled cyclophellitol derivatives.^4^

Though often considered synonymous with cellulases, β-1,4-glucanases are enzymes which recognize β-1,4-linked glucan chains which are characteristic of both the cellulosic and hemicellulosic (*i.e.* mixed-linkage glucans, xyloglucans, and glucomannans) fractions of plant biomass.^5^ The catalytic actions of a variety of retaining β-1,4-glucanases contribute to the breakdown of lignocellulosic polysaccharides.^6,7^ The efficient and specific profiling of β-1,4-glucanases is thus a valuable tool in the study of biomass-degrading organisms.

Efforts have been made to profile β-1,4-glycanases using different “warhead” chemistries. Activity-based probes (ABPs) based on a 2,4-dinitrophenyl 2-deoxy-2-fluoro-β-xylobioside/cellobioside modified with an affinity tag at the 4’ position proved effective probes for retaining β-d-glucanases and β-xylanases with particularly good specificity.^2,8,9^ However, the slow hydrolysis of the enzyme-probe complex and the weak initial binding of these probes necessitated the use of high probe concentration (∼0.5–1 mM) and time-limited labelling for ABPP. Later experiments using difluoromethylphenyl glycosides and *N*-haloacetyl glycosylamines demonstrated a unique capacity to label inverting glycoside hydrolases, but suffered from significant non-specific labelling.^3^

Cyclophellitol is an inhibitor of β-d-glucosidases originally isolated from the *Phellinus* mushroom.^10^ This cyclitol is an isostere of a glucoside where the acetal group is replaced by an epoxide. Taking advantage of the catalytic machinery of a retaining glycoside hydrolase, this epoxide undergoes an acid-catalysed ring opening addition to form a non-hydrolysable ester in place of the normal glycosyl-enzyme intermediate, irreversibly inactivating the enzyme.^11^

ABPs built around synthetic cyclitols, having configurations which target α- and β-d-glucosidases,^12–14^ β-d-glucuronidases,^15^ β-d-xylosidases,^16^ α- and β-d-galactosidases,^17,18^ and α-l-arabinofuranosidases,^19^ among others, have consistently been shown to covalently modify the catalytic nucleophiles of cognate retaining glycosidases. These cyclophellitol-derived ABPs generally bind with good specificity, high affinity, and complete irreversibility.

Recent work has shown that cyclophellitol derivatives can be glycosylated, enabling the development of inhibitors and probes which react specifically with *endo*-glycanases.^16^ This was first demonstrated with the development of an inhibitor and a collection of ABPs for β-1,4-xylanases. Being built around a xylobiose core with an alkylated aziridine warhead, these probes proved to be potent covalent inhibitors of GH10 β-1,4-xylanases, but showed cross-reactivity with β-d-xylosidases when applied to the direct detection of β-xylanases within fungal secretomes. This cross-reactivity was traced back to the internal hydrolysis of the probe by the action of β-d-xylosidases.

Building on this understanding, here we report the development of cyclophellitol-derived ABPs designed to target β-1,4-glucanases, some of the most abundant glycoside hydrolases in nature. To detect and profile these enzymes, a collection of 4-O substituted (carbohydrate numbering) cyclophellitols have been synthesised and tested for their ability to covalently modify HiCel7B, a well-known *endo*-β-1,4-glucanase. Through biochemical, structural, and mass spectrometric analyses, we have identified a potent substrate-mimicking probe architecture which shows resistance to hydrolysis by *exo*-glucosidase and *endo*-glucanase activities within a fungal secretome.

## Experimental

### Synthesis of β-d-glucanase inhibitors and probe

Chemical synthesis details and compound analysis is reported in the ESI.†

### Testing β-d-glucanase inhibitors and probes

HiCel7B was a kind gift from Martin Schülein (sadly now deceased) at Novozymes A/S (Lyngby, Denmark). The pH-activity profile of HiCel7B acting on 4-methylumbelliferyl β-d-cellobioside (4MU-GG) was measured by combining 5 μL of 1 μg mL^−1^ HiCel7B in 200 mM 2 : 7 : 7 succinate–phosphate–glycine (SPG) buffer prepared at various pH values with 45 μL of 0.1 mM substrate in quadruplicate. The reactions were incubated at 25 °C for 30 minutes prior to the addition of 5 μL of 1 M Na_2_CO_3_, transfer of 50 μL to a black 384-well plate and fluorescence measurement (*λ*_ex_ = 360 nm, *λ*_em_ = 450 nm). Rates were determined using a 4-methylumbelliferone calibration series prepared in 0.1 M Na_2_CO_3_.

Intact MS of HiCel7B bound to different inhibitors was performed according to McGregor *et al.*^19^ Briefly, enzyme was diluted to 0.1 mg mL^−1^ (∼2.2 μM) in 20 mM sodium phosphate buffer pH 7. Compounds **1**, **5**, or **13** were added to a final concentration of 5 μM and incubated at 25 °C. Samples taken at 1 hour were diluted with 4 volumes of 1% formic acid, 10% acetonitrile and analysed. Additional experiments with 5 were performed using different concentrations of inhibitor and enzyme as indicated in the text.

Inhibition kinetics for compounds **1**, **5**, and **13** acting on HiCel7B were measured at 25 °C using 4MU-GG following a method described previously.^19^ Briefly, enzyme was diluted in 50 mM sodium phosphate buffer pH 7. Substrate was dissolved in DMSO to give a 10 mM stock which was diluted with ultrapure water. Inhibitors were dissolved in and diluted with ultrapure water with the exception of **13** which was dissolved in DMSO to give a 5 mM stock which was diluted with ultrapure water. The enzyme and substrate concentrations used in the continuous inhibition assays were 10 ng mL^−1^ (∼220 pM) and 50 μM, respectively. The *K*_M_ value for the interaction of HiCel7B with 4MU-GG under the assay conditions (corrected for inner filter effect) was measured to be 76 μM (Fig. S1, ESI†) and this was used as a correction factor to determine the *K*_I_ values in Table 1 from the apparent *K*_I_ determined from fitting of *k*_app_*vs.* [inhibitor] curves.

**Table 1:** Kinetic parameters for covalent inhibition of HiCel7B by putative β-1,4-glucanase inhibitors

Compound	HiCel7B		
	*K*_I_ (μM)	*k*_inact_ (min^−1^)	*k*_inact_/*K*_I_ (s^−1^ M^−1^)
**1**	31 ± 4	0.84 ± 0.08	450
**5**	1.9 ± 0.3	0.35 ± 0.03	3100
**13**	3.9 ± 0.3	0.50 ± 0.03	2100

### Enzyme crystallisation, diffraction, and structure solution

Deglycosylated HiCel7B was desalted into 20 mM pH 8 Tris–HCl buffer and concentrated to 12 mg mL^−1^ using a 30 kDa MWCO centricon. Building on previous reports,^20,21^ crystallisation conditions were re-screened using the PACT Suite and AmSO4 Suite (Qiagen) crystallisation screens. High quality tetragonal bipyramidal crystals grew consistently at 20 °C from a 2 : 1 mixture of protein solution:well solution, where well solution was 0.15 M sodium citrate, 0.8 M ammonium sulfate, 1 M lithium sulfate (Fig. S2, ESI†). Crystal soaks were performed in a solution composed of 0.1 mM ligand in mother liquor for 5 hours at 20 °C prior to transfer into mother liquor supplemented with 20% glycerol and cryo-cooling in LN_2_.

Crystals were diffracted at Diamond Light Source (Harwell, UK) on beamline I03 at a wavelength of 0.9762 Å and automatically processed using the Xia2^22^ pipeline with 3dii. Computation was carried out using programs from the CCP4 suite^23^ unless otherwise stated. All crystal structure figures were generated using Pymol (Schrodinger). Data collection and processing statistics for all structures are given in Table S1 (ESI†).

Data for HiCel7B bound to compound **1** were collected to 1.88 Å. Data were also collected out to 1.2 Å in a higher space group (*P*4_2_2_1_2) following a soak with **13**, though the structure was found to be unliganded. The structure of **1**-bound HiCel7B was solved in the *P*4_1_22 space group by molecular replacement using Phaser^24^ with the known structure (PDBID: 2A39) as the search model. Ligand **1** was built using the existing restrains for β-d-glucose (BGC) and cyclophellitol (YLL) with Coot,^25^ and structures were refined by alternating rounds of manual model building and density refinement using Coot and REFMAC5^26^ respectively.

### In-gel fluorescence

The pH-labelling profile of HiCel7B reacting with the probe was measured by combining 10 μL of 1 μg mL^−1^ HiCel7B in 200 mM SPG buffer prepared at various pH values with 10 μL of 10 μM **14** in quadruplicate. The reactions were incubated at 25 °C for 10 minutes prior to the addition of 8 μL of 4× SDS-PAGE loading dye and heating to 95 °C for 2 minutes. 10 μL of each reaction was separated on a 10% SDS-PAGE gel prior imaging using the Cy5 laser/filter settings on a Typhoon 5 scanner (GE Healthcare). Bands were integrated using ImageQuant (GE Healthcare).

Secretome staining was performed using two aliquots of 20 μL xylan-grown *Aspergillus niger* secretome (day 4 samples prepared as described previously^16^). To each was added 5 μL of 0.5 M pH 5 McIlvane buffer. To one was then added 5 μL of 60 μM **14** and to the other was added 5 μL of 60 μM **19** (10 μM final ABP concentration). These were incubated for 30 minutes at 37 °C. The reactions were then split in two and one half (15 μL) was diluted with 5 μL of water. The other half (15 μL) of the reaction with **19** was then supplemented with 5 μL of 40 μM **14** and the other half of the reaction with **14** was supplemented with 5 μL of 40 μM **19** (10 μM final ABP concentration). The reactions were incubated for a further 30 minutes at 37 °C before being diluted with 8 μL of 4× SDS-PAGE loading dye, separated on a 4–20% SDS-PAGE gel (Bio-Rad) and imaged for fluorescence using the Cy2 and Cy5 laser/filter settings on a Typhoon 5 scanner (GE Healthcare).

### Biotin–avidin enrichment proteomic analysis

5 aliquots of 1 mL of xylan-grown *Aspergillus niger* secretome were thawed from −80 °C, centrifuged at 12 000 × *g* for 15 minutes to remove particulate and combined. 0.5 mL of this was then subsampled into 9 separate Lo-Bind 2.0 mL tubes. To three tubes was added 55 μL of 1 mM compound **1** in ultrapure water, and to the rest 55 μL of ultrapure water. All samples were incubated for 1 hour at 37 °C prior to the addition of 60 μL of compound **15** in 10% DMSO to the samples treated with **1** and three of the samples not treated with **1**. 10% DMSO was added to the remaining three samples. All of the samples were incubated for a further 2 hours at 37 °C. Proteins were denatured by heating to 95 °C for 5 minutes following the addition of 70 μL of 10× denaturing buffer (500 mM Na-HEPES, pH 7.5, 50 mM DTT, 5% SDS). Once cooled to RT, thiols were alkylated by the addition of 70 μL of 0.25 M iodoacetamide and incubation in the dark for 30 minutes. Samples were transferred to 5 mL Eppendorf tubes and proteins were precipitated by the addition of 3.2 mL of chilled acetone followed by incubation at −20 °C for 1 hour. Proteins were collected by centrifugation (14 000 × *g* for 1 minute) and the supernatant was discarded. The pellet was washed with 3 mL of cold acetone and air-dried. The pellet was then resuspended in 50 μL of 8 M urea, 10 mM HEPES, pH 7.2 and diluted with 150 μL of 0.05% SDS in phosphate-buffered saline (PBS). This was shaken overnight at 20 °C to dissolve. The samples were then diluted with a further 200 μL of 0.05% SDS in PBS and centrifuged to collect any insoluble residue. The supernatant was transferred to a 2 mL Eppendorf tube and mixed with 25 μL of Pierce Avidin Agarose beads (Thermo Fisher Scientific) which had been washed twice with PBS. Following 3 hours of mixing by inversion, beads were collected by centrifugation for 2 minutes at 2500 × *g*. The supernatant was removed and the beads were washed with 500 μL of 0.5% SDS in PBS once, 500 μL of 2% SDS at 65 °C for 10 minutes once, 500 μL of 0.5% SDS in PBS again, then 500 μL of 2 M urea followed by two washes with 500 μL of ultrapure water. The beads were finally resuspended in 20 μL of on-bead digestion buffer and trypsinisation, StageTip desalting, LC-MS/MS data acquisition, and data processing were performed as described previously.^27^ Peptides were identified by searching against a database of *A. niger* NRRL3 proteins^28^ supplemented with streptavidin, avidin, yeast enolase and trypsin. The combined search results were filtered for a minimum of two unique peptides with a false-discovery rate of 4%. Label-free quantification was performed using Progenesis QI (Waters). Following chromatographic alignment, peaks were integrated and assigned. Protein abundance was estimated using the integrated intensity of non-conflicting peptides. Results of this analysis for all identified proteins can be found in Table S2 (ESI†).

## Results and discussion

### Synthesis of potential cellulase inhibitors and probes

**Scheme 1:**
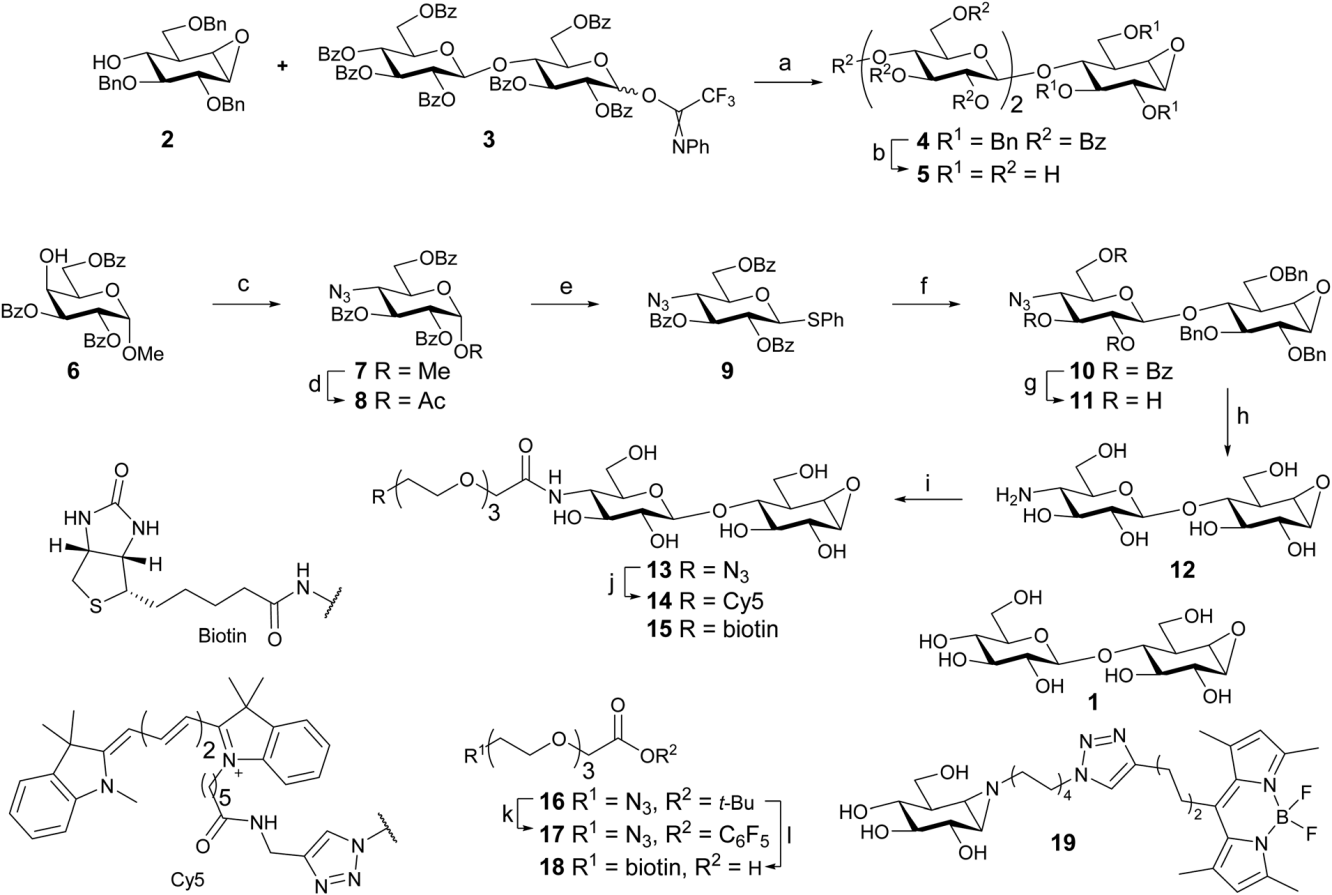
*Reagent and conditions*: (a) TSMOTf, DCM, −15 °C to 0 °C, 45%. (b) (i) NaOMe, MeOH. (ii) H_2_, Pd(OH)_2_/C, H_2_O, MeOH, dioxane, 40%. (c) (i) Tf_2_O, pyr, DCM, −55 °C to rt. (ii) NaN_3_, DMF, 80 °C, 90%. (d) Ac_2_O, AcOH, H_2_SO_4_. (e) HSPh, BF_3_·Et_2_O, DCM, 46% over 2 steps. (f) **2**, Ph_2_SO, Tf_2_O, TTBP, DCM, −70 °C to rt, 64%. (g) NaOMe, MeOH, DCM, 60%. (h) Na (s), *t*-BuOH, NH_3_, −60 °C, 53%. (i) **17**, DIPEA, DMF, 27%; or **18**, PFPOH, DIC, Et_3_N, DMF, 44%. (j) Cy5 alkyne, THPTA, CuI, DIPEA, DMSO, 46%. (k) (i) TFA, DCM; (ii) PFPOH, DIC, DMAP, DCM, 67%. (l) (i) PPh_3_, H_2_O, THF, 88%; (ii) BiotinNHS, DIPEA, DMF, 83%; (iii) TFA, DCM, quant.

Cellobiose configured cyclophellitol (**1**) was synthesized *via* previously reported procedures.^16^ Glycosylation of cyclophellitol acceptor **2** with cellobiose-derived *N*-phenyl trifluoroacetimidate **3** afforded pseudo-trisaccharide **4** (Scheme 1). The deprotection sequence: benzoyl removal by NaOMe in MeOH, followed by short hydrogenation over a high loading of Pearlman's catalyst,^29^ yielded inhibitor **5**.

To gain access to cellobiose configured ABPs 4-deoxy-4-azido-thioglucoside donor **9** was synthesized. The methods are similar to a published synthesis of 4-deoxy-4-fluoro-thioglucoside donors.^30^ The axial 4-OH of partially protected methyl α-d-galactopyranoside **6** was activated as a triflate and substituted by sodium azide leading to **7**. Acid-catalyzed displacement of the anomeric methoxy group afforded anomeric acetate **8**. Introduction of the anomeric thiophenol yielded donor **9**.

The glycosylation reaction was improved, compared to that employed in the inhibitor synthesis. Application of a pre-activation protocol (Tf_2_O/Ph_2_SO) circumvents the use of relatively high temperatures and long reaction times required to activate this type of donor using NIS/TfOH. It also allows the activation of the donor to take place without the presence of the acid-labile epoxide. Disaccharide **10** was obtained in 64% yield without the use of a large excess of donor. Unreacted acceptor (**2**) was also recovered indicating the stability of the epoxide functionality under these conditions. Increasing the amount of donor (**9**) led to diminished yield and complex mixtures. This was presumably due to the reaction of the epoxide in the product with the excess activated donor.^31^

Following the synthesis of disaccharide **10**, the benzoyl esters were removed with NaOMe affording **11**. Staudinger reduction of the azide followed by benzyl removal under Birch conditions afforded fully deprotected **12**.

Azide-terminated triethylene glycol *t*-butyl ester **16**^32^ was deprotected using trifluoroactic acid and DIC/DMAP mediated esterification with pentafluorophenol afforded activated ester **17**. The amine in **12** was selectively acylated with **17**, yielding probe **13** following semi-preparative HPLC purification. Cy5-labeled probe **14** was obtained after copper catalyzed click reaction of **13** with Cy5 alkyne. Biotin-labeled probe **15** was synthesized in one step from **12** by amide bond formation with biotin-terminated spacer **18**, obtained from **16** in 3 steps.

BODIPY green-labeled β-glucosidase probe **19** was obtained by methods developed for the previously reported BODIPY red variant using BODIPY green alkyne.^33,34^

### Testing potential cellulase inhibitors and probes with HiCel7B

*Humicola insolens* Cel7B (HiCel7B) was chosen as a model β-d-glucanase since it is well-characterized, has good hydrolytic performance with chromogenic substrates, can be readily crystallised, and has been studied in our lab previously.^20,21,35^

**Fig. 1:**
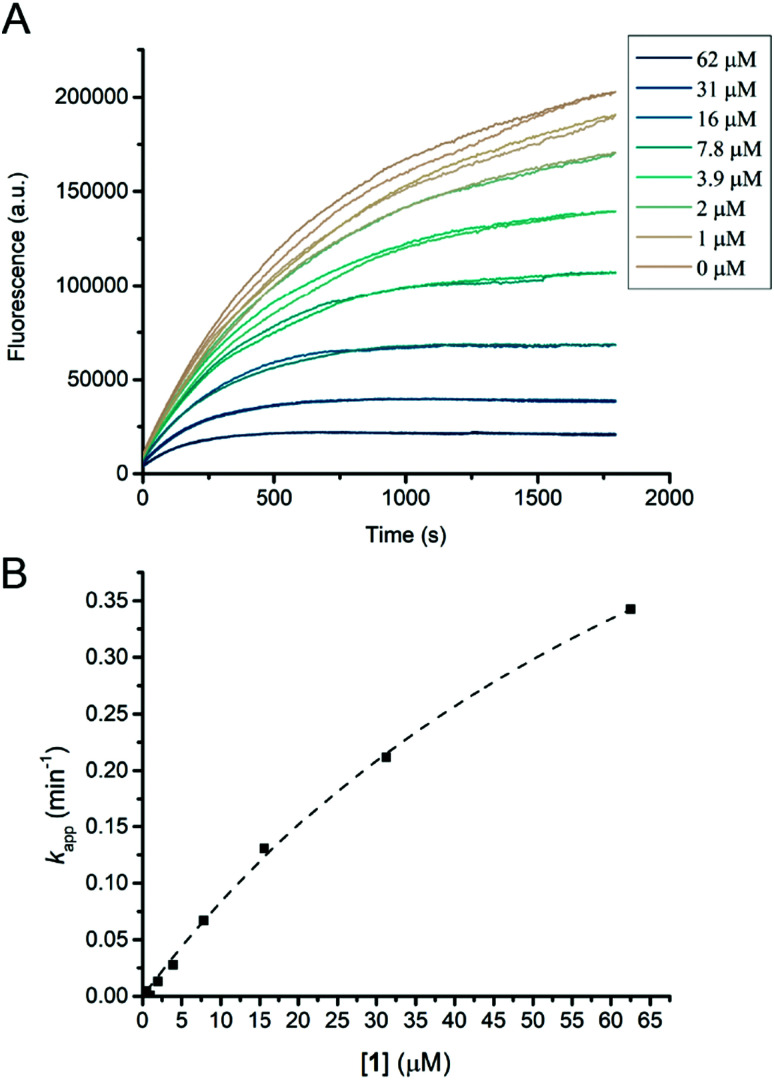
HiCel7B inhibition kinetics with inhibitor **1**. (A) Plot of fluorescence *vs.* time for HiCel7B in the presence of different concentrations of inhibitor **1**. Each line represents a single measurement. (B) Plot of apparent decay constant (*k*_app_) extracted from an exponential decay fit of the curves shown in panel A *vs.* inhibitor concentration with the hyperbolic fit shown as a dotted line. Each point is the average of two measurements.

**Fig. 2:**
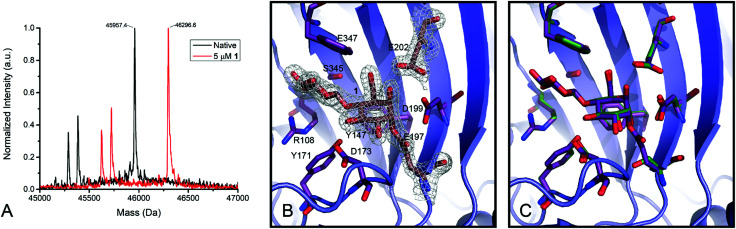
Labelling of HiCel7B with **1**. (A) Deconvoluted intact mass spectra of HiCel7B with (red) and without (black) incubation with **1**. Δm (expected) = 338, Δ*m* (observed) = 339. (B) Structure of the HiCel7B:**1** complex showing 2*F*_o_–*F*_c_ electron density at 2*σ*. Amino acids having polar or hydrophobic contacts with **1** are shown as purple sticks, **1**, E197, and E202 are shown as tan sticks. (C) Overlay of **1** complex with Cellobiose:HiCel7B(E197S) complex (PDB ID: 1OJK). Active site residues and cellobiose from PDB ID 1OJK are shown in green, active site residues and **1** from PDB ID 6YOZ (this work) are shown in purple.

Compound **1** proved to be an efficient covalent inhibitor of HiCel7B, with a *k*_i_/*K*_I_ of 450 M^−1^ s^−1^ (Fig. 1 and Table 1). Intact MS confirmed complete, single labelling after 60 minutes at 25 °C (Fig. 2A). These kinetics compare favourably with the reported requirement to incubate *F. oxysporum* EG I with 8.25 mmol of 3,4-epoxybutyl β-d-cellobioside for 3 hours at 40 °C to achieve complete inhibition.^36^

The addition of another β-1,4-linked glucose residue to the non-reducing terminus to give **5** improved the performance of the inhibitor roughly 7-fold (Table 1 and Fig. S3, ESI†), however intact MS with 2.2 μM enzyme and 5 μM inhibitor revealed minimal labelling (Fig. S4, ESI†). Treatment with **5** gave small peaks with mass differences of both ∼338 Da (equivalent to addition of **1**) and ∼500 Da (expected). Soaking HiCel7B crystals with 0.1 mM **5** also gave an unliganded enzyme structure. Repeating the intact MS experiment with a higher inhibitor concentration (50 μM) resulted in more overall labelling, but still a dominant mass difference of ∼338 Da. Lowering the inhibitor (5 μM) and enzyme concentrations (0.5 μM) gave overall weaker signal, showing incomplete labelling, with a mass difference attributable to **5** as the dominant modification (Fig. S4, ESI†). We interpret these results as indicative of an internal hydrolysis of **5** to give primarily a mixture of cellobiose and cyclophellitol, which is unreactive, and secondarily a mixture of **1** and glucose, which gives rise to the smaller observed mass difference. The observed concentration-dependence suggests that both hydrolytic processes have a higher *K*_M_ than the *K*_I_ of the interaction between HiCel7B and **5**. Thus, the course of inhibition of HiCel7B with **5** is enzyme- and inhibitor concentration-dependent, being affected by the *K*_M_ of the two possible hydrolytic pathways and the *K*_I_ values for **5** and **1**. To hopefully avoid the complication of internal hydrolysis, **13** was built on a β-1,4-glucosyl cyclophellitol inhibitor core.

Probe **13** turned out to be a strong inhibitor of HiCel7B, reacting with a *k*_i_/*K*_I_ of 2100 M^−1^ s^−1^, comparable to that of the **5**. Intact MS confirmed complete single labelling at a 5 : 2.2 probe : enzyme stoichiometric ratio (Fig. S4, ESI†), confirming efficient labelling without hydrolysis. Modifying the azide handle of **13** with Cy5 gave compound **14**, which is an effective probe for in-gel fluorescence-based detection of HiCel7B. A serial dilution of HiCel7B with **14** gave significant signal for the HiCel7B band from as little as 1.6 pg of enzyme per well (Fig. S5, ESI†). Probe **14** also facilitated measurement of the pH-labelling profile for HiCel7B (Fig. S6A, ESI†). Comparison to the pH-activity profile for the hydrolysis of 4MU-GG shows significant similarity between the pH-labelling profile and pH-activity profile, particularly above pH 5 (Fig. S6B, ESI†).

### The structure of the HiCel7B complex with inhibitor **1**

Soaking HiCel7B crystals with **1** yielded the complex shown in Fig. 2B. This confirmed that **1** binds in the expected manner, mimicking the ^4^C_1_ conformation of two glucose units of cellobiose previously observed in the −1 and −2 subsites (Fig. 2C).^37^ Binding of the inhibitor had no significant impact on the structure of the active site, inducing no conformational change following addition to the catalytic nucleophile. This is in spite of the epoxide oxygen forming an extremely close (2.3 Å) contact with the general acid/base. Extending beyond the −2 subsite, in which essential hydrophobic stacking with W347 and hydrogen bonding interactions with R108, Y147, and S345 are formed, the active site broadens significantly, suggesting a lack of a specific −3 subsite, possibly accounting for the weak selectivity between **5** and **13**.

### Enzyme detection and identification by in-gel fluorescence and biotin–avidin enrichment

**Fig. 3:**
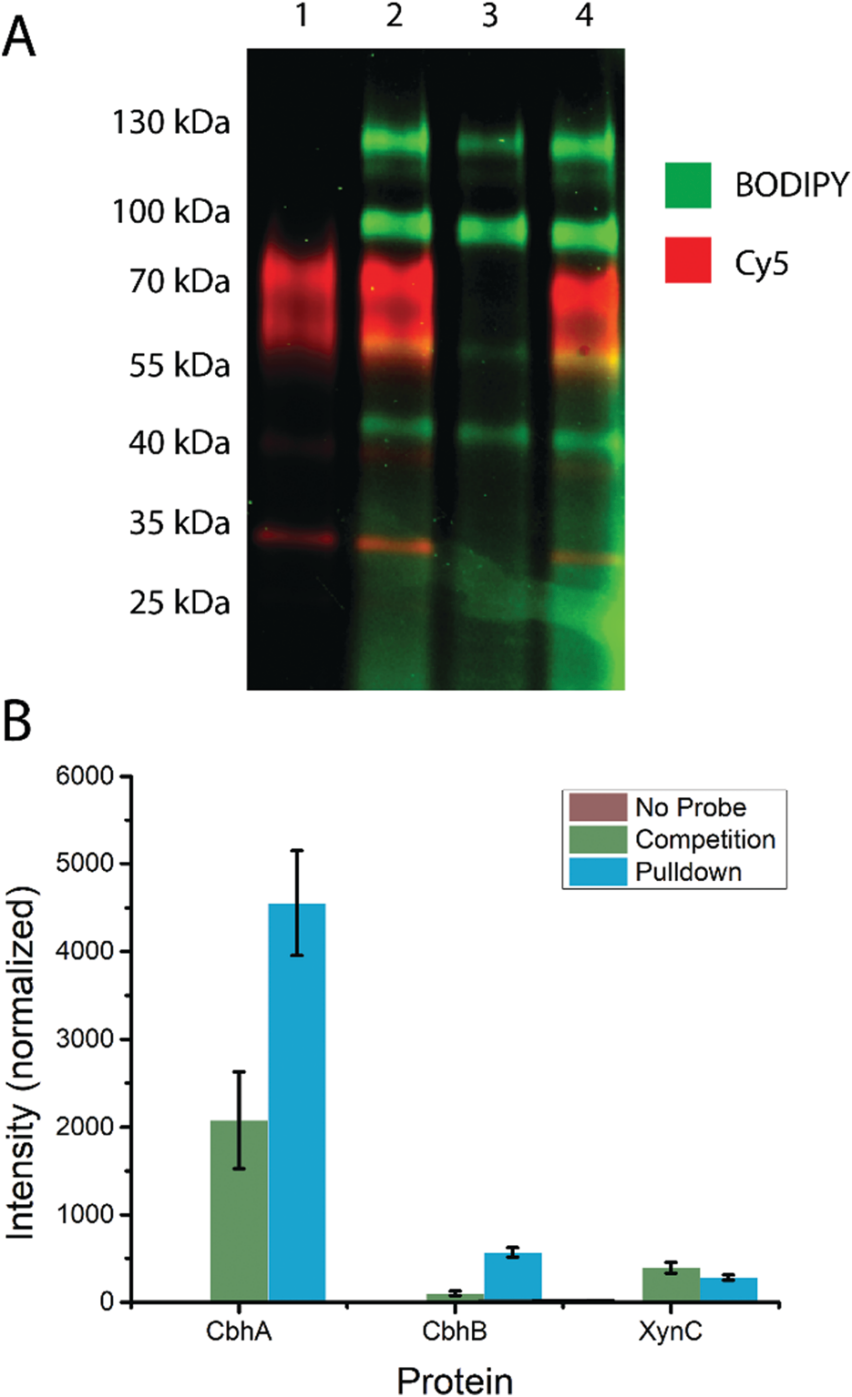
(A) Staining of the A. niger xylan-grown secretome with **14** and **19**. Dual channel fluorescence scan of a 4–20% SDS-PAGE is shown. Lane 1 contains **14**-stained secretome, lane 2 contains **14**-stained secretome subsequently stained with **19**, lane 3 contained **19**-stained secretome, and lane 4 contains **19**-stained secretome subsequently stained with **14**. (B) Label-free quantification of proteins identified from the *A. niger* secretome following treatment with probe **15** and biotin–avidin enrichment. The “No Probe” sample is the negative control, the “Competition” sample was pre-treated with inhibitor **1**, and the “Pulldown” sample was only treated with probe **15**. The observed signal intensity was normalised to a 10 fmol μl^−1^ trysinised yeast enolase internal standard added to each sample prior to analysis.

To test the ability of the probe to stain β-d-glucanases in fungal secretomes without staining β-d-glucosidases, an *A. niger* xylan-grown secretome was stained with **14** or **19**, followed by **19** or **14**, respectively (Fig. 3A). **19**-stained bands were present at molecular weights of ∼45 kDa, ∼60 kDa, ∼100 kDa, and ∼130 kDa. **14**-stained bands were present at ∼35 kDa, ∼40 kDa (faint), ∼60, and ∼80 kDa. Notably, the gel shows minimal overlap between the staining of the two probes and no apparent preclusion of staining of one probe by the other. This suggests that among the retaining glycoside hydrolases secreted by *A. niger*, there is no hydrolysis of **14** and no cross-reactivity between **14** and **19**. Thus, 4-O substitution appears to reduce cross-reactivity with *exo*-glycosidases compared to the previously reported xylanase probes.^16^

Based on the known content of this secretome, we tentatively assigned the 60 and 80 kDa **14**-stained bands as CbhA and CbhB respectively, two GH7 cellulases.^39^ We also assigned the ∼40 kDa band as EglB,^40^ a GH5 *endo*-β-d-glucanase, and the ∼35 kDa band as XynC, an abundant GH10 xylanase likely stained due to a loose enzyme-substrate specificity comparable to other fungal GH10 xylanases.^41,42^ We tentatively assign the 100 and 130 kDa **19**-stained bands as GH3 enzymes, possibly BglA, BglM, and XlnD,^38^ which have been detected in this secretome previously.^16^

To test the specificity of our cellulase probe architecture, we used the biotinylated derivative (probe **15**) and performed a biotin–avidin pulldown enrichment prior to on-bead digestion, peptide identification, and label-free quantification. Three samples were prepared: a negative control, a probe **15**-treated sample, and a sample treated with probe **15** after treatment with inhibitor **1**. The only proteins from *A. niger* detected at elevated levels in the probe **15**-treated samples relative to the negative control were CbhA, CbhB, and XynC, confirming our assignment of the major bands observed by in-gel fluorescence. Label-free quantification showed a significant drop in CbhA and CbhB signal following treatment with inhibitor **1**, but revealed no significant drop in XynC signal, suggesting that XynC was minimally inhibited (Fig. 3B). Thus, the probe architecture presented here shows specificity towards known GH7 cellulases within the context of a complex fungal secretome.

## Conclusions

β-1,4-Glucanases form the foundations of lignocellulose-degrading systems. Being produced by a wide variety of saprotrophic, symbiotic, and pathogenic microorganisms, the ability to detect small quantities of these enzymes directly offers a variety of opportunities for activity-based protein profiling. We have shown here that a cellobiose-mimicking cyclophellitol derivative is a potent inhibitor of β-1,4-glucanases. We have also shown that extension of this inhibitor from the 4′ position with glucose enhances inhibitor binding, but facilitates inhibitor hydrolysis. Fortuitously, extension with a PEG linker enhances binding without facilitating inhibitor hydrolysis. Furthermore, the addition of a detection tag to the linker gives a potent and selective activity-based probe which can be applied to the direct detection of β-1,4-glucanases within a fungal secretome.

## Data deposition

Coordinates and structure factors have been deposited with the PDB, with accession codes 6OYZ (HiCel7B soaked with **1**) and 6YP1 (HiCel7B Soaked with **13**). Results from the label-free quantitation proteomic experiment have been deposited in the PRIDE database with the accession code PXD019930.

## Author contributions

GJD and HO conceived the study. CB, EP, SPS, JJ, G van der M, and JDCC performed or supervised organic synthesis. BIF performed proteomics. JR, AFJR and GPvanW prepared *Aspergillus* cultures and secretomes. NGSM performed structural biology, intact mass spectrometry, kinetic measurements, and in-gel fluorescence. Manuscript preparation was led by NGSM with help from other authors.

## Conflicts of interest

There are no conflicts to declare.

## Supplementary Material

CB-001-D0CB00045K-s001
