# Correction: Glycosylated cyclophellitol-derived activity-based probes and inhibitors for cellulases

**DOI:** 10.1039/d1cb90031e

**Published:** 2021-10-25

**Authors:** Casper de Boer, Nicholas G. S. McGregor, Evert Peterse, Sybrin P. Schröder, Bogdan I. Florea, Jianbing Jiang, Jos Reijngoud, Arthur F. J. Ram, Gilles P. van Wezel, Gijsbert A. van der Marel, Jeroen D. C. Codée, Herman S. Overkleeft, Gideon J. Davies

**Affiliations:** Leiden Institute of Chemistry, Leiden University Einsteinweg 55 2300 RA Leiden The Netherlands h.s.overkleeft@chem.leidenuniv.nl; York Structural Biology Laboratory, Department of Chemistry, The University of York Heslington York YO10 5DD UK gideon.davies@york.ac.uk; Institute of Biology Leiden, Leiden University Sylviusweg 72 2333 BE Leiden The Netherlands

## Abstract

Correction for ‘Glycosylated cyclophellitol-derived activity-based probes and inhibitors for cellulases’ by Casper de Boer *et al.*, *RSC Chem. Biol.*, 2020, **1**, 148–155, DOI: 10.1039/d0cb00045k.

The authors regret that an incorrect PDB code for the structure of the *Humicola insolens* Cel7B with β–1,4 glucosyl cyclophellitol was given in the Data deposition section of the original article. The correct PDB code is 6YOZ.

The Royal Society of Chemistry apologises for these errors and any consequent inconvenience to authors and readers.

## Supplementary Material

