# Supplementary material for: Glycosylated cyclophellitol-derived activity-based probes and inhibitors for cellulases
Source: RSC Chem Biol. 2020 Jul 28;1(3):148–55. doi: 10.1039/d0cb00045k (PMC8341922; doi:10.1039/d0cb00045k)

Electronic Supplementary Material (ESI) for RSC Chemical Biology.
This journal is © The Royal Society of Chemistry 2020

Supplemental Figures and Tables
for
Glycosylated cyclophellitol-derived activity-based probes and inhibitors for cellulases

Casper de Boer,[†b] Nicholas G. S. McGregor,[†a] Evert Peterse,[b] Sybrin P. Schröder,[b] Bogdan I. Florea,[b] Jianbing Jiang,[b] Jos Reijngoud,[c] Arthur F.J. Ram, [c] Gilles P. van Wezel,[c] Gijsbert A. van der Marel,[b] Jeroen D.C. Codée,[b] Herman S. Overkleeft,[b,*] and Gideon J. Davies[a,*]

[a]York Structural Biology Laboratory, Department of Chemistry, The University of York, Heslington, York, YO10 5DD [b]Leiden Institute of Chemistry, Leiden University, Einsteinweg 55, 2300 RA Leiden, The Netherlands [c]Institute of Biology Leiden, Leiden University, Sylviusweg 72, 2333 BE Leiden, The Netherlands

[†] These authors contributed equally to this work.

*Correspondence should be addressed to GJD: Gideon.davies@york.ac.uk, or HSO: h.s.overkleeft@chem.leidenuniv.nl

Supplemental synthetic protocols and characterisation

General chemical synthesis procedures

All reactions were carried out in oven-dried glassware. Trace amounts of water were removed by co-evaporation with toluene. Reactions were carried out under an atmosphere of nitrogen unless stated otherwise. Tetrahydrofuran (THF), *N,N*-dimethylformamide (DMF) dichloromethane (DCM) and toluene were reagent grade and were stored over molecular sieves before use. Pentane, petroleum ether and diethyl ether used for workup and column chromatography were technical grade and used as received. Ethyl acetate (EtOAc) was distilled under reduced pressure before use. Unless stated otherwise, solvents were removed by rotary evaporation under reduced pressure at 40 °C. Triflic acid anhydride (Tf_2O, Fluorochem Ltd) was distilled over P_2O_5 and stored at -20 °C for no more than 3 months before use. All other chemicals (Acros, Sigma-Aldrich, TCI, Carbosynth, Merck, Boom, Honeywell & Biosolve) were used as received. Reactions were monitored by TLC analysis using Merck aluminum sheets (Silica gel 60 F254) with detection by UV absorption (254 nm) and by spraying with a solution of $(NH_4)_6Mo_7O_{24}\cdot 4H_2O$ (25 g/L) and $(NH_4)_4Ce(SO_4)_4\cdot 2H_2O$ (10 g/L) in 10% sulfuric acid or a solution of $KMnO_4$ (20 g/L) and K_2CO_3 (10 g/L) in water, followed by charring at ~150 °C. Silica gel column chromatography was performed on Screening Devices silica gel 60 (particle size of 40 – 63 µm, pore diameter of 60 Å). Gel filtration was performed on an ÄKTA explorer (GE Healthcare) using a 1.6x60 cm Toyopearl HW-40S resin eluting with a solution of NH_4HCO_3 (150mM) in MilliQ. Fraction monitoring was performed using refractive index. For reversed-phase HPLC purifications an Agilent Technologies 1200 series instrument equipped with a semi-preparative column (Gemini C18, 250 x 10 mm, 5 µm particle size, Phenomenex) was used. ^{1}H and ^{13}C NMR spectra were recorded on a 300/75, 400/100, or a 500/125 or 800/200 MHz spectrometer. Chemical shifts (δ) are given in ppm relative to tetramethylsilane or the residual solvent. Coupling constants are given in Hz. High-resolution mass spectrometry (HRMS) analysis was performed with an LTQ Orbitrap mass spectrometer (Thermo Finnigan), equipped with an electrospray ion source in positive mode (source voltage 3.5 kV, sheath gas flow 10 mL/min, capillary temperature 250 °C) with resolution R = 60000 at m/z 400 (mass range m/z = 150 – 2000) and dioctyl phthalate (m/z = 391.28428) as a "lock mass". The mass spectrometer was calibrated prior to measurements with a standard calibration mixture (Thermo Finnigan).

4-*O*-(2,3,4,6-tetra-*O*-benzoyl-β-D-glucopyranosyl)-2,3,6-tri-*O*-benzoyl-β-D-glucopyranosyl 1-(*N*-phenyl)-2,2,2-trifluoroacetimidate (3)

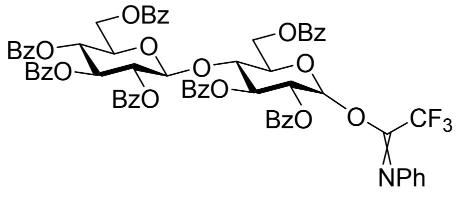

4-*O*-(2,3,4,6-tetra-*O*-benzoyl-β-d-glucopyranosyl)-2,3,6-tri-*O*-benzoyl-β-d-glucopyranose[1] (2.14 g, 2.00 mmol) was dissolved in acetone (13 ml). $CsCO_3$ (0.977 g, 3.0 mmol) and 2,2,2-trifluoro-*N*-phenyl-acetimidoyl chloride (0.39 ml, 2.4 mmol) were added and the reaction was stirred overnight. The mixture was diluted with EtOAc and washed with $NaHCO_3$ (aq. sat) and brine. $MgSO_4$ was added, solids were removed by filtration and the mixture was concentrated under reduced pressure. Column chromatography eluting with pentane/EtOAc (8/2, v/v) yielded the product as a white solid as an E/Z mixture. (2.50 g, 99%)

^1H NMR (300 MHz, $CDCl_3$) δ = 8.17 – 7.88 (m, 18H), 7.84 – 7.71 (m, 7H), 7.67 – 7.48 (m, 6H), 7.48 – 7.12 (m, 33H), 7.12 – 7.00 (m, 3H), 6.95 (t, *J*=7.3, 1H), 6.65 (d, *J*=8.1, 1H), 6.36 (s, 2H), 6.13 (t, *J*=9.1, 1H), 5.78 (td, *J*=9.7, 4.1, 2H), 5.64 – 5.37 (m, 5H), 5.01 (m, 2H), 4.75 – 4.43 (m, 3H), 4.42 – 4.24 (m, 3H), 3.99 – 3.75 (m, 4H). ^{13}C NMR (75 MHz, $CDCl_3$) δ 165.8, 165.5, 165.2, 165.0, 164.9, 142.9, 133.8, 133.5, 133.3, 130.0, 129.9, 129.9, 129.8, 129.8, 129.7, 129.5, 129.4, 128.8, 128.7, 128.6, 128.6, 128.5, 128.5, 128.4, 128.3, 101.2, 76.0, 73.0, 72.7, 72.5, 72.0, 71.5, 70.5, 69.9, 69.4, 62.7, 61.9, 60.5.

4-*O*-(4-*O*-(2,3,4,6-tetra-*O*-benzoyl-β-D-glucopyranosyl)-2,3,6-tri-*O*-benzoyl-β-D-glucopyranosyl)-2,3,6-tri-*O*-benzyl-cyclophellitol (**4**)

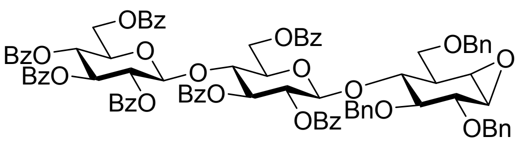

Donor **3** (0.311 g, 0.25 mmol) and acceptor **2** (45 mg, 0.10 mmol) were co-evaporated with toluene (3x). DCM (1.0 ml) and 4Å molecular sieves were added and the mixture was stirred for 30 minutes. The mixture was cooled to −15 °C. TMSOTf (5.4 µl, 0.03 mmol) was added and the mixture was warmed to 0 °C and stirred for 4.5 hours. The reaction was quenched with triethylamine, diluted with DCM and washed with $NaHCO_3$ (aq. sat) and brine. $MgSO_4$ was added, solids were removed by filtration and the mixture was concentrated under reduced pressure. Column chromatography eluting with pentane/EtOAc (8/2 -> 7.5/2.5, v/v) yielded the product as a white solid. (67 mg, 45%)

^1H NMR (400 MHz, $CDCl_3$) δ = 8.01 – 7.89 (m, 10H), 7.79 – 7.69 (m, 4H), 7.60 – 7.13 (m, 36H), 7.01 (t, *J*=7.6, 2H), 6.93 – 6.77 (m, 1H), 5.62 (m, 2H, H3'/H3"), 5.47 (dd, *J*=9.8, 7.9, 1H, H2"), 5.39 (dd, *J*=9.9, 8.0, 1H, H2'), 5.31 (t, *J*=9.5, 1H, H4"), 4.90 (d, *J*=12.0, 1H, CH_2Bn), 4.82 – 4.74 (m, 3H, H1'/H1"/CH_2Bn), 4.68 – 4.53 (m, 2H, CH_2Bn), 4.33 (d, *J*=12.0, 1H, CH_2Bn), 4.25-4.12 (m, 3H, H6a'/H4'/CH_2Bn), 4.08 – 3.96 (m, 2H, H6b'/H6a"), 3.78 (d, *J*=7.4, 1H, H2), 3.73 – 3.53 (m, 4H, H6b"/H4/H5"/H6b), 3.53 – 3.40 (m, 2H, H6a/H3), 3.30 (m, 2H, H5'/epoxide), 3.09 (d, *J*=3.7, 1H, epoxide), 2.26 – 2.09 (m, 1H, H5). ^{13}C NMR (101 MHz, $CDCl_3$) δ 165.8, 165.7, 165.6, 165.5, 165.3, 165.1, 164.8, 139.2, 138.1, 137.7, 133.6, 133.5, 133.5, 133.3, 133.3, 133.2, 123.0, 129.8, 129.8, 129.7, 129.7, 129.7, 129.6, 129.6, 129.3, 128.8, 128.7, 128.7, 128.6, 128.6, 128.6, 128.5, 128.4, 128.4, 128.3, 128.1, 128.0, 127.9, 127.9, 127.7, 127.0, 126.6, 101.4 (C1'), 100.8 (C1"), 83.2 (C3), 79.5 (C2), 76.4 (C4), 76.0 (C4'), 74.3 (CH_2Bn), 73.2 (CH_2Bn), 73.2 (CH_2Bn), 73.1 (C5'), 73.0, 72.9, 72.9 (C3"/C3'/C2'), 72.4 (C5"), 71.9 (C2"), 69.6 (C4"), 68.4 (C6), 62.7 (C6"), 62.3 (C6'), 55.6 (epoxide), 53.3 (epoxide), 41.9 (C5). HRMS (ESI) m/z: $[M+H]^+$ calculated for $C_{89}H_{79}O_{22}$ 1499.5058, found 1499.5058.

4-*O*-(4-*O*-[β-D-glucopyranosyl]-β-D-glucopyranosyl)-cyclophellitol (**5**)

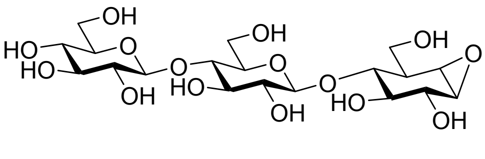

Trisaccharide **4** (64 mg, 0.043 mmol) was dissolved in DCM/MeOH (0.85 ml, 1/1, v/v) NaOMe (4 µl, 5.4M in MeOH) was added and the mixture was stirred overnight. Amberlite CG-50 (NH_4^+) was added until the mixture was no longer strongly alkaline. The resin was filtered off and the solvent was removed under reduced pressure. The residue was dissolved in MeOH (1 ml) and added to cold Et_2O (10ml). The suspension was centrifuged and the solvent was decanted. Subsequently the residue was dissolved in H_2O/MeOH/dioxane (0.4 ml, 1/1/1 v/v). The solution was purged with nitrogen and $Pd(OH)_2/C$ (10 mg) was added. The flask was purged with hydrogen and the reaction was stirred under hydrogen atmosphere 2.5 hours. The flask was purged with nitrogen, solids were removed by filtration over

celite, and the mixture was concentrated in vacuo. Water was added and the sample was lyophilized yielding the product as a white powder (8.5 mg, 40%).

^{1}H NMR (500 MHz, D_2O) δ = 4.55 – 4.48 (m, 2H, H1 2x), 4.09 (dd, *J*=11.3, 3.6, 1H, H6a), 4.00 – 3.91 (m, 3H, H6b/H6a(2x)), 3.90 – 3.81 (m, 2H, H6b), 3.79 – 3.72 (m, 1H, H6b), 3.72 – 3.64 (m, 2H, H3), 3.62 – 3.55 (m, 1H, epoxide), 3.55 – 3.48 (m, 4H, H3 (2x)/H4/H5), 3.44 (m, 1H), 3.42 – 3.36 (m, 1H, H2), 3.33 (dd, *J*=9.3, 7.9, 1H, H2), 3.25 (d, *J*=3.9, 1H, epoxide), 2.37 – 2.29 (m, 1H, H5). ^{13}C NMR (126 MHz, D_2O) δ 102.9 (C1), 102.6 (C1), 78.3, 77.7, 76.0, 75.5, 74.9, 74.8, 74.2, 73.2 (C2 2x), 70.9, 69.5, 60.6 (C6), 59.8 (C6 2x), 56.7 (epoxide), 55.3 (epoxide), 42.8 (C5). HRMS (ESI) m/z: $[M+H]^+$ calculated for $C_{19}H_{33}O_{15}$ 501.1814, found 501.1818.

Methyl 2,3,6-tri-*O*-benzoyl-4-deoxy-4-azido-α-D-glucopyranoside (**7**)

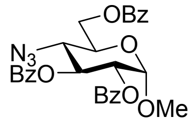

Alcohol **6**[2] (19.0 g, 37.5 mmol) was dissolved in DCM (150 ml, 0.25 M). Pyridine (16.6 ml, 206 mmol) was added and the mixture was cooled to -55 °C. Tf_2O (8.84 ml, 52.52 mmol) was added and the mixture was slowly warmed to room temperature. When TLC (8/2, v/v, Pentane/EtOAc) indicated complete consumption of the starting material, water and DCM were added and the organic layer was washed twice with brine, dried over $MgSO_4$ and filtered. The volatiles were removed under reduced pressure and the crude triflate was dissolved in DMF (125 ml, 0.3 M). NaN_3 (4.88 g, 9.76 mmol) was added and the mixture was stirred overnight at 80 °C. The mixture was allowed to cool to room temperature and was poured over $NaHCO_3$ (aq, sat.). The water layer was extracted three times with EtOAc. The combined organic layers were washed subsequently with $NaHCO_3$ (aq, sat.) and brine, dried with $MgSO_4$ and filtered. Volatiles were removed under reduced pressure and the product was isolated after column chromatography (9/1, v/v, pentane/EtOAc) as a colorless oil (17.9 g, 90%).

^{1}H NMR (400 MHz, $CDCl_3$) δ = 8.16 – 8.09 (m, 2H), 8.05 – 7.96 (m, 4H), 7.62 – 7.53 (m, 1H), 7.52 – 7.42 (m, 4H), 7.39 – 7.29 (m, 4H), 6.06 (t, *J* = 9.9 Hz, 1H, H3), 5.27 (dd, *J* = 10.1, 3.6 Hz, 1H, H2), 5.20 (d, *J* = 3.6 Hz, 1H, H1), 4.74 (dd, *J* = 12.2, 2.4 Hz, 1H, H6a), 4.66 (dd, *J* = 12.2, 4.7 Hz, 1H, H6b), 4.15 – 4.04 (m, 1H, H5), 3.95 (t, *J* = 10.1 Hz, 1H, H4), 3.42 (s, 3H, OMe). ^{13}C NMR (101 MHz, $CDCl_3$) δ = 166.1, 165.8, 165.5, 133.4, 133.4, 133.3, 129.9, 129.7, 129.7, 129.0, 128.8, 128.5, 128.4, 97.1 (C1), 71.9 (C2), 71.1 (C3), 68.0 (5), 63.3 (C6), 61.0 (C4), 55.6 (OMe). HRMS (ESI) m/z: $[M+Na]^+$ calculated for $C_{28}H_{25}N_3O_8Na$ 554.1539, found 554.1542

Acetyl 2,3,6-tri-*O*-benzoyl-4-deoxy-4-azido-a-D-glucopyranoside (8)

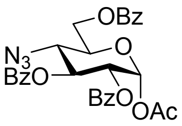

7 (17.88 g, 33.64 mmol) was dissolved in Ac_2O (63.5 ml, 673 mmol). The mixture was cooled to 0°C and AcOH (9.24 ml, 161 mmol) and H_2SO_4 (1.79 ml, 33.6 mmol) were added slowly. The mixture was allowed to warm to rt overnight. TLC (9/1, v/v, pentane/EtOAc) showed full conversion to a lower running spot. $NaHCO_3$ (aq. sat.) was added slowly and the water layer was extracted with toluene three times. The combined organic layers were washed with $NaHCO_3$ (aq. sat.) and brine, dried with $MgSO_4$, filtered and the volatiles were removed under reduced pressure.

^{1}H NMR (300 MHz, $CDCl_3$) δ 8.14 – 8.08 (m, 2H), 8.04 – 7.98 (m, 2H), 7.94 – 7.87 (m, 2H), 7.64 – 7.46 (m, 5H), 7.43 – 7.33 (m, 4H), 6.57 (d, *J* = 3.7 Hz, 1H, H1), 6.00 (dd, *J* = 10.8, 9.2 Hz, 1H, H3), 5.43 (dd, *J* = 10.2, 3.7 Hz, 1H, H2), 4.67 (d, *J* = 3.0 Hz, 2H, H6ab), 4.16 (dt, *J* = 10.5, 3.0 Hz, 1H, H5), 4.00 (t, *J* = 10.1

Hz, 1H, H4), 2.18 (s, 3H, OAc). ^{13}C NMR (75 MHz, $CDCl_3$) δ = 168.7, 166.2, 165.7, 165.5, 133.7, 133.7, 133.5, 129.9, 129.9, 129.6, 128.8, 128.7, 128.6, 128.6, 89.4 (C1), 70.9 (C3), 70.7 (C5), 70.3 (C2), 62.9 (C6), 60.5 (C4), 20.9 (OAc).

Phenyl 2,3,6-tri-*O*-benzoyl-4-deoxy-4-azido-1-thio-β-D-glucopyranoside (9)

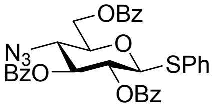

Crude **8** was dissolved in DCM (85ml, 0.4 M) and thiophenol (4.12 ml, 40.4 mmol) and $BF_3 \cdot Et_2O$ (4.98 ml, 40.4 mmol) were added and the reaction was stirred for 40 hours. Thiophenol (2.05 ml, 20 mmol) and $BF_3.Et_2O$ (4.98 ml, 40.4 mmol) were added and the reaction was stirred for another 2 hours. The reaction was quenched with $NaHCO_3$ (aq. sat.) and the organic layer was washed with $NaHCO_3$ (aq. sat.) and brine and was subsequently dried over $MgSO_4$ and filtered. The volatiles were removed under reduced pressure the product was crystalized out of Et_2O and pentane as a white solid. (15.47 mmol, 46%).

^{1}H NMR (400 MHz, $CDCl_3$) δ = 8.11 – 8.07 (m, 2H), 7.95 (m, 4H), 7.68 – 7.59 (m, 1H), 7.57 – 7.33 (m, 10H), 7.29 – 7.22 (m, 1H), 7.19 – 7.10 (m, 2H), 5.70 (t, J = 9.5 Hz, 1H, H3), 5.36 (dd, J = 10.0, 9.4 Hz, 1H, H2), 4.95 (d, J = 10.0 Hz, 1H, H1), 4.81 (dd, J = 12.1, 1.9 Hz, 1H, H6a), 4.59 (dd, J = 12.1, 4.8 Hz, 1H, H6b), 3.92 – 3.76 (m, 2H, H4/H5). ^{13}C NMR (101 MHz, $CDCl_3$) δ = 166.2, 165.7, 165.3, 133.7, 133.5, 133.5, 131.5, 130.0, 130.0, 130.0, 129.7, 129.1, 129.0, 128.8, 128.7, 128.6, 128.6, 128.5, 86.2 (C1), 76.6 (C5), 75.1 (C3), 70.4 (C2), 63.6 (C6), 60.8 (C4). HRMS (ESI) m/z: $[M+Na]^+$ calculated for $C_{33}H_{27}N_3O_7SNa$ 632.1467, found 632.1467

4-*O*-(2,3,6-tri-*O*-benzoyl-4-deoxy-4-azido-β-D-glucopyranosyl)-2,3,6-tri-*O*-benzyl-cyclophellitol (10)

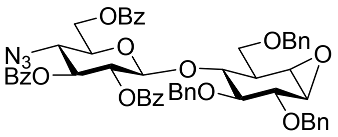

A mixture of **9** (150 mg, 0.246 mmol), Ph_2SO (68 mg, 0.336 mmol) and TTBP (303 mg, 1.22 mmol) was co-evaporated twice with dry toluene and dissolved in dry DCM (2.5 ml). Crushed 3Å molecular sieves were added and the mixture was stirred for 45 min at room temperature. The mixture was cooled to -60 °C and freshly distilled Tf_2O (49 µl, 0.291 mmol) was added. The mixture was allowed to warm to -40 °C within 30 minutes and was subsequently cooled back to -70 °C. **2** (100 mg, 0.224 mmol, co-evaporated three times with dry toluene), dissolved in DCM (1.0 mL), was added. The mixture was slowly warmed to room temperature overnight. Pyridine (0.05 ml) was added and the mixture was poured over brine. DCM was added and the layers were separated. The organic layer was washed with brine, dried with $MgSO_4$, and filtered. The volatiles were evaporated under reduced pressure and the product was isolated by column chromatography (pentane/EtOAc, 9/1 -> 8/2, v/v), providing the product (136 mg, 64%)

^{1}H NMR (500 MHz, $CDCl_3$) δ = 8.04 – 8.00 (m, 2H), 7.92 (m, 4H), 7.61 – 7.12 (m, 24H), 5.50 (t, J=9.8, 1H, H3'), 5.35 (dd, J=9.8, 8.0, 1H, H2'), 4.97 (d, J=11.9, 1H, CH_2Bn), 4.86 – 4.79 (m, 2H, CH_2Bn/H1'), 4.66 (d, J=11.5, 1H, CH_2Bn), 4.61 (d, J=11.5, 1H, CH_2Bn), 4.40 – 4.27 (m, 2H, CH_2Bn/H6a'), 4.24 (dd, J=12.2, 4.2, 1H, H6b'), 4.17 (d, J=11.9, 1H, CH_2Bn), 3.82 (d, J=7.4, 1H, H2), 3.78 (t, J=10.0, 1H, H4'), 3.68 (t, J=9.8, 1H, H4), 3.60 (dd, J=8.8, 3.4, 1H, H6a), 3.52 (dd, J=9.6, 7.4, 1H, H3), 3.46 (t, J=8.5, 1H, H6b), 3.34 – 3.29 (m, 2H, H5'/epoxide), 3.11 (d, J = 3.7, 1H, epoxide), 2.22 – 2.14 (m, 1H, H5). ^{13}C NMR (126 MHz, $CDCl_3$) δ 166.1, 165.7, 165.3, 139.3, 138.2, 137.7, 133.6, 133.3, 129.9, 129.9, 129.7, 129.1, 128.9, 128.7, 128.6, 128.3, 128.0, 127.9, 127.7, 127.3, 126.8, 101.5 (C1'), 83.2 (C3), 79.5 (C2), 76.5 (C4), 74.4 (CH_2Bn), 74.0 (C3'), 73.2 (CH_2Bn), 73.1 (CH_2Bn), 72.9 (C2'), 72.6 (C5'), 68.4 (C6), 63.2 (C6'), 60.9 (C4'), 55.6 (epoxide),

53.3 (epoxide), 41.9 (C5). HRMS (ESI) m/z: [M+Na]+ calculated for $C_{55}H_{51}N_3O_{12}Na$ 968.3365, found 968.3387

4-*O*-(4-deoxy-4-azido-β-D-glucopyranosyl)-2,3,6-tri-*O*-benzyl-cyclophellitol (**11**)

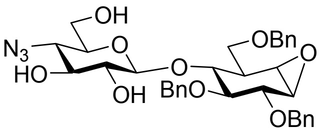

10 (62 mg, 0.098 mmol) was dissolved in a mixture of DCM and methanol (1.3 ml, 1/1, v/v). NaOMe (12 µl of a 5.4 M solution, 0.065 mmol) was added and the mixture was stirred overnight. The reaction was quenched by adding solid CO_2. Volatiles were removed under reduced pressure and column chromatography (EtOAc/DCM, 1/4, v/v) provided the product (25 mg, 60%)

^{1}H NMR (500 MHz, $CDCl_3$) δ 7.41 – 7.24 (m, 15H), 4.87 – 4.79 (m, 2H, CH_2Bn (2x)), 4.77 (d, *J* = 11.4 Hz, 1H, CH_2Bn), 4.69 (d, *J* = 11.3 Hz, 1H, CH_2Bn), 4.65 – 4.56 (m, 2H, CH_2Bn), 4.37 (d, *J* = 7.8 Hz, 1H, H1'), 3.90 – 3.80 (m, 3H, H2/H6ab), 3.78 – 3.71 (m, 2H,H4/OH), 3.56 – 3.43 (m, 3H, H3/H3'/H6a'), 3.37 – 3.29 (m, 3H, H4'/H6b'/epoxide), 3.26 (m, 1H, H2'), 3.17 (d, *J* = 3.7 Hz, 1H, epoxide), 2.91 (m, 1H, H5'), 2.79 (d, *J* = 2.9 Hz, 1H, OH), 2.38 – 2.27 (m, 1H, H5), 1.87 (s, 1H, OH). ^{13}C NMR (126 MHz, $CDCl_3$) δ 138.7, 137.5, 137.4, 128.7, 128.2, 128.2, 128.2, 128.1, 127.8, 127.0, 102.4 (C1'), 82.9 (C3), 79.5 (C2), 75.8 (C3'), 75.1 (C4), 74.9 (C5'), 74.6 (C2'), 74.6 (CH_2Bn), 73.6 (CH_2Bn), 73.2 (CH_2Bn), 69.1 (C6), 61.9 (C6'), 61.1 (C4'), 56.2 (epoxide), 53.2 (epoxide), 42.3 (C5). HRMS (ESI) m/z: [M+Na]+ calculated for $C_{34}H_{39}N_3O_9Na$ 656.2579, found 656.2594

4-*O*-(4-deoxy-4-amino-β-D-glucopyranosyl)-cyclophellitol (12)

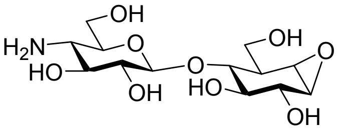

Ammonia (3 ml) was condensed at -50 °C. Sodium (36 mg, 1.57 mmol) was added. **11** (33 mg, 0.052 mmol) was dissolved in THF (3 ml) and *t*-BuOH (0.15 ml, 1.57 mmol). This solution was added dropwise at - 60 °C. After 45 minutes the reaction was quenched with NH_4Cl (97 mg, 1.82 mmol). The mixture was warmed to room temperature and the ammonia was evaporated. The crude product was purified by size exclusion chromatography over HW-40 eluting with 150 mM NH_4HCO_3 in H_2O yielding the product as a white solid (9.3 mg, 53%).

^{1}H NMR (400 MHz, D_2O) δ 4.42 (d, *J* = 7.8 Hz, 1H, H1'), 4.04 (dd, *J* = 11.3, 3.6 Hz, 1H, H6a), 3.94 – 3.78 (m, 3H, H6b/H6a'/H2), 3.70 (t, *J* = 12.5, 5.3 Hz, 1H, H6b'), 3.52 (m, 1H, epoxide), 3.49 – 3.45 (m, 2H, H4/H3), 3.44 – 3.34 (m, 2H, H5'/H3'), 3.29 (dd, *J* = 9.3, 7.7 Hz, 1H, H2'), 3.20 (d, *J* = 3.8 Hz, 1H, epoxide), 2.76 (t, *J* = 9.8 Hz, 1H, H4'), 2.28 (m, 1H, H5). ^{13}C NMR (101 MHz, D_2O) δ 103.1 (C1'), 77.7 (C4), 76.0 (C5'), 75.0 (C3'), 74.7 (C3), 73.7 (C2'), 70.8 (C2), 60.6 (C6'), 59.7 (C6), 56.5 (epoxide), 55.2 (epoxide), 52.1 (C4'), 42.6 (C5). HRMS (ESI) m/z: [M+H]+ calculated for $C_{13}H_{24}NO_9$ 338.1446, found 338.1453

GG azide probe (13)

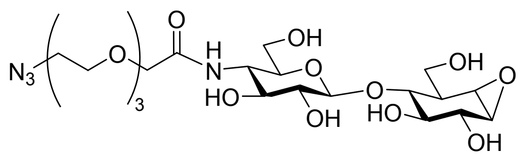

12 (38 mg, 0.113 mmol) was dissolved in DMF (0.4 ml, 0.3 M) and added to PFP ester **17** (59 mg, 0.147 mmol). DIPEA (20 µl, 0.136 mmol) was added and the reaction was stirred overnight. LC/MS indicated full conversion and the product was purified on semi-preparative HPLC eluting with a linear gradient of solution A (MeCN) in solution B (50 mM AcOH in H_2O). The fractions were concentrated under reduced pressure, diluted with water and lyophilized to yield the product as a white solid (16.6 mg, 27%)

^{1}H NMR (500 MHz, D_2O) δ 4.45 (d, *J* = 7.9 Hz, 1H, H1’), 4.11 (s, 2H, $OCH_2C{=}O$), 4.06 (dd, *J* = 11.3, 3.5 Hz, 1H, H6a), 3.91 (dd, *J* = 11.3, 6.7 Hz, 1H, H6b), 3.87 – 3.81 (m, 2H, H2/H4’), 3.75 – 3.67 (m, 11H, TEG (10H)/H6a’), 3.65 (dd, *J* = 10.1, 9.3 Hz, 1H, H3’), 3.60 – 3.56 (m, 2H, H3/H6b’), 3.55 – 3.53 (m, 1H, epoxide), 3.52 – 3.46 (m, 4H, H4/CH_2N_3/H5’), 3.38 (dd, *J* = 9.3, 7.9 Hz, 1H, H2’), 3.21 (d, *J* = 3.9 Hz, 1H, epoxide), 2.33 – 2.27 (m, 1H, H5). ^{13}C NMR (126 MHz, D_2O) δ = 173.2, 103.0 (C1’), 77.7 (C3), 74.8, 74.8 (C5’/C3), 73.8 (C2’), 73.2 (C3’), 70.9 (C2), 70.3, 69.6, 69.5, 69.5, 69.5, 69.3 (OCH_2CH_2O/ O**C**$H_2C{=}O$), 60.6 (C6’), 59.8 (C6), 56.6 (epoxide), 55.3 (epoxide), 51.1 (C4’), 50.2 (CH_2N_3), 42.8 (C5). HRMS (ESI) m/z: $[M+H]^+$ calculated for $C_{21}H_{37}N_4O_{13}$ 553.2352 found 553.2349

GG Cy5 probe (14)

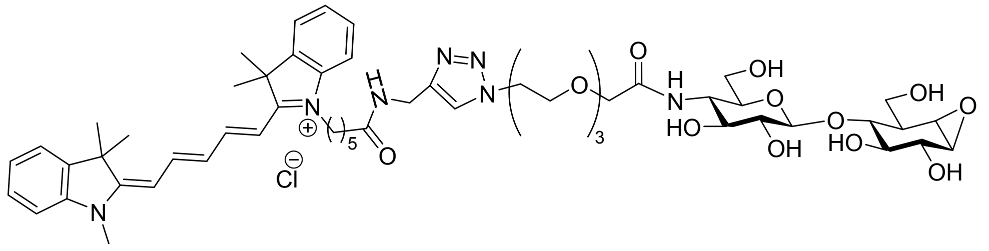

To **13** (3.1 mg, 5.6 µmol) was added a stock solution of DMSO (0.2 ml) containing THPTA (1.68 µmol), CuI (0.56 µmol) and DIPEA (0.67 µmol). To this solution was added Cy5 alkyne (3.3 mg, 5.9 µmol). The mixture was stirred overnight after which LC/MS analysis indicated full consumption of the starting azide. The product was purified on semi-preparative HPLC eluting with a linear gradient of solution A (MeCN) in solution B (50mM NH_4HCO_3 in H_2O). The fractions were concentrated under reduced pressure, diluted with water and lyophilized yielding the compound as a blue solid after lyophilization. (2.8 mg, 46%)

^{1}H NMR (850 MHz, MeOD) δ 8.24 (t, *J* = 12.9 Hz, 2H, alkene), 7.92 (s, 1H, triazole), 7.49 (d, *J* = 7.4 Hz, 2H, phenyl), 7.42 (td, *J* = 7.7, 3.6 Hz, 2H, phenyl), 7.32 – 7.28 (m, 2H, phenyl), 7.28 – 7.25 (m, 2H, phenyl), 6.63 (t, *J* = 12.4 Hz, 1H, alkene), 6.28 (d, *J* = 13.7 Hz, 2H, alkene), 4.58 – 4.56 (m, 2H, OCH_2C**H_2**N), 4.42 (s, 2H, TriazoleC**H_2**NH), 4.36 (d, *J* = 7.9 Hz, 1H, H1’), 4.10 (m, 3H, H6b/$CH_2N{=}C$), 4.01 (s, 2H, O**C**$H_2C{=}O$), 3.90 (t, *J* = 5.1 Hz, 2H, OC**H_2**CH_2N), 3.83 (dd, *J* = 10.9, 7.1 Hz, 1H, H6a), 3.79 – 3.70 (m, 2H, H4’/H2), 3.69 – 3.59 (m, 15H, TEG10H /H6a’/H3’/CH_3N), 3.56 – 3.49 (m, 2H, H6b’/H5), 3.43 – 3.34 (m, 3H, epoxide/H3/H4), 3.30 – 3.27 (m, 1H, H2’), 3.04 (d, J = 3.7 Hz, 1H, epoxide), 2.25 (t, J = 7.4 Hz, 2H, HNC=OCH2), 2.20 – 2.16 (m, 1H, H5), 1.82 (q, J = 7.7 Hz, 2H, HNC=OCH2CH2CH2CH2), 1.75 – 1.67 (m, 14H, HNC=OCH2CH2/CH3 4x), 1.48 (q, J = 7.9 Hz, 2H, HNC=OCH2CH2CH2). 13C NMR (214 MHz, MeOD) δ 175.7, 175.4, 174.6, 173.7, 155.5, 155.5, 146.1, 144.3, 143.6, 142.6, 142.5, 129.8, 129.7, 126.6, 126.3, 126.2, 125.0, 123.4, 123.3, 112.1, 111.8, 104.7 (C1’), 104.4 (Cy), 104.3 (Cy), 80.5 (C4), 77.0 (C5’), 76.9 (C3), 75.8 (C2’), 74.9 (C3’), 72.9 (C2), 71.9 (TEG), 71.4 (TEG 2x), 71.3 (TEG), 71.2 (OCH2C=O), 70.3 (OCH2CH2N), 62.8 (C6’), 62.1 (C6), 56.9 (epoxide), 56.5 (epoxide), 52.9 (C4’), 51.4 (OCH2CH2N), 44.9 (C5), 44.8 (CH2N=C), 36.5 (HNC=OCH2), 35.6 (CH2NH), 31.5 (CH3N), 28.2 (HNC=OCH2CH2CH2CH2), 28.0 (2x CH3Cq), 27.8 (2x CH3Cq), 27.4 (HNC=OCH2CH2CH2), 26.4 (HNC=OCH2CH2CH2). HRMS (ESI) m/z: $[M]^+$ calculated for $C_{56}H_{78}N_7O_{14}$ 1072.5601, found 1072.5618

GG biotin probe (15)

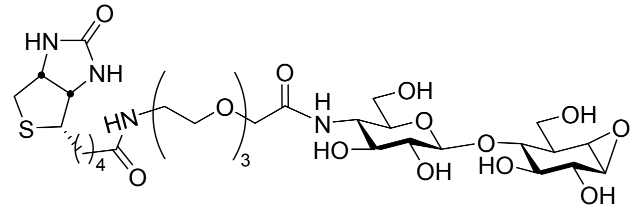

Carboxylic acid **18** (25 μmol) was dissolved in DMF (0.5 ml), 2,3,4,5,6-pentafluorophenol (23 mg, 0.13 μmol), Et_3N (10 μl, 0.13 mmol) and DIC (3.9 μl,25 μmol) were added and the mixture was stirred for 90 minutes. Part of the stock solution (0.34 ml) was added to amine **12** and stirred overnight. LC/MS indicated full conversion of the amine and the product was purified on semi-preparative HPLC eluting with a linear gradient of solution A (MeCN) in solution B (50 mM AcOH in H2O). The fractions were concentrated under reduced pressure, co-evaporated with water, diluted with water and lyophilized to yield the product as a white solid. (4.68 mg, 44%)

^1H NMR (850 MHz, MeOD) δ = 4.50 (dd, *J*=7.9, 4.0, 1H, biotin), 4.36 (d, *J*=7.9, 1H, H1'), 4.31 (dd, *J*=7.9, 4.5, 1H, biotin), 4.12 (dd, *J*=10.9, 4.1, 1H, 6A), 4.05 (d, *J*=2.4, 2H, O**CH₂**C=O), 3.85 (dd, *J*=10.9, 7.1, 1H, 6B), 3.77 – 3.71 (m, 3H), 3.71 – 3.67 (m, 5H), 3.67 – 3.61 (m, 4H), 3.57 – 3.54 (m, 3H), 3.52 (ddd, *J*=10.4, 6.1, 2.1, 1H), 3.42 (dt, *J*=3.7, 1.1, 1H, epox), 3.42 – 3.36 (m, 4H), 3.30 – 3.28 (m, 1H, H2'), 3.22 (ddd, *J*=9.0, 5.9, 4.5, 1H, biotin), 3.05 (d, *J*=3.7, 1H, epoxide), 2.93 (dd, *J*=12.8, 5.0, 1H, biotin), 2.71 (d, *J*=12.7, 1H, biotin), 2.23 (t, *J*=7.4, 1.4, 2H, biotin), 2.21 – 2.17 (m, 1H, H5), 1.77 – 1.71 (m, 1H, biotin), 1.71 – 1.57 (m, 3H, biotin), 1.48 – 1.42 (m, 2H, biotin). ^{13}C NMR (214 MHz, MeOD) δ 176.2, 173.8, 104.7 (C1'), 80.5, 77.0, 76.9, 75.8 (C2'), 74.9, 72.9, 71.8, 71.4, 71.4, 71.2, 71.1, 70.6, 63.4 (biotin), 62.8 (C6'), 62.2 (C6), 61.6 (biotin), 57.0 (biotin), 56.8 (epoxide), 56.5 (epoxide), 52.9, 45.0 (C5), 41.0, 40.3, 36.8, 29.8, 29.5, 26.8. HRMS (ESI) m/z: $[M+H]^+$ calculated for $C_{31}H_{53}N_4O_{15}S$ 753.3223 found 753.3219

COOH-TEG-N₃ (**S1**)

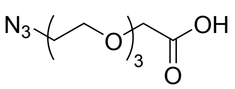

Ester **16**[3] (100 mg, 0.346 mmol) was dissolved in DCM/TFA (7 ml, 1/1, v/v, 0.05 M) and stirred for 30 minutes. The mixture was repeatedly co-evaporated with toluene and used and analyzed without further purification.

^1H NMR (400 MHz, CD_3CN) δ = 4.08 (s, 2H, OC**H₂**COOH), 3.67 – 3.57 (m, 10H), 3.37 (t, *J*=4.9, 2H, CH_2N_3). ^{13}C NMR (101 MHz, CD_3CN) δ 172.2 (COOH), 71.5, 71.1, 71.0, 71.0, 70.5, 68.8, 51.5 (CH_2N_3). HRMS (ESI) m/z: $[M+Na]^+$ calculated for $C_8H_{15}N_3O_5Na$ 256.0904, found 256.0902.

PFP-TEG-N₃ (**17**)

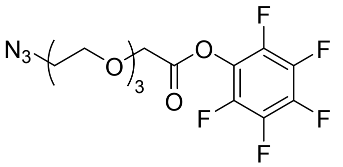

Crude acid **S1** (0.346 mmol) was dissolved in DCM (1.9 ml, 0.2 M). 2,3,4,5,6-pentafluorophenol (70 mg, 0.381 mmol), DIC (0.059 ml, 0.381 mmol) and DMAP (cat.) were added and the mixture was stirred overnight. Volatiles were evaporated under reduced pressure and the mixture was separated by column chromatography (pentane/EtOAc, 9/1 -> 8/2, v/v) providing the product as a colorless oil (93 mg, 67 % over 2 steps).

^1H NMR (400 MHz, $CDCl_3$) δ 4.56 (s, 2H, $OCH_2C=O$), 3.86 – 3.81 (m, 2H), 3.78 – 3.74 (m, 2H), 3.71 – 3.66 (m, 6H), 3.40 (t, *J* = 5.1 Hz, 2H, CH_2N_3). ^{13}C NMR (101 MHz, $CDCl_3$) δ 166.8, 71.4, 70.9, 70.8, 70.8, 70.2, 68.0 (, O**C**H₂C=O), 50.8 (**C**H₂N₃). HRMS (ESI) m/z: $[M+NH_4]^+$ calculated for $C_{14}H_{18}F_5N_4O_5$ 417.1192 found 417.1190

t-Bu-TEG-NH$_2$ (S2)

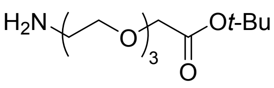

Azide **16**[3] (1.0 g, 3.46 mmol) was dissolved in THF (11.5 ml, 0.3 M), PPh_3 (1.81 g, 6.91 mmol) and H_2O (1.5 ml, 83 mmol) were added and the mixture was stirred 72 h at rt. The mixture was diluted with H_2O (140 ml) and washed with toluene (3 x 50 ml). The combined organic layers were extracted with H_2O (4 x 20 ml), and then the water layers were combined and evaporated. The residual oil was co-evaporated with dioxane (3x) to give the title compound as an oil (803 mg, 88%).

^{1}H NMR (400 MHz, $CDCl_3$) δ 4.03 (s, 2H), 3.79 – 3.61 (m, 8H), 3.52 (t, J = 5.2 Hz, 2H), 2.87 (t, J = 5.2 Hz, 2H), 1.82 (br s, 2H, NH2), 1.48 (s, 9H) ppm. ^{13}C NMR (101 MHz, $CDCl_3$) δ 169.6, 81.5, 73.3, 70.6, 70.5, 70.5, 70.2, 68.9, 41.7, 28.0 ppm HRMS (ESI) m/z: $[M+H]^+$ calculated for $C_{12}H_{26}NO_5$ 264.1806 found 264.1803

t-Bu-TEG-biotin (S3)

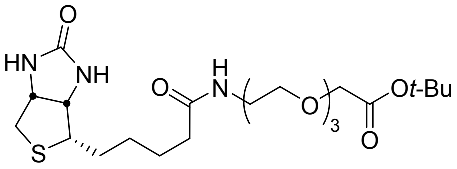

Biotin-NHS[6] (171 mg, 0.5 mmol) was dissolved in dry DMF (1.0 ml, 0.5 M), then DIPEA (105 µl, 0.6 mmol) and amine **S2** (145 mg, 0.55 mmol) were added and the mixture was stirred 16 h at rt. The mixture was evaporated at 60 °C and then flash purification by silica column chromatography (DCM/MeOH, 49/1 -> 9/1) afforded the title compound as a white solid (202 mg, 83%).

^{1}H NMR (400 MHz, $CDCl_3$) δ 7.02 (t, J = 5.2 Hz, 1H, NH), 6.94 (s, 1H, NH), 6.24 (s, 1H, NH), 4.59 – 4.43 (m, 1H), 4.36 – 4.18 (m, 1H), 4.02 (s, 2H), 3.81 – 3.61 (m, 8H), 3.57 (t, J = 4.8 Hz, 2H), 3.51 – 3.33 (m, 2H), 3.14 (q, J = 7.0 Hz, 1H), 2.90 (dd, J = 12.7, 4.6 Hz, 1H), 2.84 – 2.64 (m, 1H), 2.23 (t, J = 7.4 Hz, 2H), 1.80 – 1.60 (m, 4H), 1.48 (s, 11H) ppm. ^{13}C NMR (101 MHz, $CDCl_3$) δ 173.4, 169.5, 164.4, 81.5, 70.5, 70.3, 70.3, 69.9, 69.9, 68.8, 61.7, 60.2, 55.7, 40.4, 39.0, 35.9, 28.3, 28.0, 25.6 ppm. HRMS (ESI) m/z: $[M+H]^+$ calculated for $C_{22}H_{40}N_3O_7S$ 490.2582 found 490.2571

COOH-TEG-biotin (18)

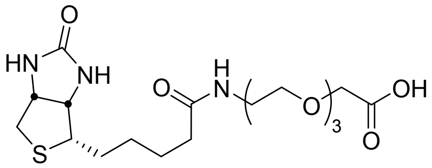

Ester **S3** (195 mg, 0.4 mmol) was dissolved in DCM/TFA (4.0 ml, 0.1 M, 20%). The mixture was stirred for 16 h at rt, subsequently diluted with toluene (20 ml) and concentrated under reduced pressure (3x) to furnish the title product as a white solid (173 mg, quant.).

^{1}H NMR (400 MHz, $CDCl_3$ + 3 drops of CD_3OD) δ 7.13 (s, 1H, NH), 7.00 (s, 1H, NH), 4.55 (dd, J = 7.6, 4.8 Hz, 1H), 4.37 (dd, J = 7.6, 4.5 Hz, 1H), 4.32 – 4.05 (m, 2H), 3.83 – 3.60 (m, 8H), 3.57 (t, J = 4.7 Hz, 2H), 3.52 – 3.33 (m, 2H), 3.18 (q, J = 7.3 Hz, 1H), 2.93 (dd, J = 13.0, 4.8 Hz, 1H), 2.84 – 2.67 (m, 1H), 2.27 (t, J = 7.7 Hz, 2H), 1.80 – 1.58 (m, 4H), 1.51 – 1.39 (m, 2H) ppm. ^{13}C NMR (101 MHz, $CDCl_3$ + CD_3OD) δ 174.7, 174.0, 165.2, 71.0, 70.6, 70.5, 70.1, 69.7, 68.8, 62.2, 60.7, 55.4, 40.5, 39.6, 35.6, 28.1, 27.9, 25.7 ppm. HRMS (ESI) m/z: $[M+H]^+$ calculated for $C_{18}H_{32}N_3O_7S$ 434.19555 found 434.19565

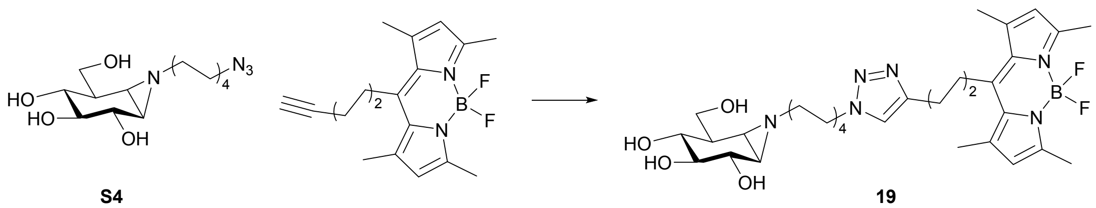

BODIPY Green β-glucosidase probe 19

Azide **S4**[4] (13 mg, 39 μmol) was dissolved in DMF (0.80 mL). Green BODIPY alkyn[5] (15 mg, 45 μmol), $CuSO_4$ (1.0 M in H_2O, 15 μL, 15 μmol) and sodium ascorbate (1.0 M in H_2O, 16 μL, 16 μmol) were added to the solution under argon atmosphere. After stirring at room temperature for 12 h, volatiles were removed under reduced pressure. The product was purified on semi-preparative HPLC eluting with a linear gradient of solution A (MeCN) in solution B (50mM NH_4HCO_3 in H_2O). The fractions were concentrated under reduced pressure, diluted with water and lyophilized yielding the compound as an orange solid after lyophilization. (7.7 mg, 30%)

^1H NMR (400 MHz, Methanol-d_4) δ 7.70 (s, 1H), 6.08 (s, 2H), 4.31 (t, J = 7.0 Hz, 2H), 3.95 (dd, J = 10.1, 4.4 Hz, 1H), 3.61 – 3.52 (m, 2H), 3.07 (dd, J = 9.9, 8.2 Hz, 1H), 2.97 (td, J = 9.2, 8.6, 3.4 Hz, 3H), 2.74 (t, J = 7.2 Hz, 2H), 2.40 (s, 6H), 2.34 (s, 6H), 2.28 (dt, J = 11.7, 7.7 Hz, 1H), 2.08 (dt, J = 11.6, 7.2 Hz, 1H), 1.95 (dd, J = 6.3, 3.5 Hz, 1H), 1.92 – 1.78 (m, 5H), 1.68 – 1.45 (m, 5H), 1.24 (d, J = 13.6 Hz, 8H). ^{13}C NMR (101 MHz, MeOD) δ 154.9, 148.5, 147.9, 142.2, 132.6, 123.4, 122.6, 79.0, 73.9, 70.1, 63.7, 62.1, 51.2, 45.51, 45.45, 43.0, 32.2, 31.2, 30.8, 30.3, 30.2, 29.8, 29.1, 28.2, 27.3, 25.9, 16.5, 14.4. HRMS (ESI) m/z: $[M+H]^+$ calculated for $C_{34}H_{51}NBF_2N_6O_4$ 657.41117, found 657.41122

Supplemental References

1 A. Cammarata, S. K. Upadhyay, B. S. Jursic and D. M. Neumann, *Bioorg. Med. Chem. Lett.*, 2011, **21**, 7379–7386.

2 F. J. Weiberth, H. S. Gill, Y. Jiang, G. E. Lee, P. Lienard, C. Pemberton, M. R. Powers, W. Subotkowski, W. Tomasik, B. J. Vanasse and Y. Yu, *Org. Process Res. Dev.*, 2010, **14**, 623–631.

3 K. Heller, P. Ochtrop, M. F. Albers, F. B. Zauner, A. Itzen and C. Hedberg, *Angew. Chemie Int. Ed.*, 2015, **54**, 10327–10330.

4 J. Jiang, T. J. M. Beenakker, W. W. Kallemeijn, G. A. Van Dermarel, H. Van Den Elst, J. D. C. Codée, J. M. F. G. Aerts and H. S. Overkleeft, *Chem. - A Eur. J.*, 2015, **21**, 10861–10869.

5 M. Verdoes, U. Hillaert, B. I. Florea, M. Sae-Heng, M. D. P. Risseeuw, D. V. Filippov, G. A. van der Marel and H. S. Overkleeft, *Bioorganic Med. Chem. Lett.*, 2007, **17**, 6169–6171.

6 K. Susumu, H. T. Uyeda, I. L. Medintz, T. Pons, J. B. Delehanty, H. Mattoussi, *J. Am. Chem. Soc.*, 2007, **129**, 13987–13996.

Supplemental Figures

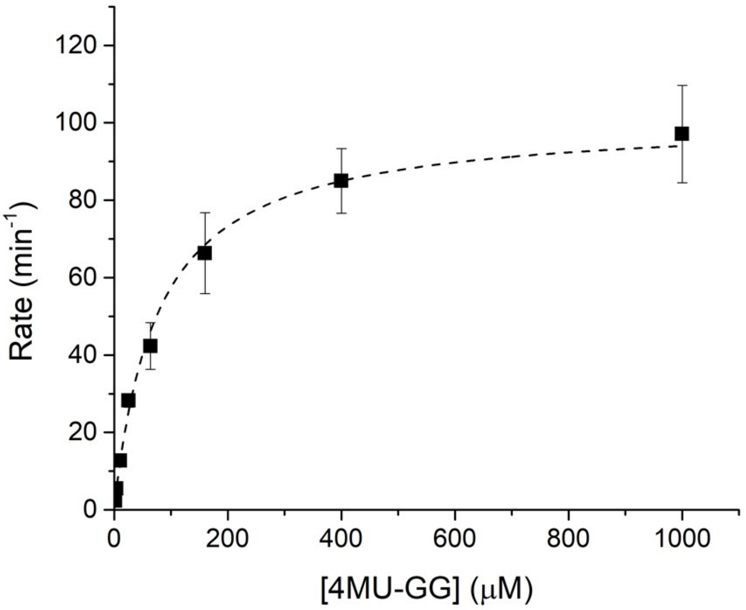

Supplemental Figure 1: Michaelis-Menten curve measured for HiCel7B hydrolyzing 4MU-β-cellobioside in 50 mM pH 7 sodium phosphate buffer at 25°C. Each datapoint represents the average of four initial rate slope measurements with error bars representing the standard deviation. Data were corrected for inner filter effect measured using a 4MU standard curve and data were fit to a standard Michaelis-Menten site-saturation kinetic model.

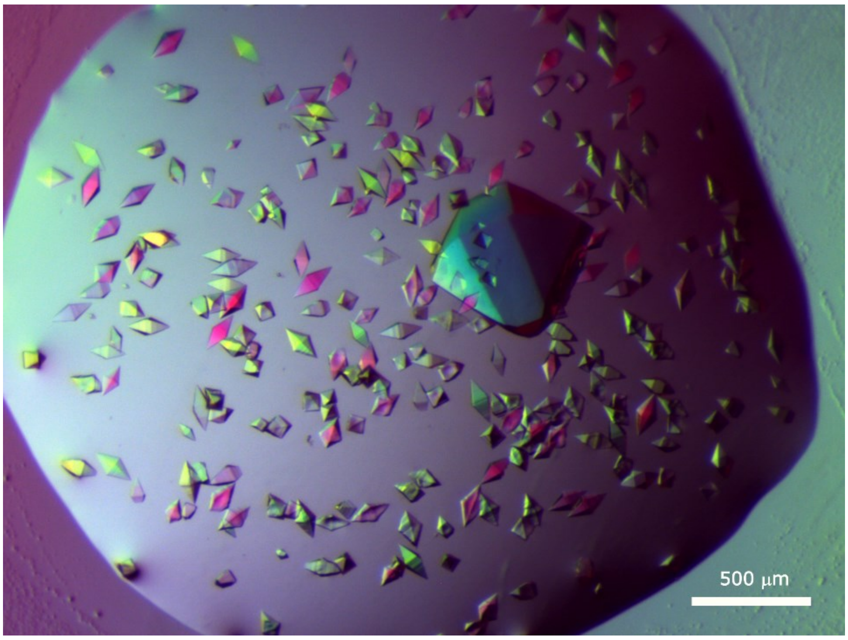

Supplemental Figure 2: A) Crystals of HiCel7B grown at 293 K from a mixture of 1200 nL of 12 mg/mL HiCel7B in 20 mM Tris, pH 8 mixed with 600 nL of 0.15 M trisodium citrate, 1 M Li_2SO_4, 0.8 M $(NH_4)_2SO_4$, viewed under polarized light.

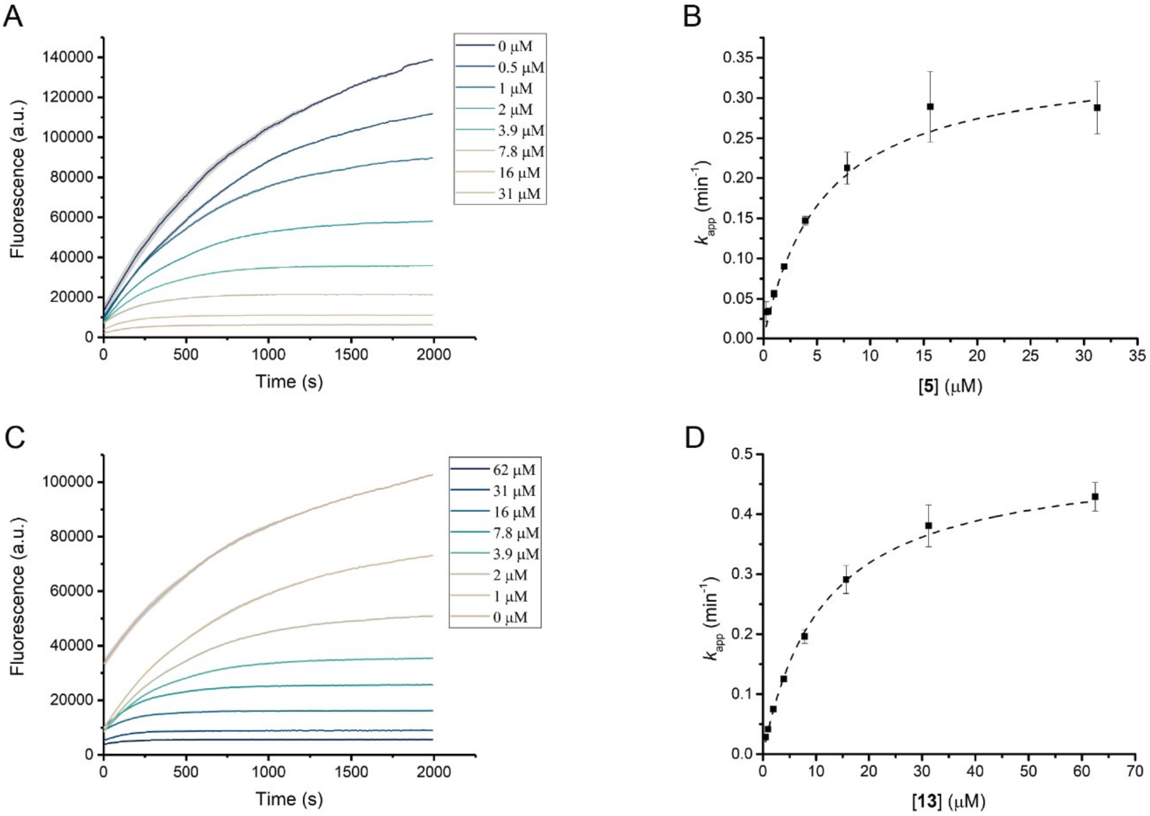

Supplemental Figure 3: Inhibition kinetics of HiCel7B with **5** and **13**. A, C) Plots of fluorescence vs. time for HiCel7B in the presence of difference concentrations of inhibitors **5** or **13**, respectively. The average of four measurements is shown with the standard deviation represented as thinner lines above and below. B, D) Plots of k_{app} vs. [inhibitor] for HiCel7B interacting with **5** or **13**, respectively. Error bars are the standard deviation of the k_{app} values extracted from each of the four measured curves independently.

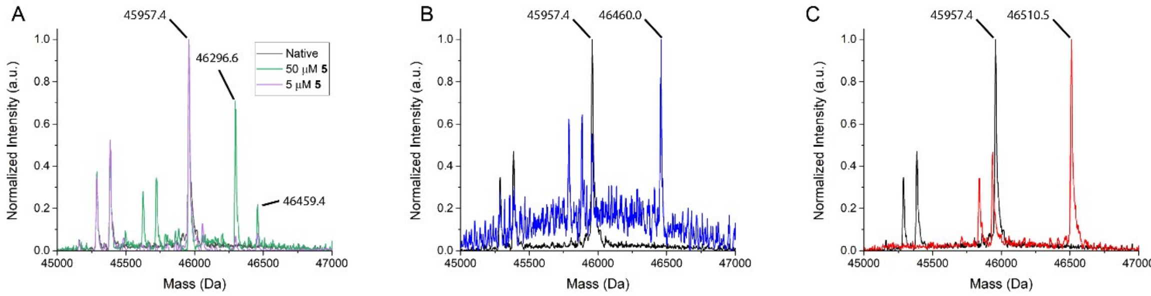

Supplemental Figure 4: Deconvoluted intact mass spectra of HiCel7B before (black) and after (coloured) treatment with **5** (A,B) or **13** (C). A) 2.2 μM HiCel7B treated with 5 μM **5** (purple) or 50 μM **5** (green). Δm (expected) = 500, Δm (observed) = 339, 502. B) 0.5 μM HiCel7B treated with 5 μM **5** (blue). Δm (expected) = 500, Δm (observed) = 503. C) 2.2 μM HiCel7B treated with 5 μM **13**. Δm (expected) = 552, Δm (observed) = 553.

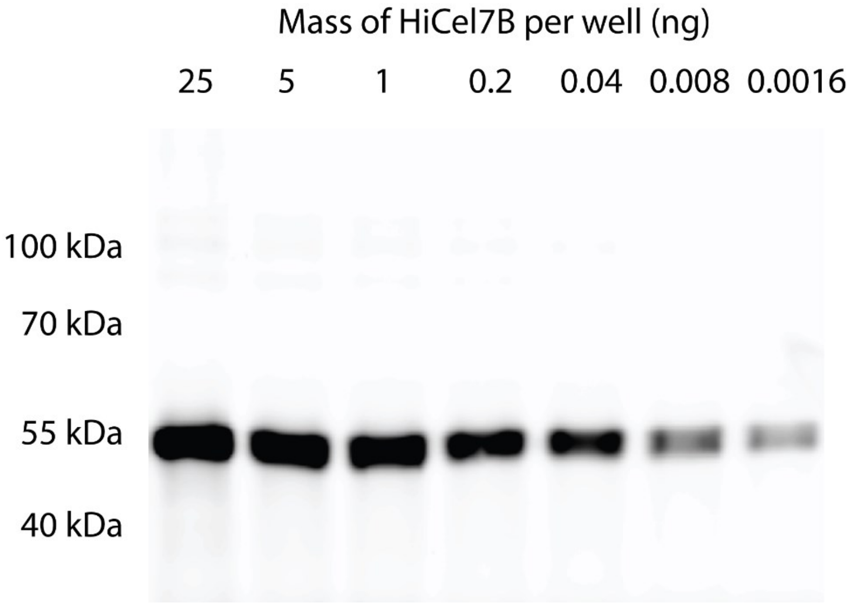

Supplemental Figure 5: Detection of HiCel7B using **14**. Cy5 fluorescence scan of an SDS-PAGE gel run following the staining of variable quantities of HiCel7B. The mass of **14**-stained HiCel7B loaded into each well is given above each well.

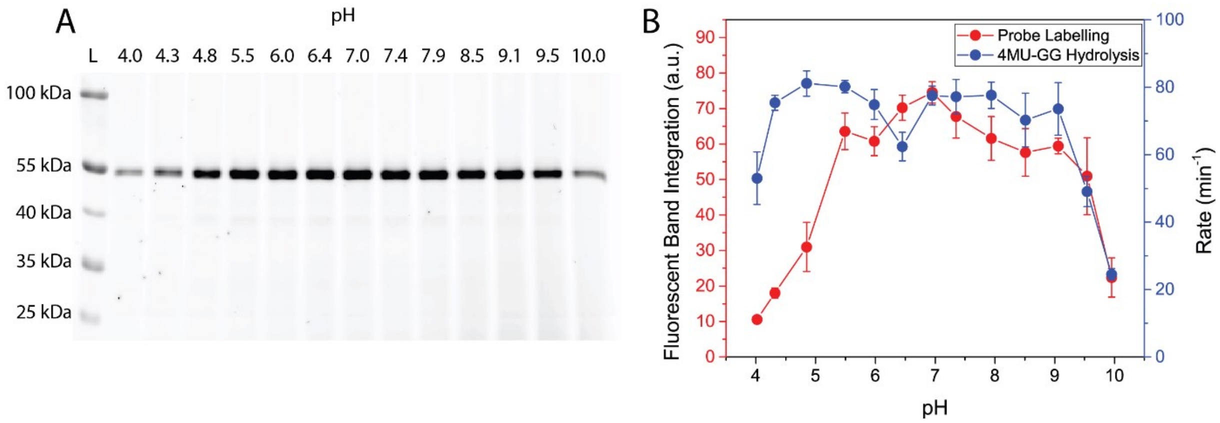

Supplemental Figure 6: pH-dependence of Hicel7B hydrolytic activity and probe labelling. A) Cy5-fluorescence scan of HiCel7B (~7 ng per well) labelled with probe 14 in SPG buffer at pH values given above each lane. B) Plot of 4MU-GG hydrolytic activity vs pH (blue) and Cy5-Fluorescence band integration vs. pH (red) for HiCel7B. Each point represents the average of 4 measurements. Error bars are the standard deviation.

Supplemental Tables

Supplemental Table 1: Data collection and refinement statistics (molecular replacement)

	HiCel7B	
	Complex with **1** (PDB 6YOZ)	Unliganded (soaked with **13**) (PDB 6YP1)
**Data collection**		
Space group	$P4_122$	$P4_22_12$
a, *b*, *c* (Å)	102.00 102.00 278.85	92.31, 92.31, 100.81
α, β, γ (°)	90, 90, 90	90, 90, 90
Resolution (Å)	57.55-1.88 (1.91-1.88)*	46.16-1.20 (1.22-1.20)
R_{meas}	0.177 (2.978)	0.053 (0.483)
$I / \sigma I$	14.2 (1.1)	26.7 (3.2)
Completeness (%)	100 (100)	99.8 (96.3)
Redundancy	16.2 (16.1)	14 (4.7)
**Refinement**		
Resolution (Å)	57.55-1.88 (1.93-1.88)	46.16-1.2 (1.23-1.20)
No. reflections	114251 (8348)	128433 (9151)
R_{work} / R_{free}	0.201/0.236 (0.365/0.406)	0.113/0.132 (0.219/0.249)
No. atoms		
Protein	6093	3142
Ligand/ion	200	126
Water	519	393
B-factors		
Protein	35.8	12.7
Ligand/ion	49.3	27.6
Water	43.6	25.1
R.m.s. deviations		
Bond lengths (Å)	0.0142	0.0238
Bond angles (°)	1.824	2.158

*Values in parentheses are for highest-resolution shell

Supplemental Table 2: Progenesis QI label-free quantification results for proteins pulled down from the *A. niger* secretome

Accession	Common Name	Peptide Count	Anova (p)	Average Abundance (Standard Deviation)		
				No Probe	Pulldown	Competition
NRRL3_02584	CbhB	2	4.78E-08	0 (0)	566 (53)	99.5 (26)
NRRL3_04953	CbhA	3	1.52E-07	0.3 (0.4)	4550 (590)	2080 (550)
NRRL3_08708	XynC	3	2.56E-05	7.8 (4.4)	279 (28)	390 (62)
NRRL3_11298	PNPO	3	0.03368	250 (33)	328 (37)	396 (52)
ENO1_YEAST	Enolase	29	0.045746	9160 (1700)	10800 (2600)	16600 (2300)
TRYP_PIG	Trypsin	3	0.070538	27900 (6200)	30600 (4000)	44400 (5700)
NRRL3_02413	Unknown	3	0.146654	589 (88)	650 (130)	932 (240)
AVID_CHICK	Avidin	11	0.877625	15500 (8900)	10600 (2300)	12200 (4200)

Compound 3 in $CDCl_3$

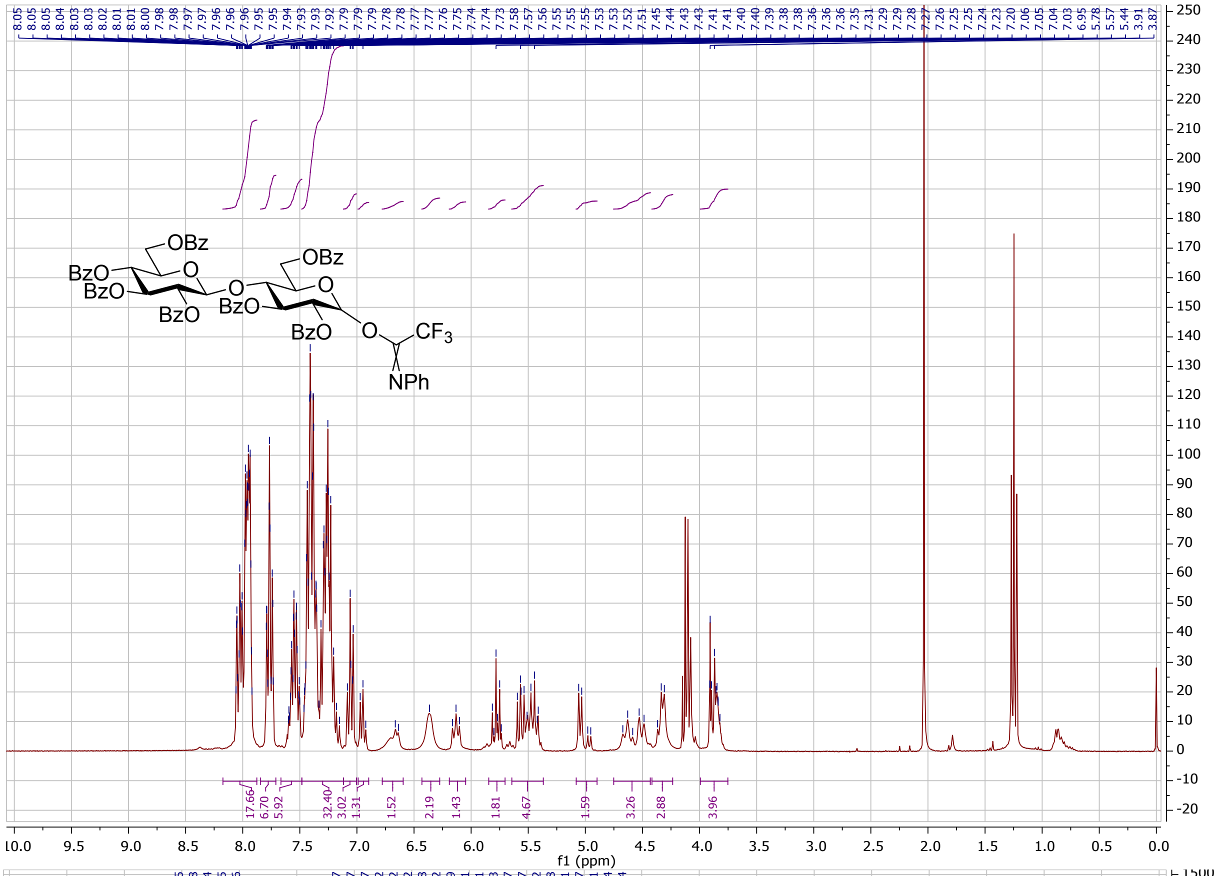

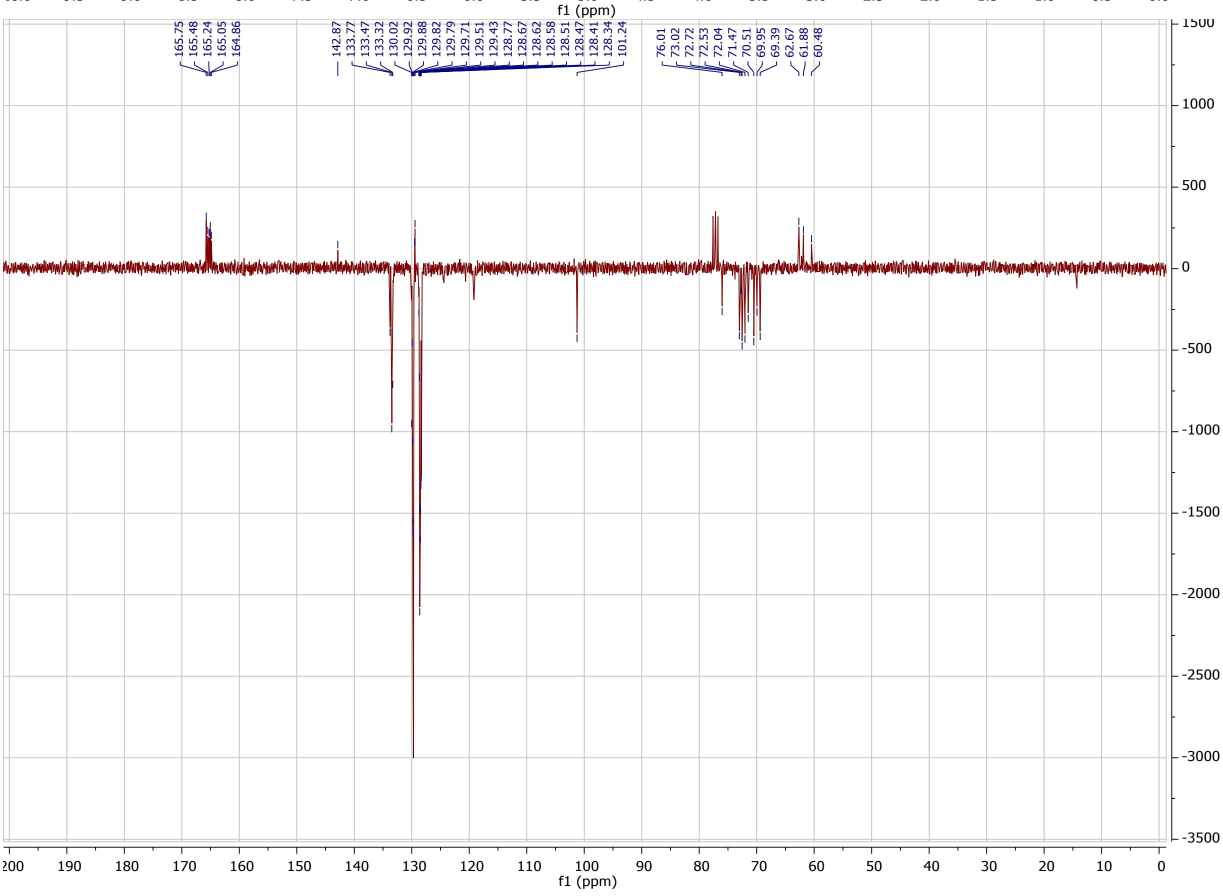

Compound 4 in $CDCl_3$

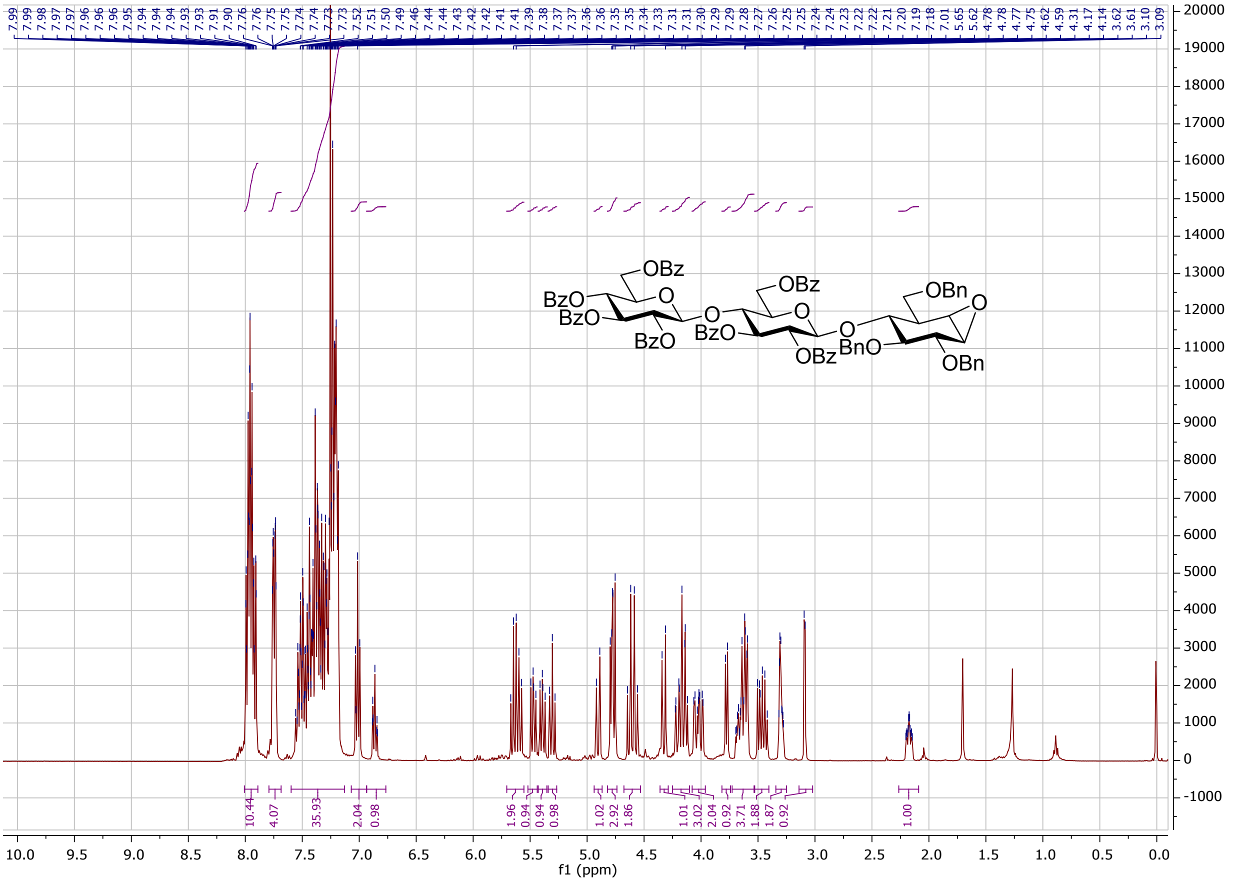
OBz
BzO
BzO
BzO
O
OBz
BzO
OBz
OBn
BnO
OBn
f1 (ppm)

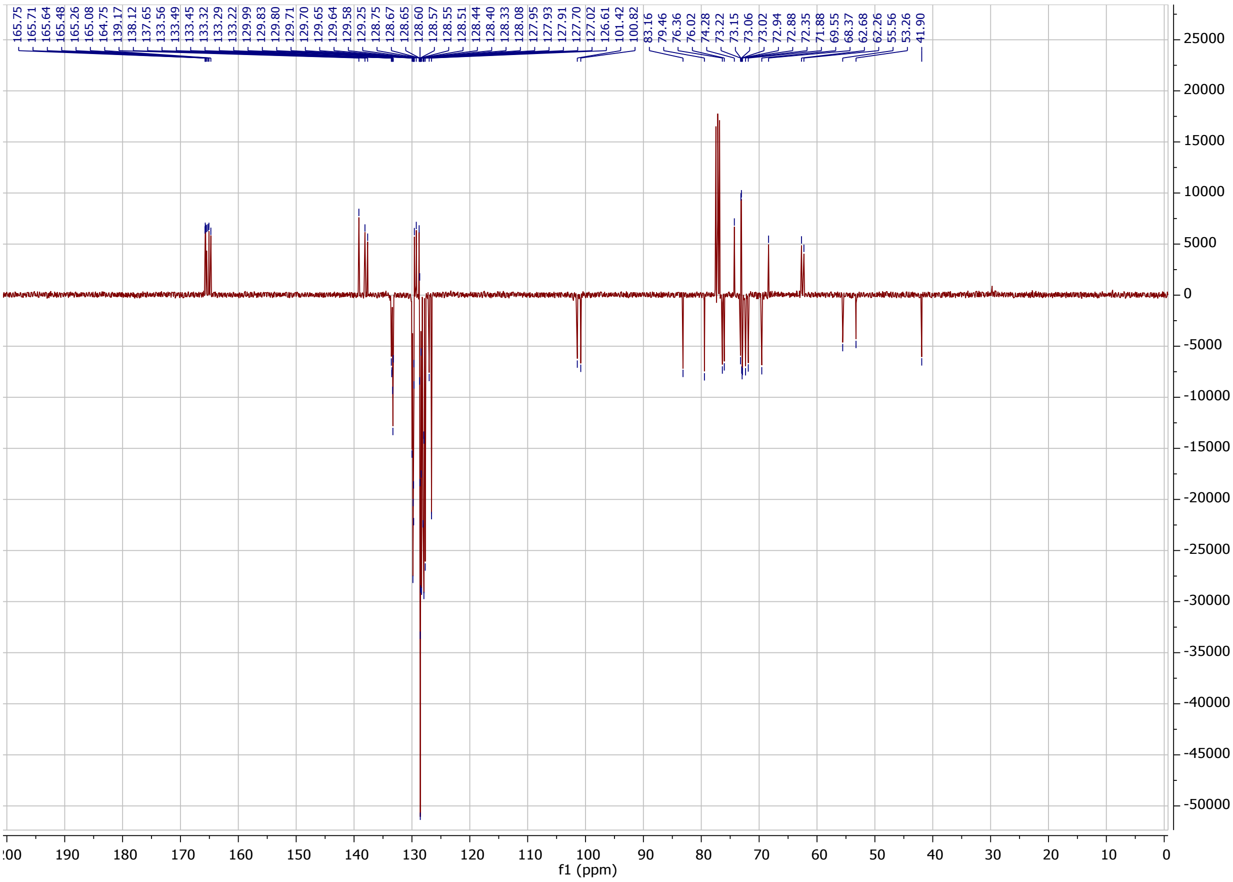
f1 (ppm)

Compound 5 in D_2O

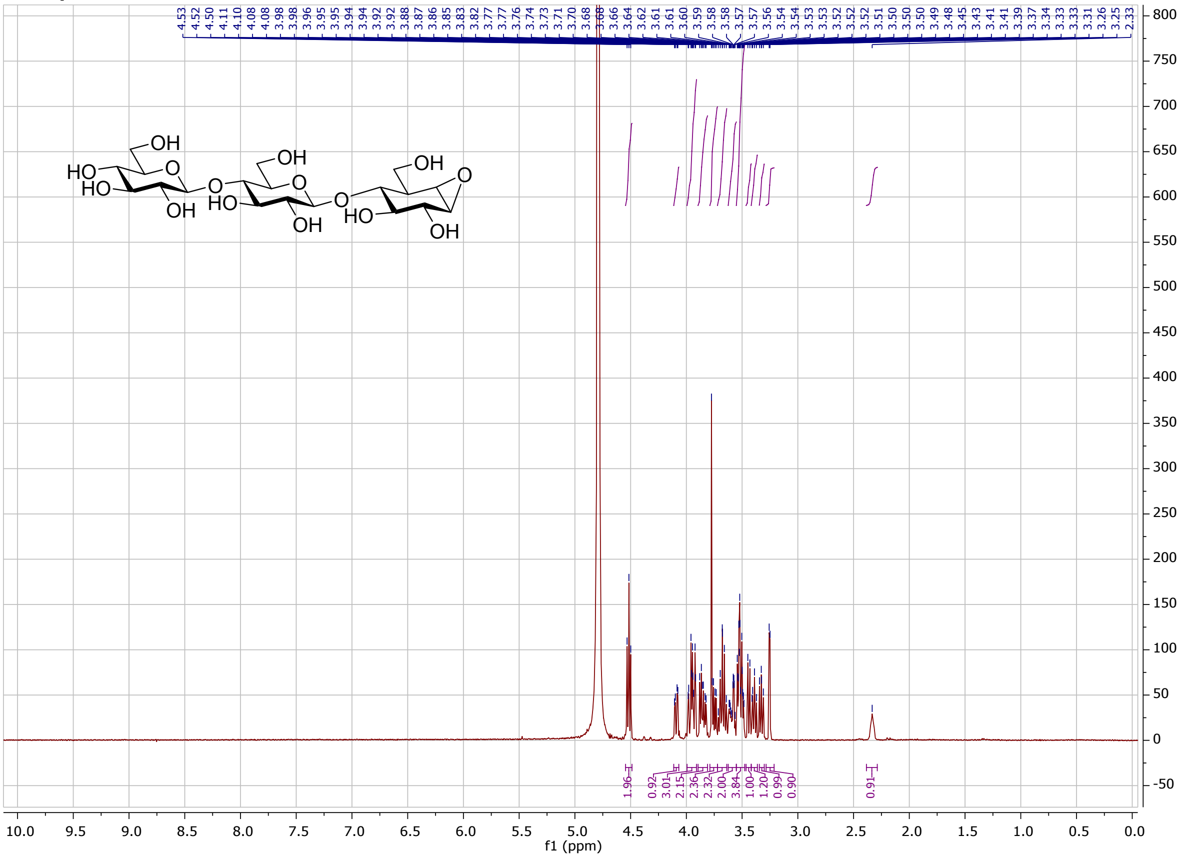

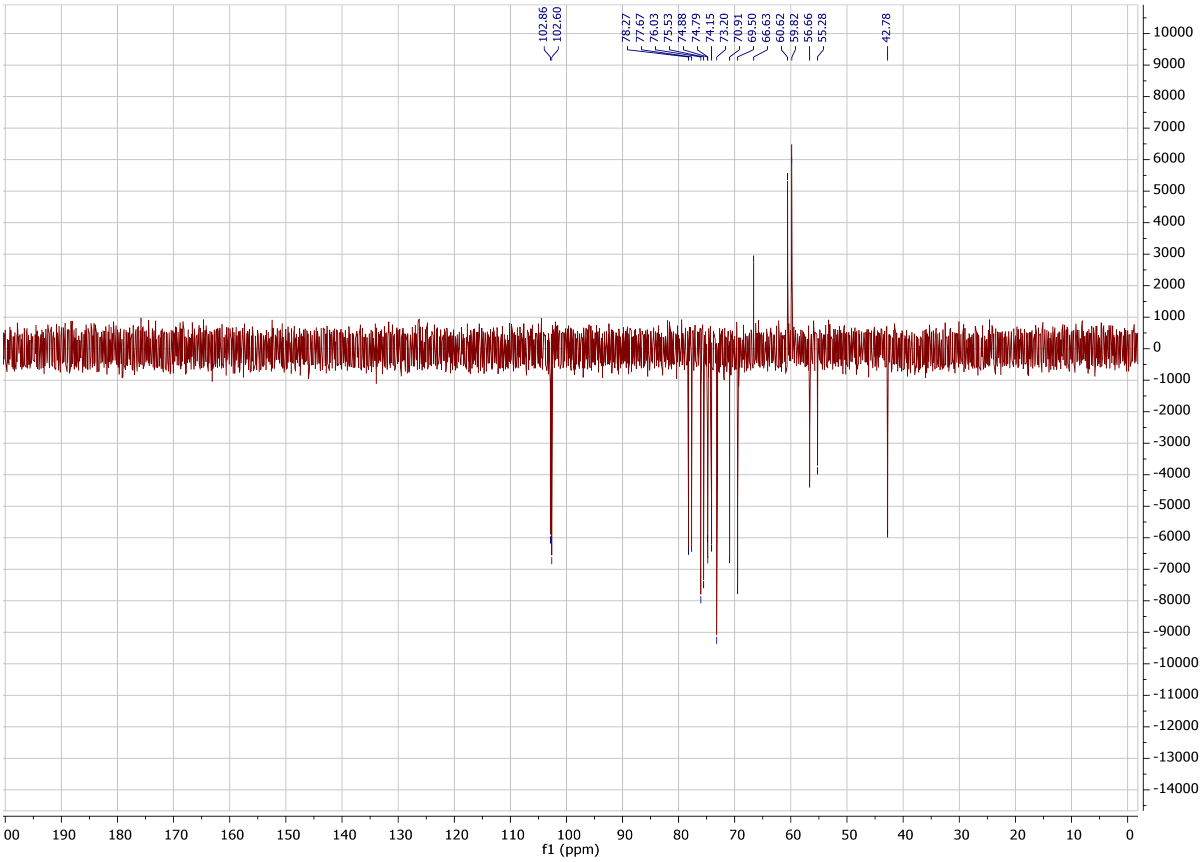

Compound 7 in $CDCl_3$

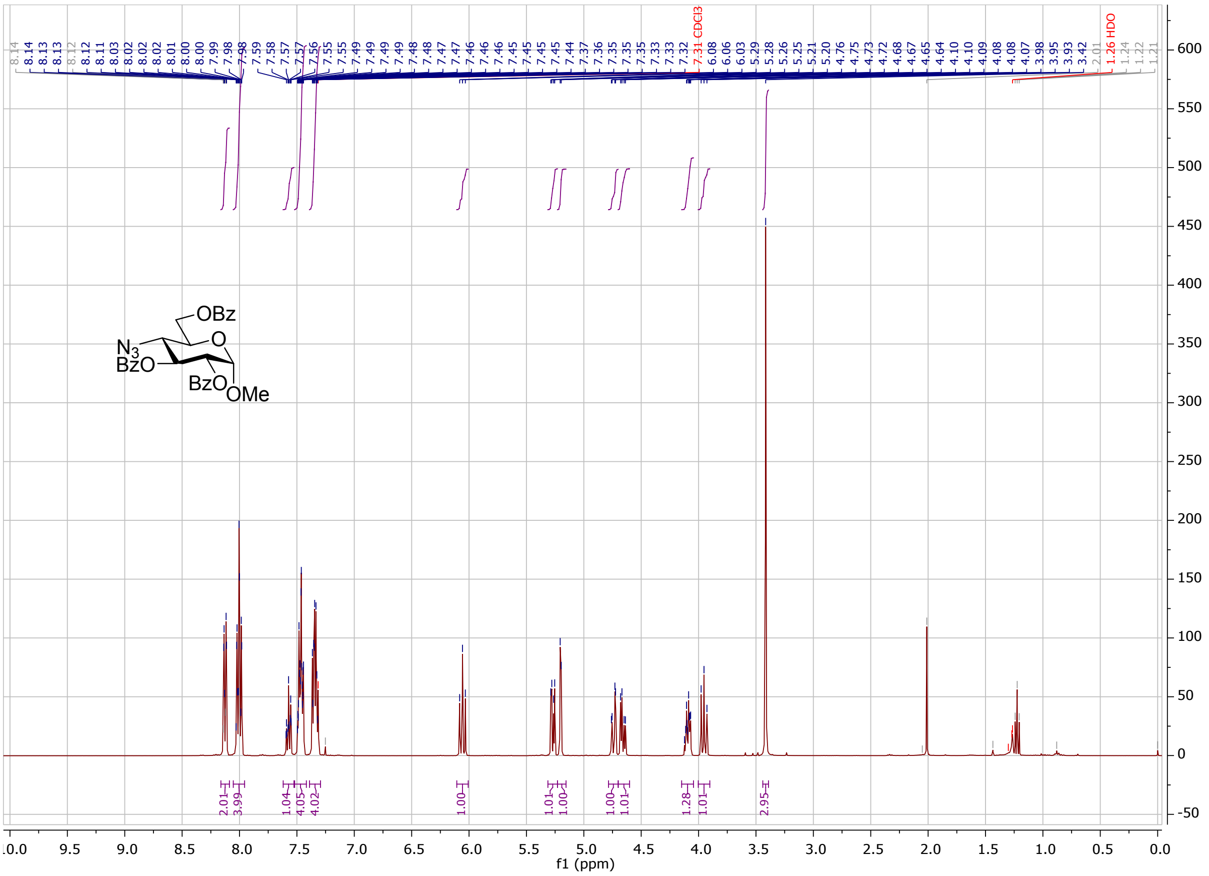

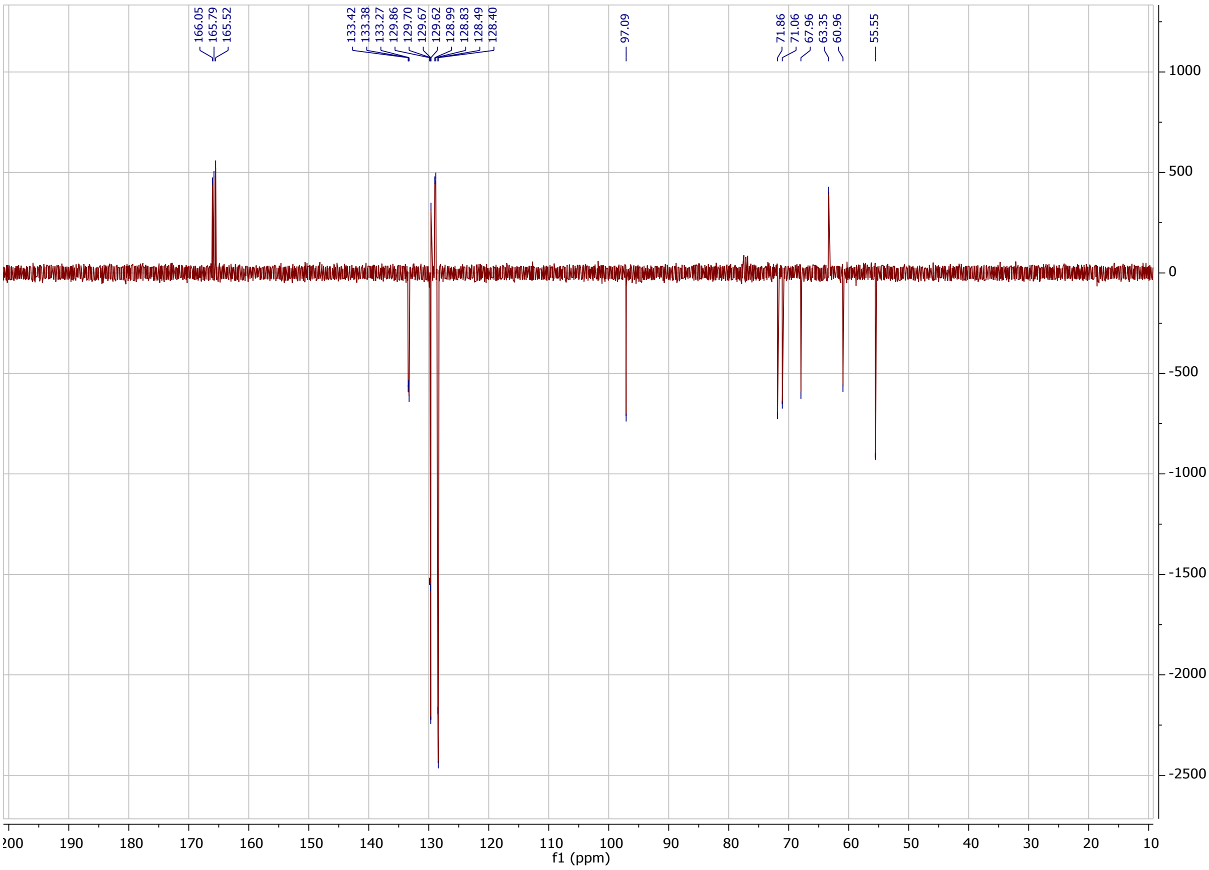

Compound 8 in $CDCl_3$

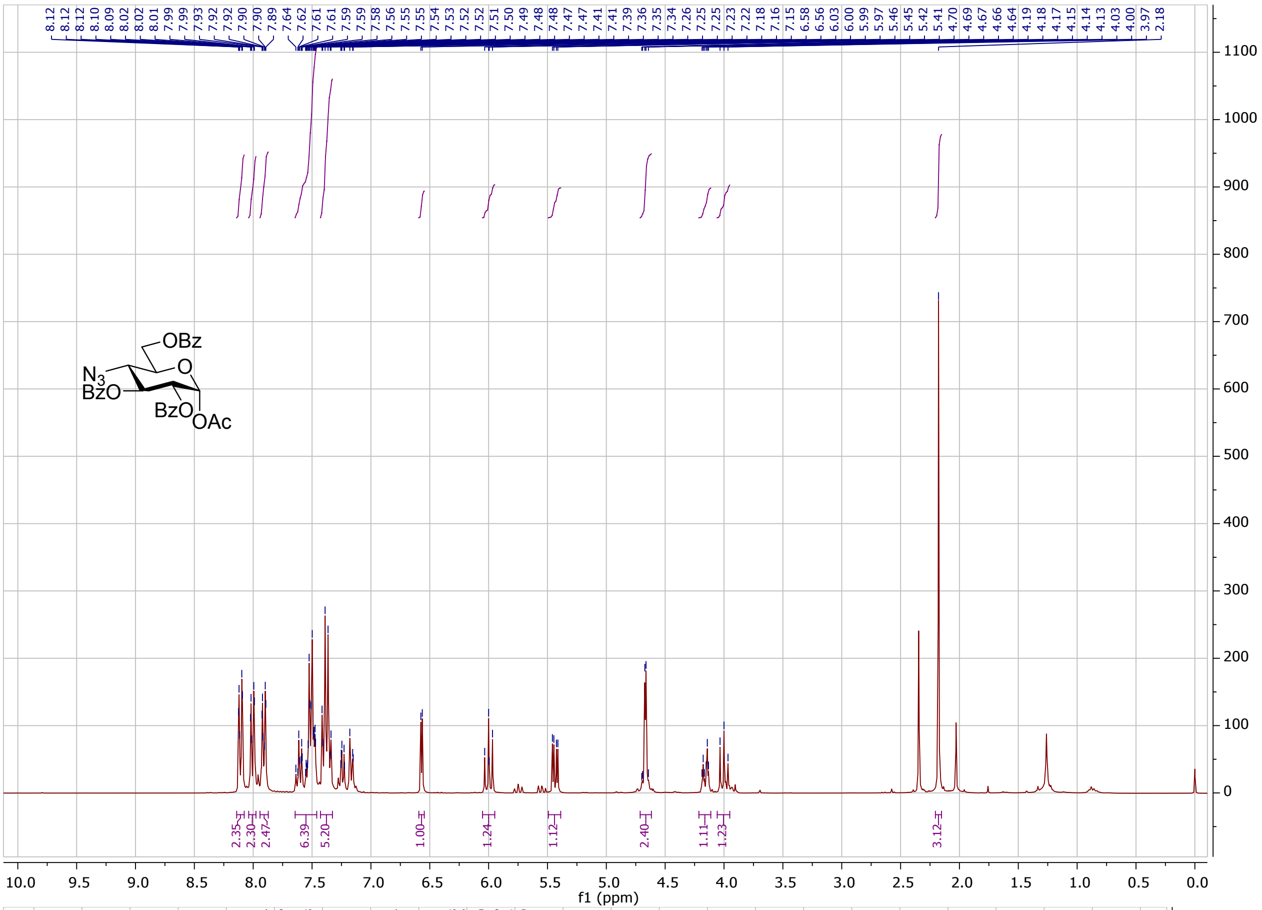

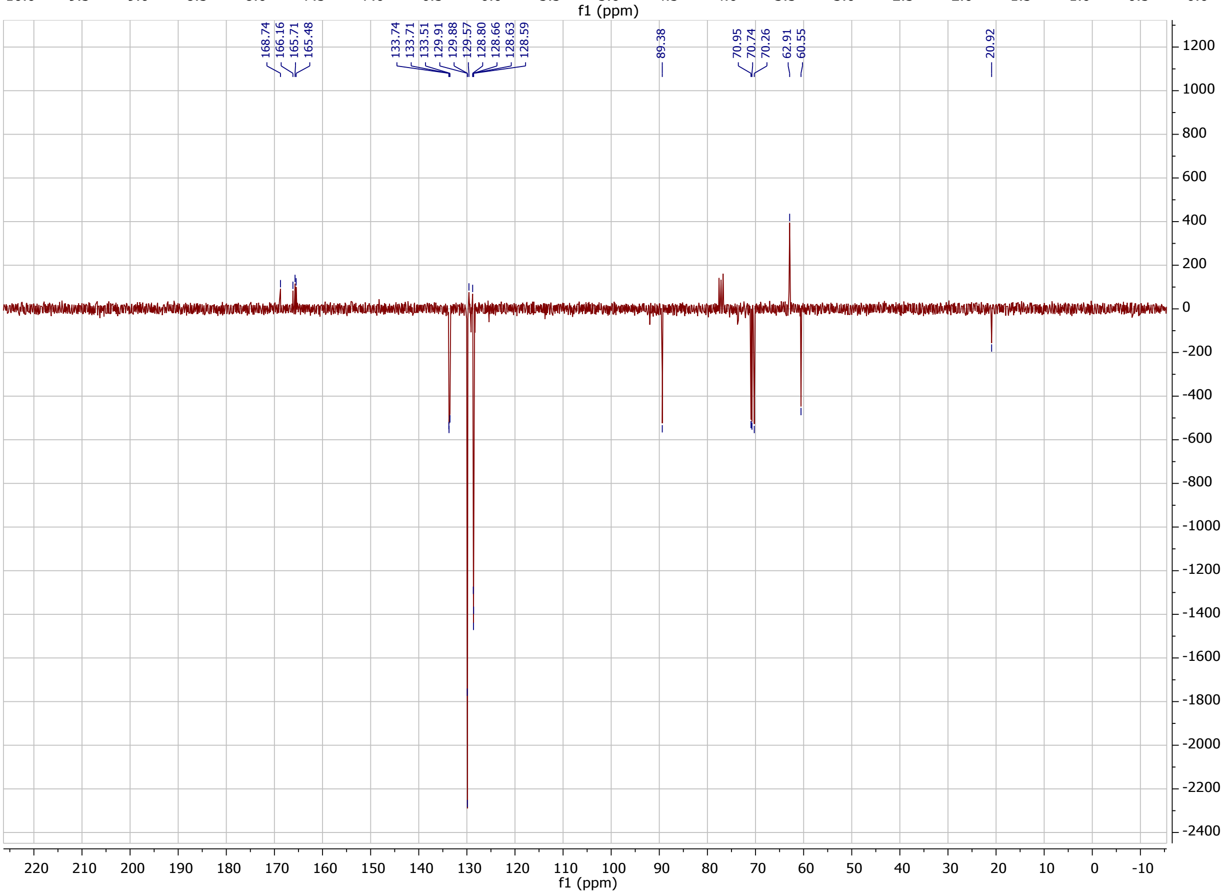

Compound 9 in $CDCl_3$

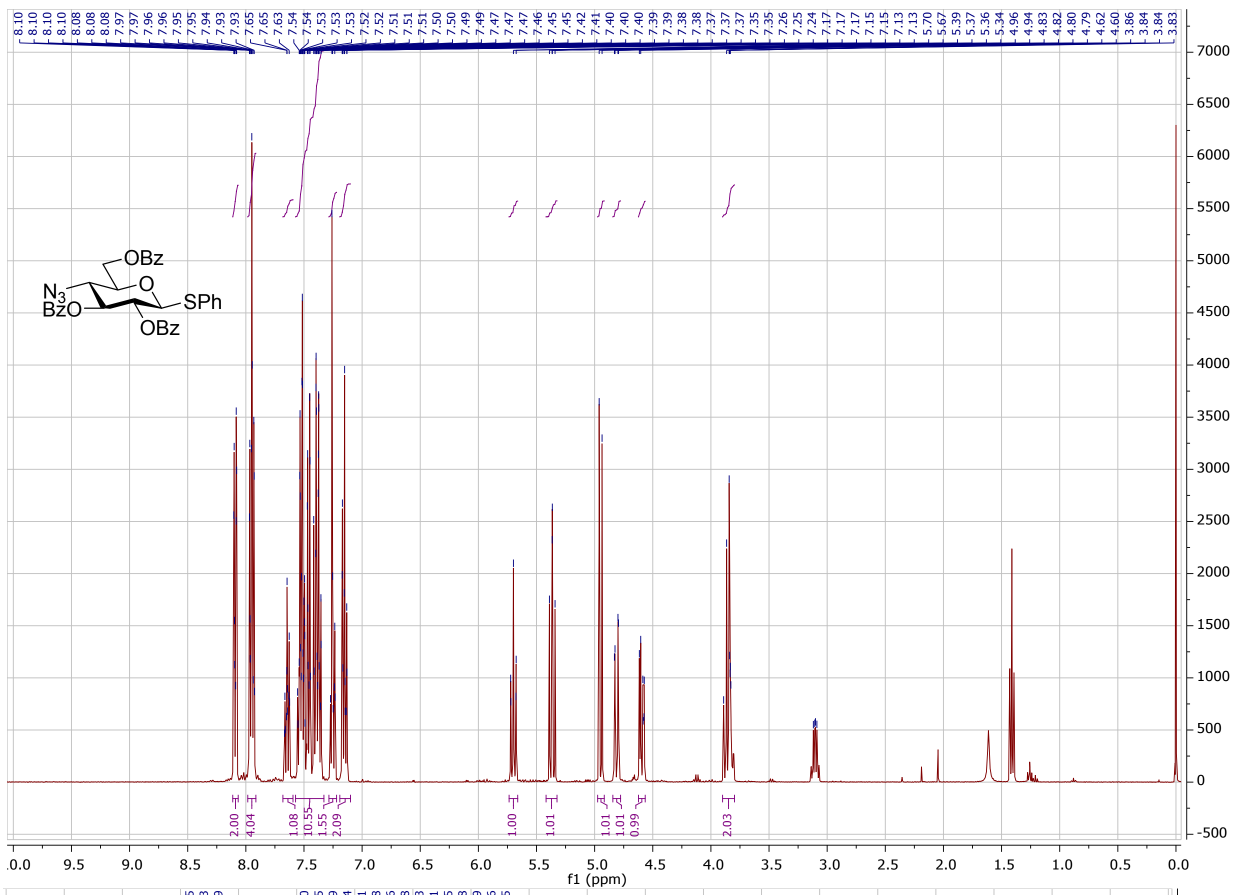

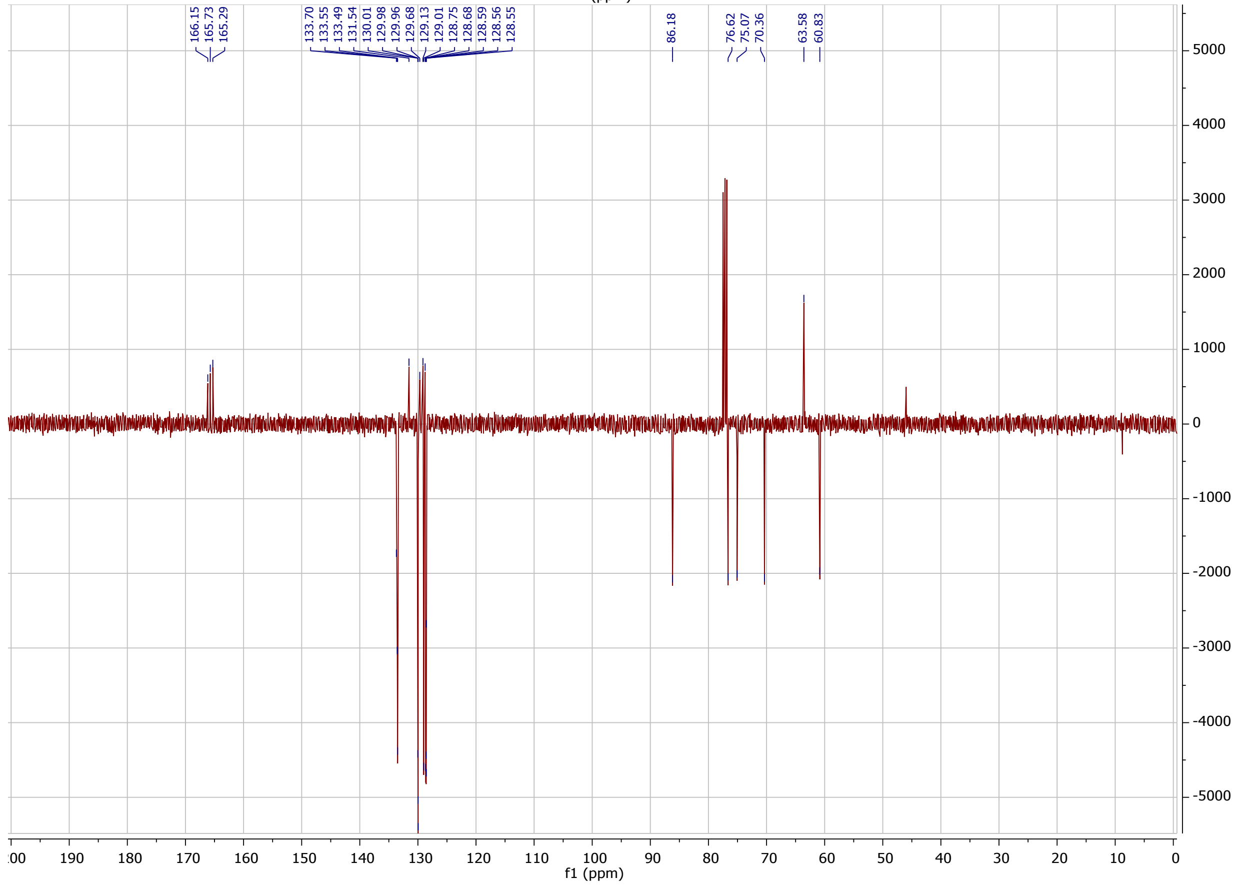

Compound 10 in $CDCl_3$

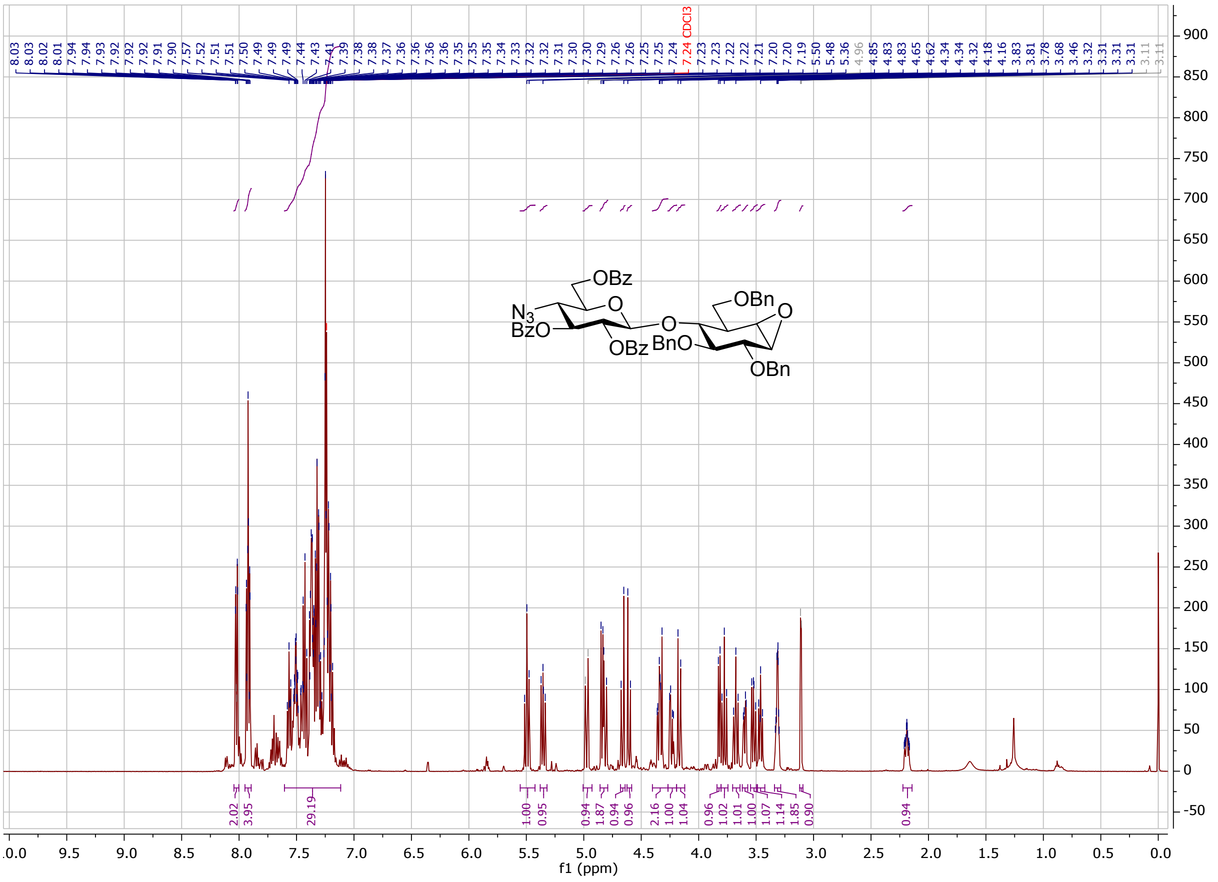

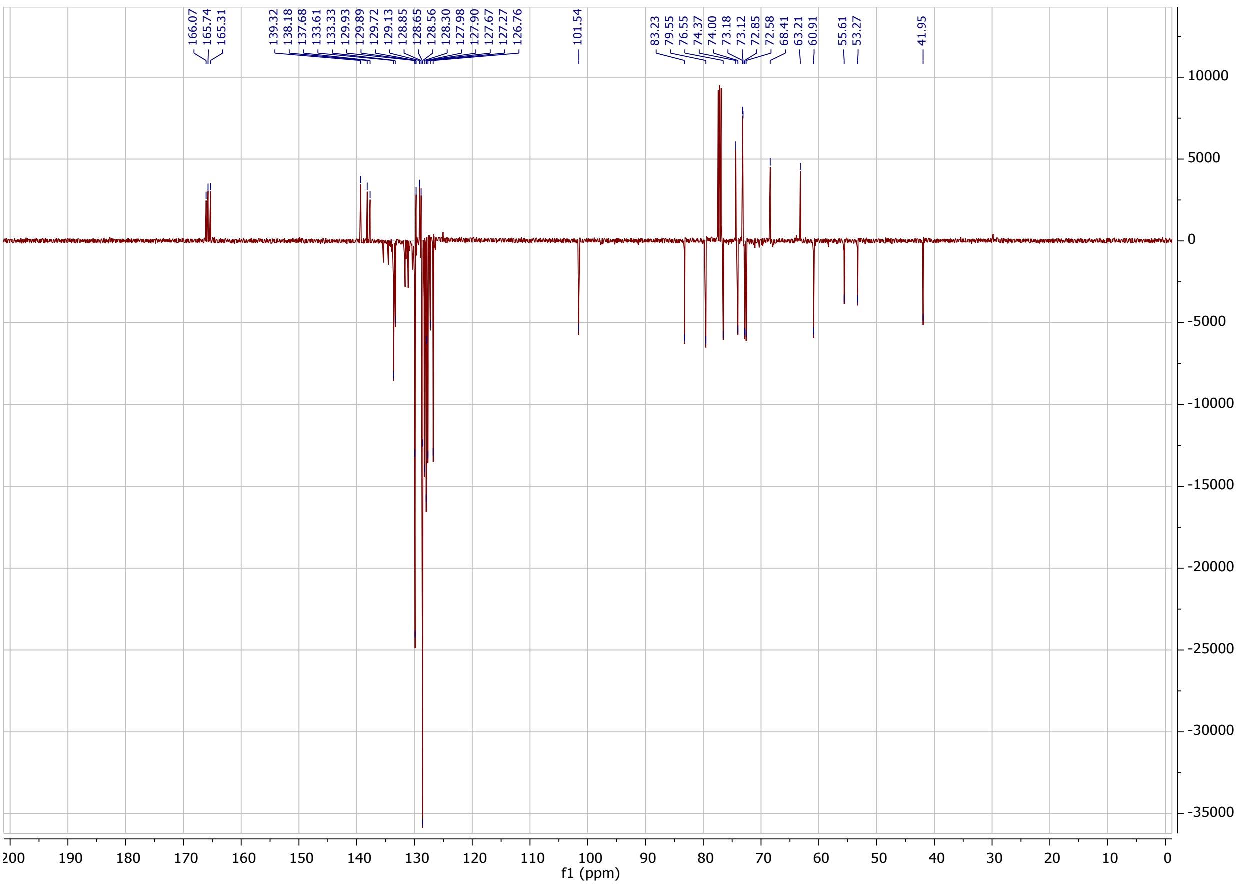

Compound 11 in $CDCl_3$

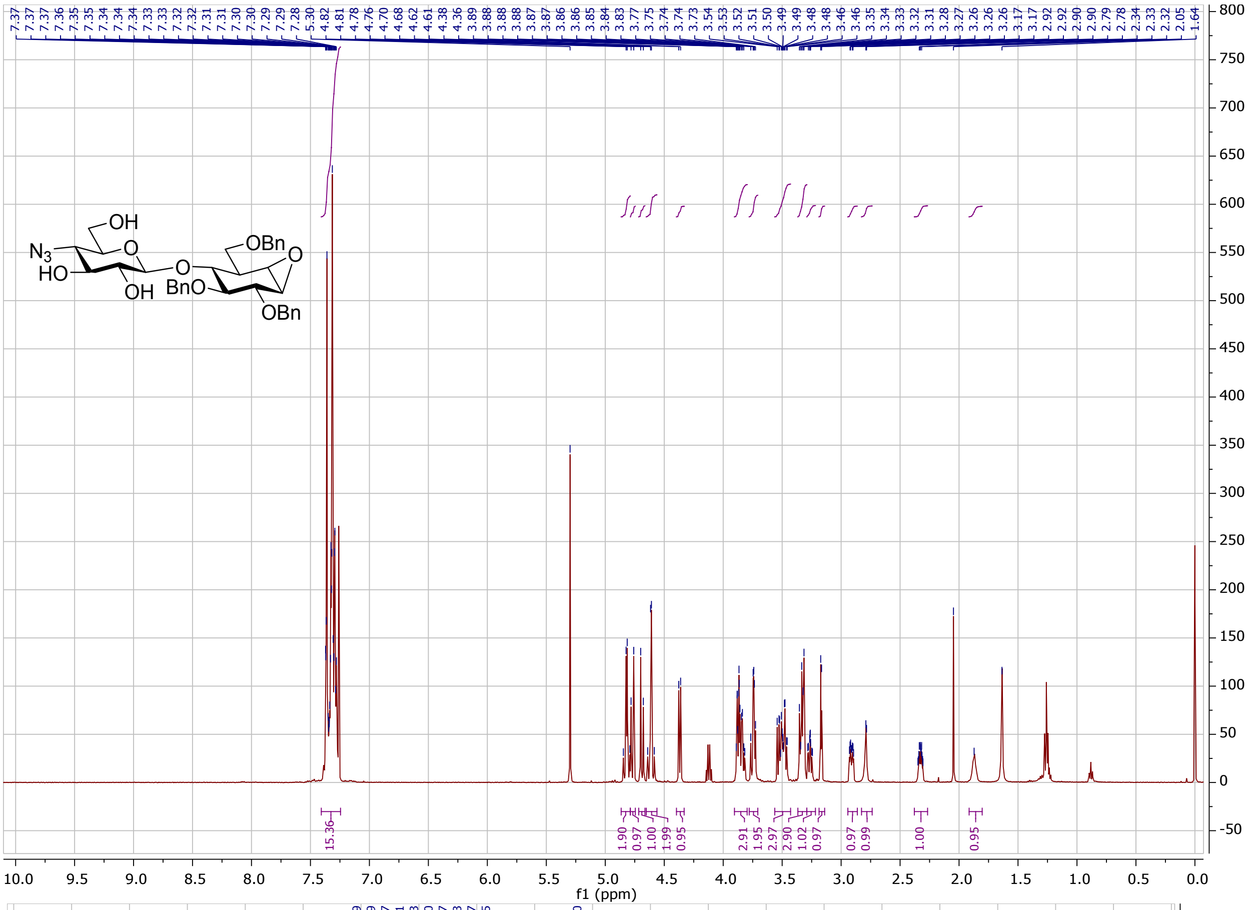

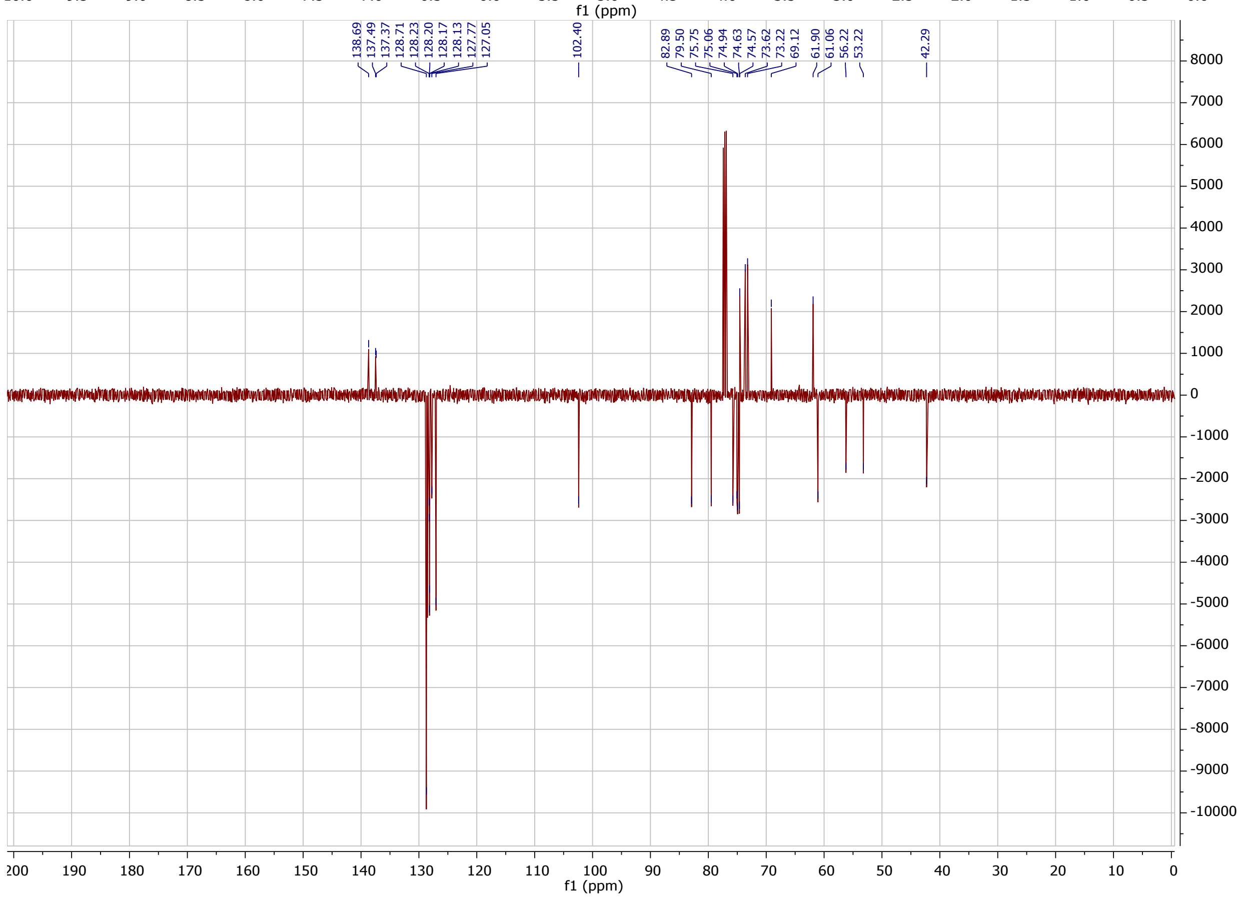

Compound 12 in D_2O

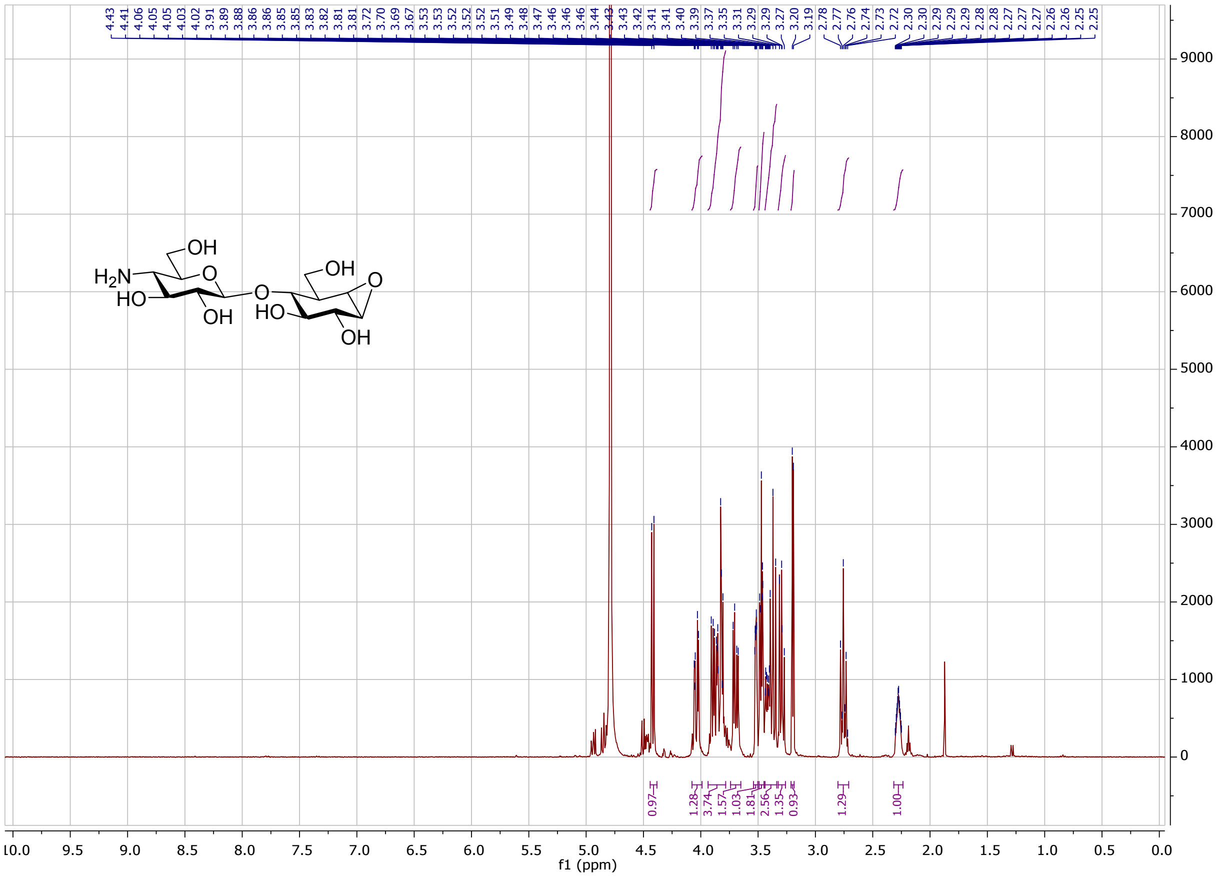
4.43
4.41
4.06
4.05
4.05
4.03
4.02
3.91
3.89
3.88
3.86
3.86
3.85
3.83
3.82
3.81
3.81
3.72
3.70
3.69
3.67
3.53
3.53
3.52
3.52
3.51
3.49
3.48
3.47
3.46
3.46
3.44
3.43
3.43
3.42
3.41
3.41
3.40
3.39
3.37
3.35
3.31
3.29
3.29
3.27
3.20
3.19
2.78
2.77
2.76
2.74
2.73
2.72
2.30
2.30
2.29
2.29
2.29
2.28
2.28
2.27
2.27
2.27
2.26
2.25
2.25
OH
H_2N
O
HO
OH
O
OH
HO
O
OH
0.97
1.28
3.74
1.57
1.03
1.81
2.56
1.35
0.93
1.29
1.00
f1 (ppm)

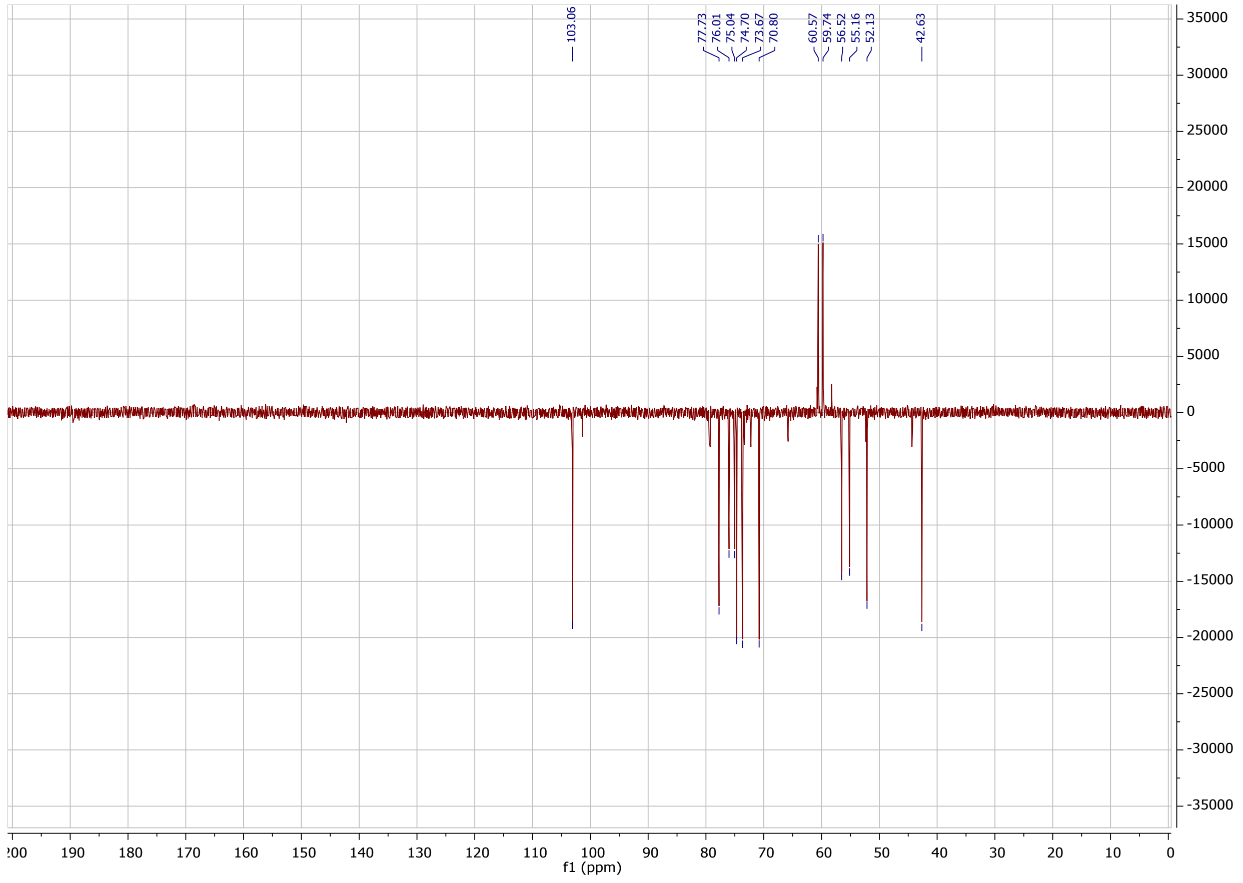
103.06
77.73
76.01
75.04
74.70
73.67
70.80
60.57
59.74
56.52
55.16
52.13
42.63
f1 (ppm)

Compound 13 in D_2O

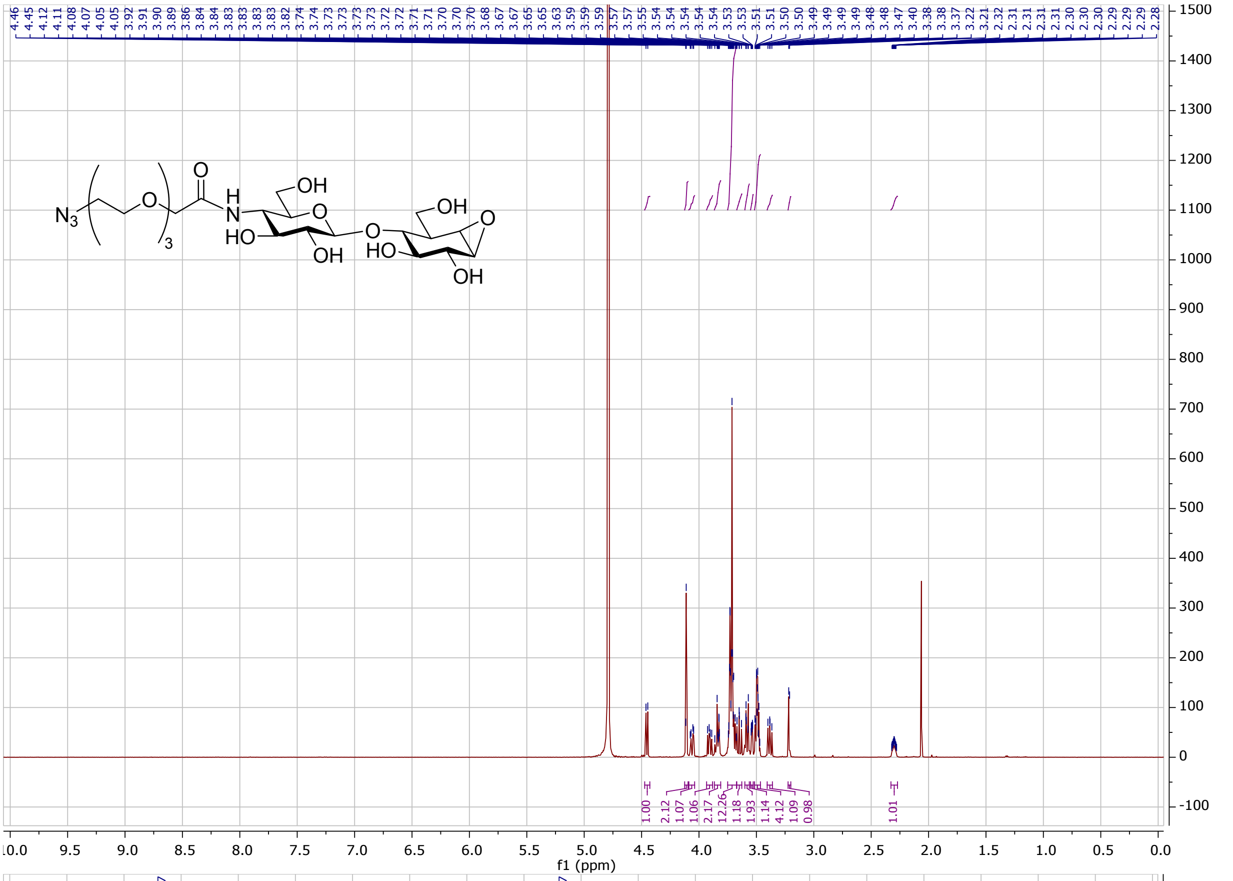

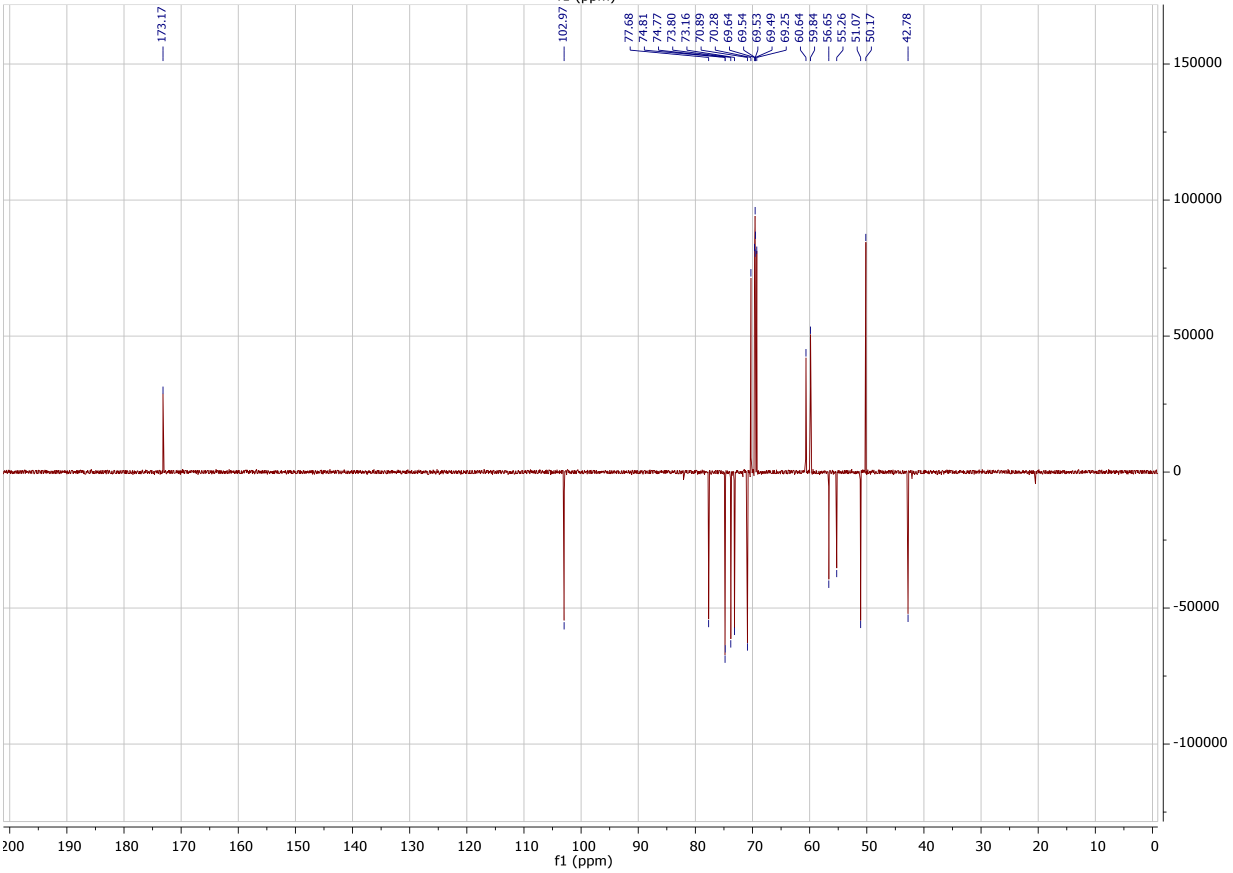

Compound 14 in MeOD

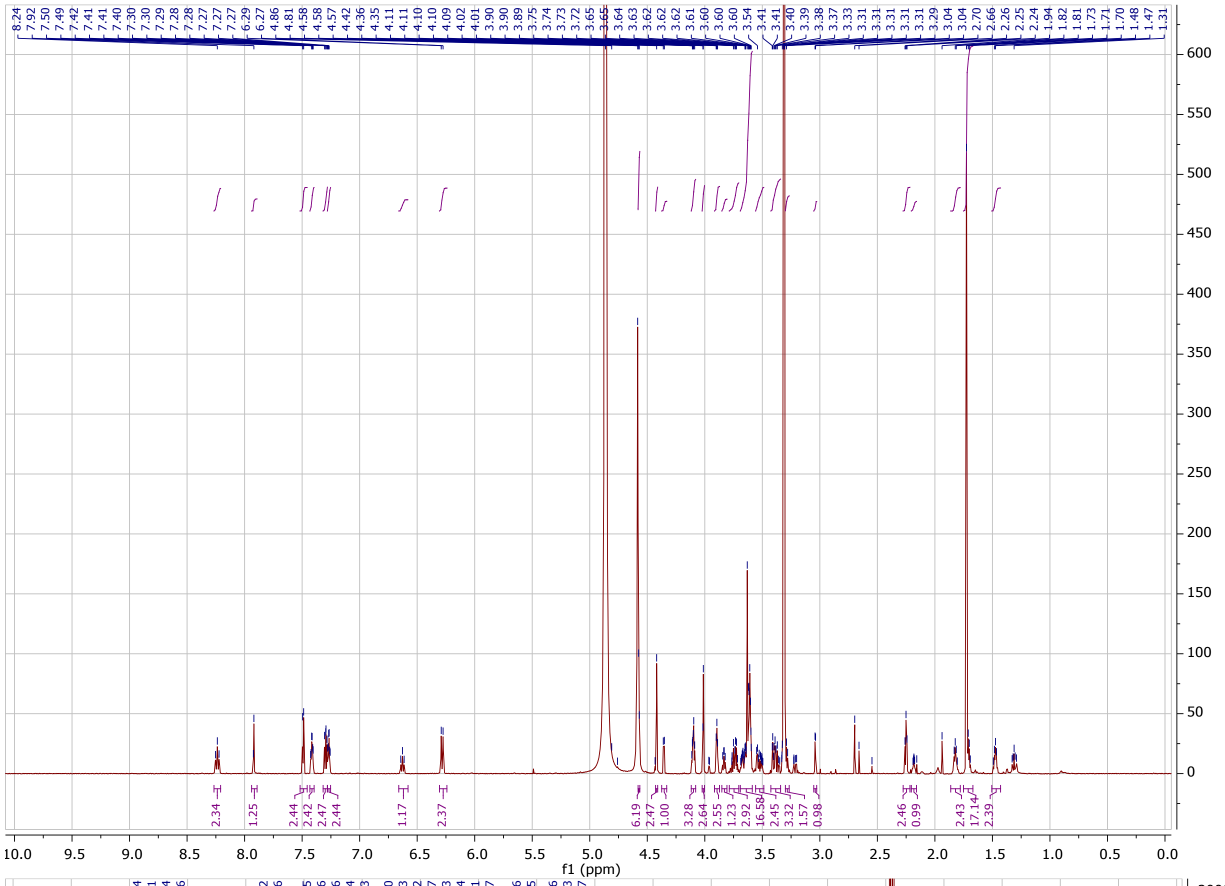
8.24 7.92 7.50 7.49 7.42 7.41 7.40 7.30 7.30 7.29 7.28 7.27 7.27 7.27 6.29 6.27 4.86 4.81 4.58 4.58 4.57 4.42 4.36 4.35 4.11 4.11 4.10 4.10 4.09 4.02 4.01 3.90 3.90 3.89 3.75 3.74 3.73 3.72 3.65 3.65 3.64 3.63 3.62 3.62 3.62 3.61 3.60 3.60 3.60 3.54 3.41 3.41 3.40 3.39 3.38 3.37 3.33 3.31 3.31 3.31 3.31 3.31 3.29 3.04 3.04 2.70 2.66 2.26 2.25 2.24 1.94 1.82 1.81 1.73 1.71 1.70 1.48 1.47 1.31
2.34 1.25 2.44 2.42 2.47 2.44 1.17 2.37 6.19 2.47 1.00 3.28 2.64 2.55 1.23 2.92 16.58 2.45 3.32 1.57 0.98 2.46 0.99 2.43 17.14 2.39
f1 (ppm)

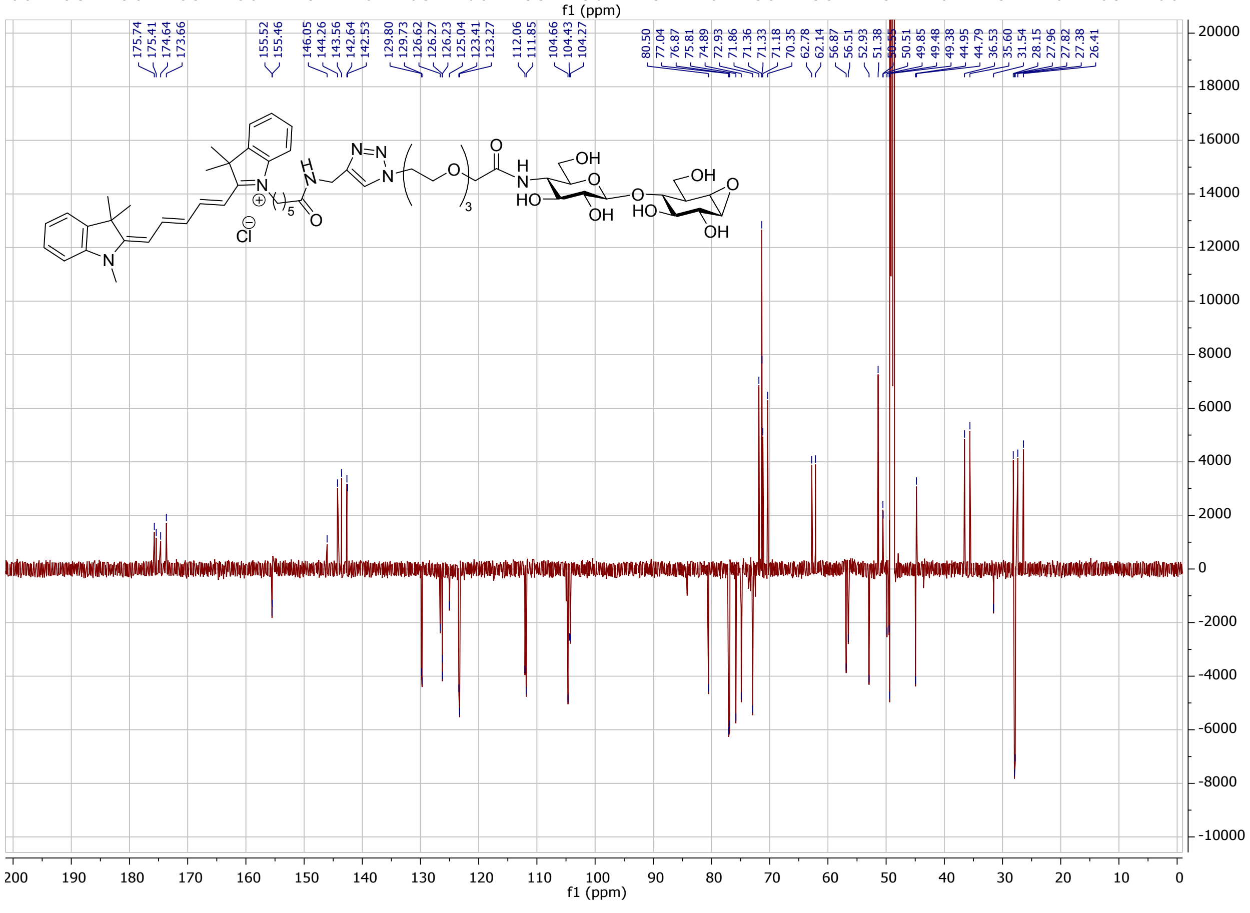
175.74 175.41 174.64 173.66 155.52 155.46 146.05 144.26 143.56 142.64 142.53 129.80 129.73 126.62 126.27 126.23 125.04 123.41 123.27 112.06 111.85 104.66 104.43 104.27 80.50 77.04 76.87 75.81 74.89 72.93 71.86 71.36 71.33 71.18 70.35 62.78 62.14 56.87 56.51 52.93 51.38 50.55 50.51 49.85 49.48 49.38 44.95 44.79 36.53 35.60 31.54 28.15 27.96 27.82 27.38 26.41
f1 (ppm)
Cl
OH
HO
OH
HO
OH
O

Compound 15 in MeOD

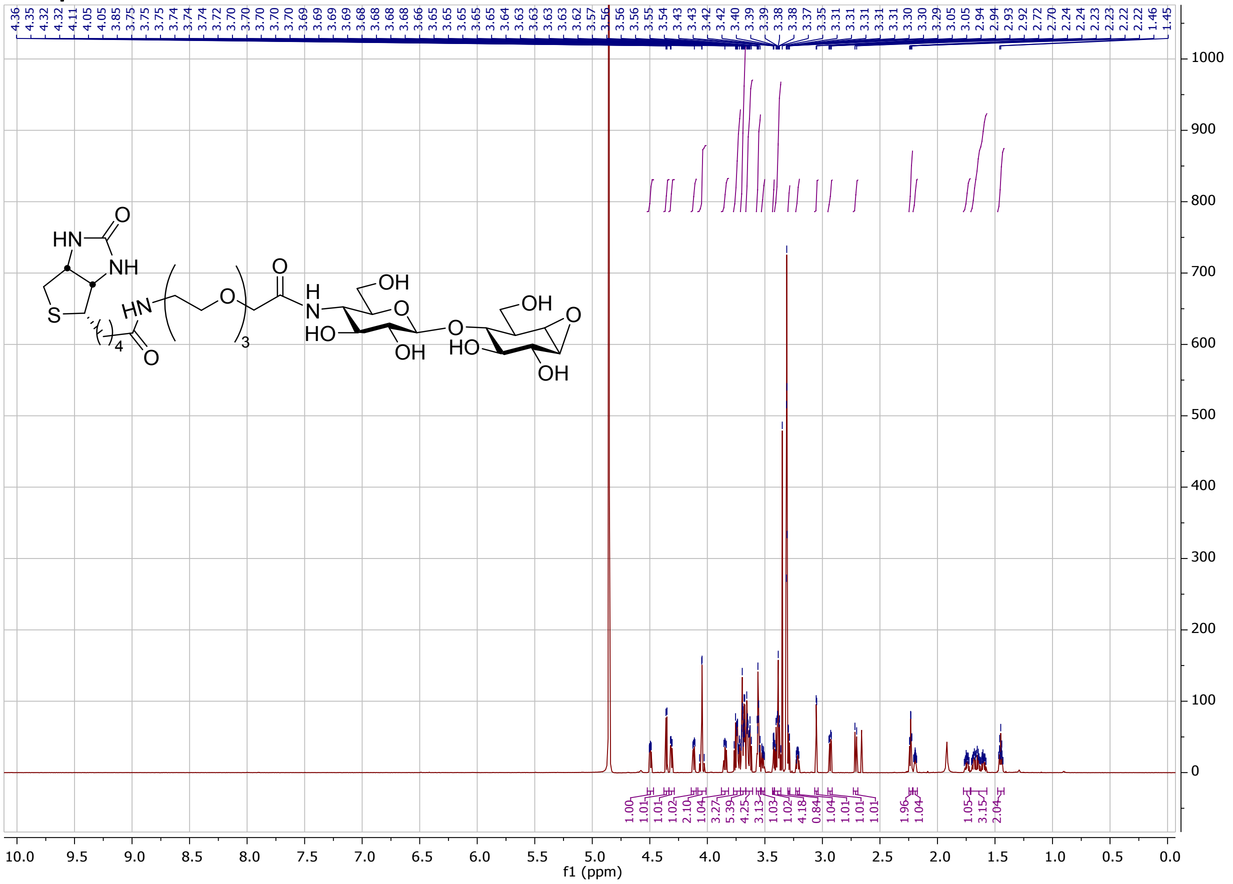

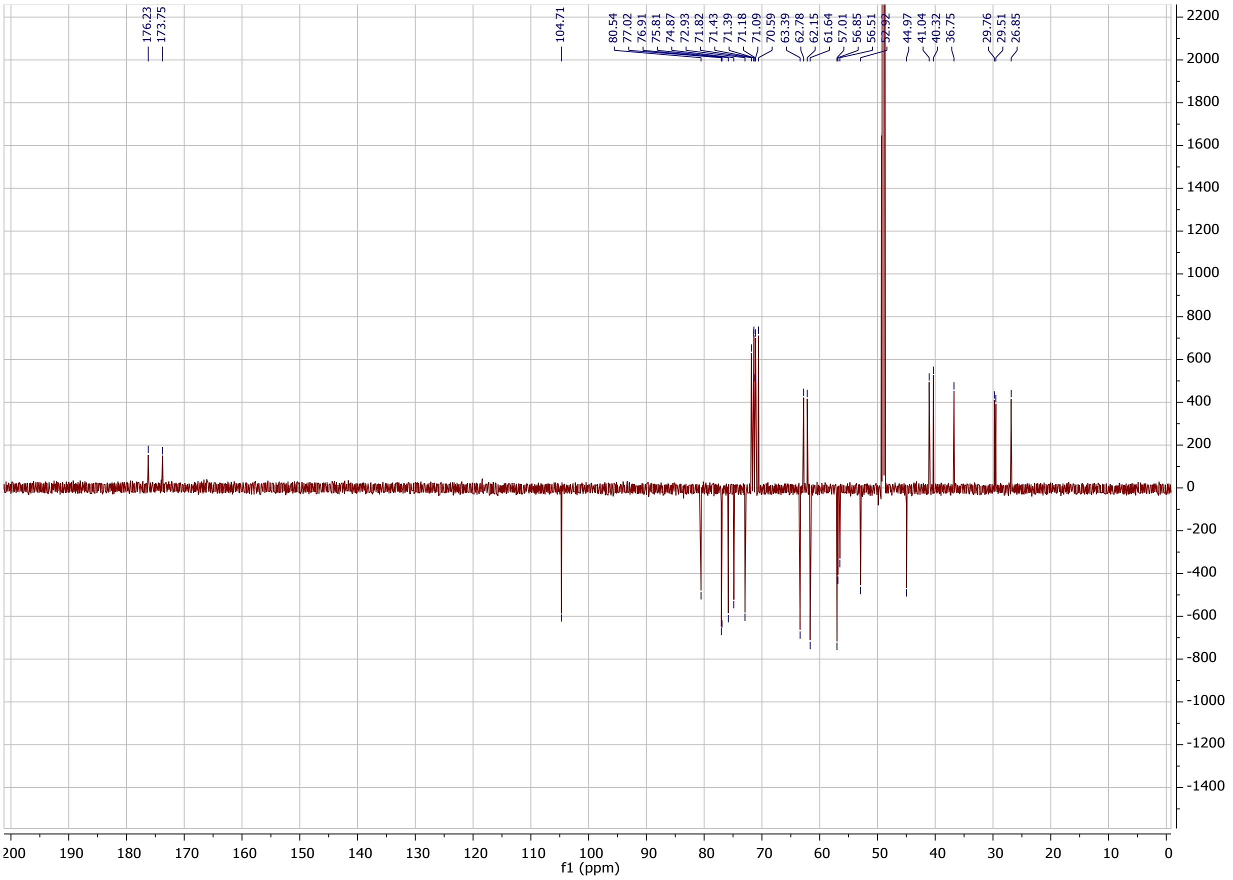

Compound S1 in CD_3CN

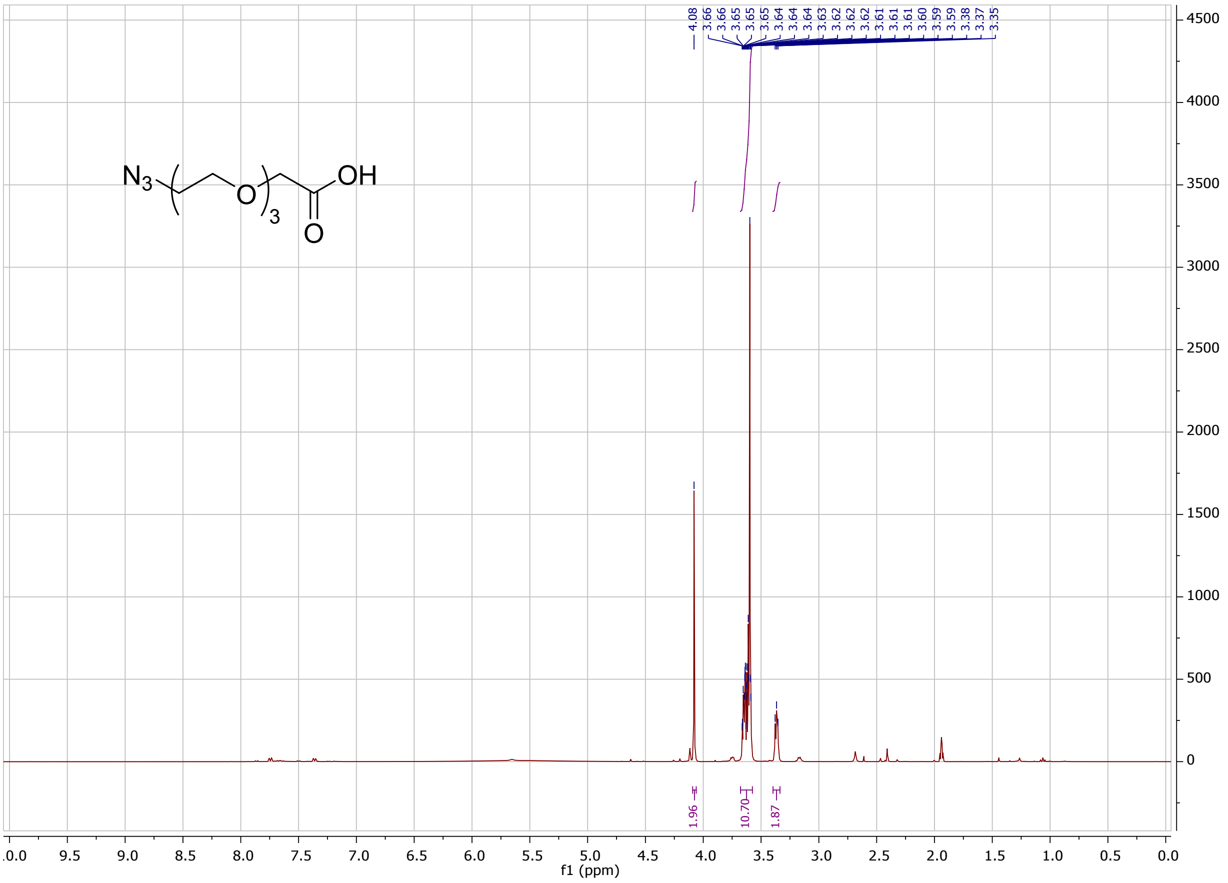

4.08
3.66 3.65 3.65 3.65 3.65 3.64 3.64 3.64 3.63 3.62 3.62 3.62 3.61 3.61 3.61 3.60 3.59 3.59 3.38 3.37 3.35
N3 O 3 OH O
1.96
10.70
1.87
f1 (ppm)

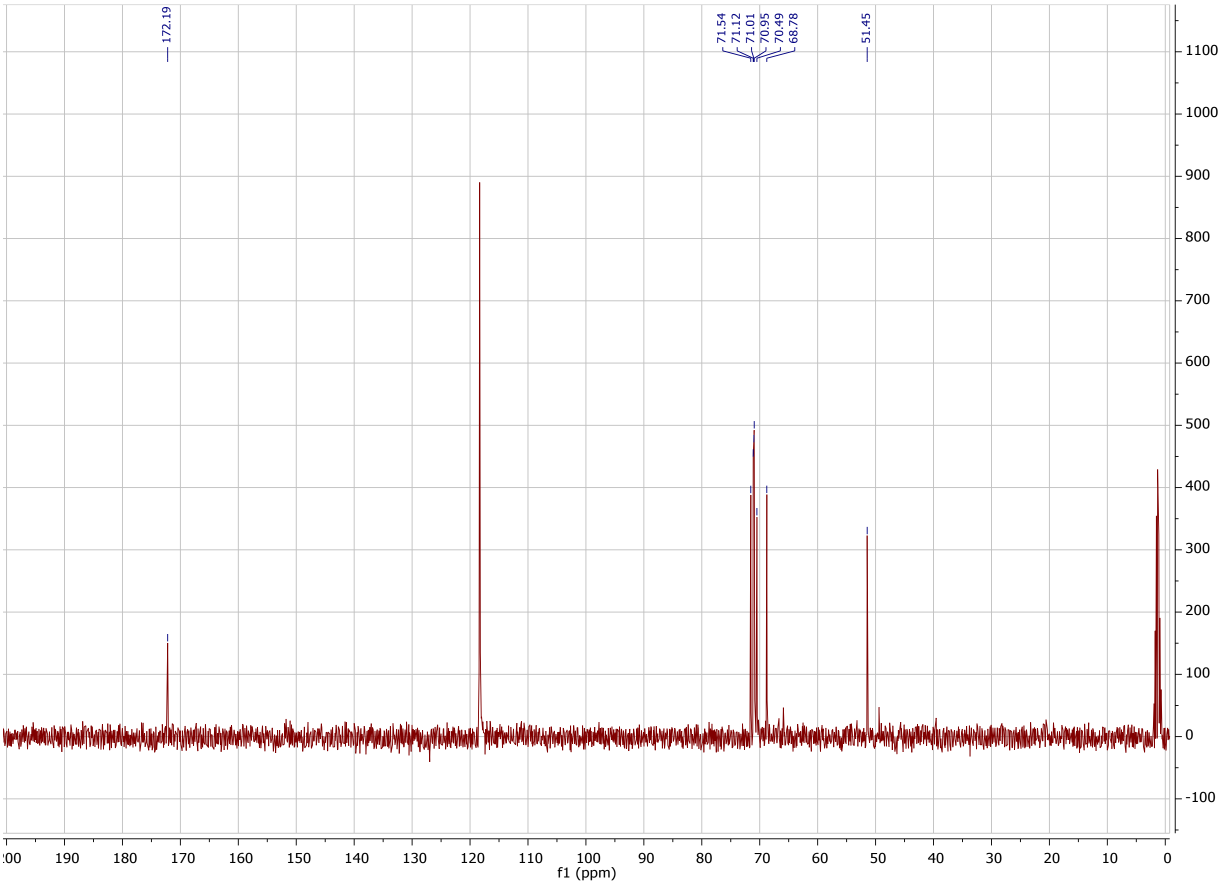

172.19
71.54 71.12 71.01 70.95 70.49 68.78
51.45
f1 (ppm)

Compound 17 in $CDCl_3$

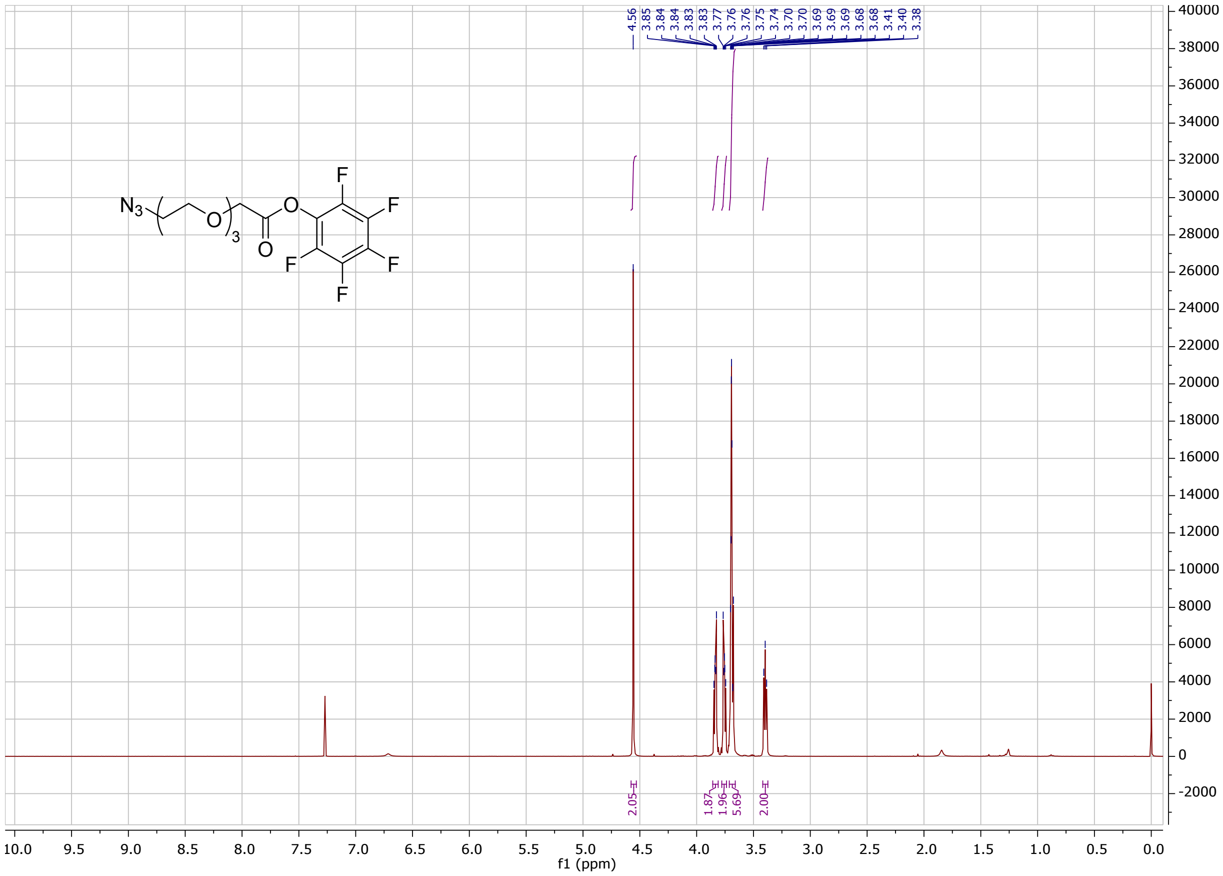
4.56
3.85
3.84
3.84
3.83
3.83
3.77
3.76
3.76
3.75
3.74
3.70
3.70
3.69
3.69
3.69
3.68
3.41
3.40
3.38
2.05
1.87
1.96
5.69
2.00
f1 (ppm)

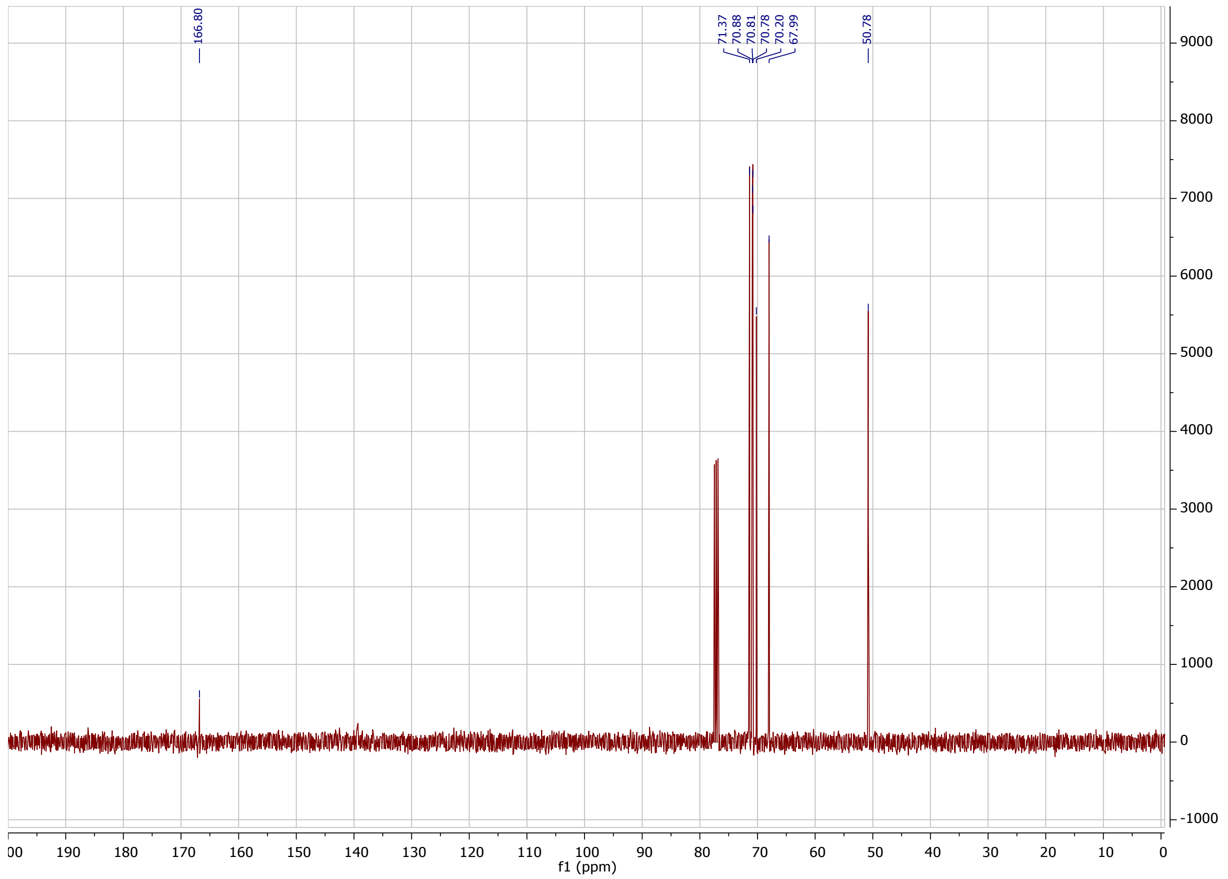
166.80
71.37
70.88
70.81
70.78
70.20
67.99
50.78
f1 (ppm)

Compound S2 in $CDCl_3$

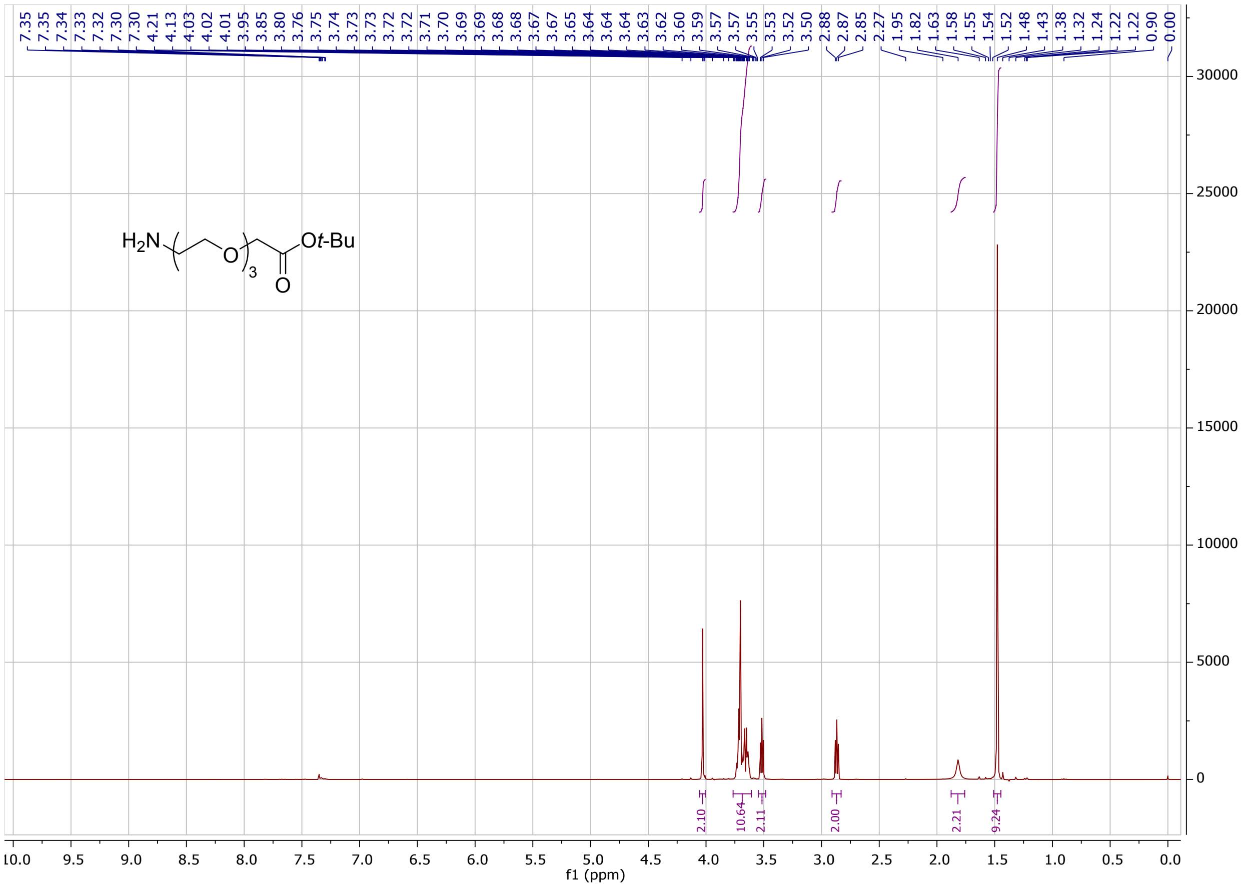

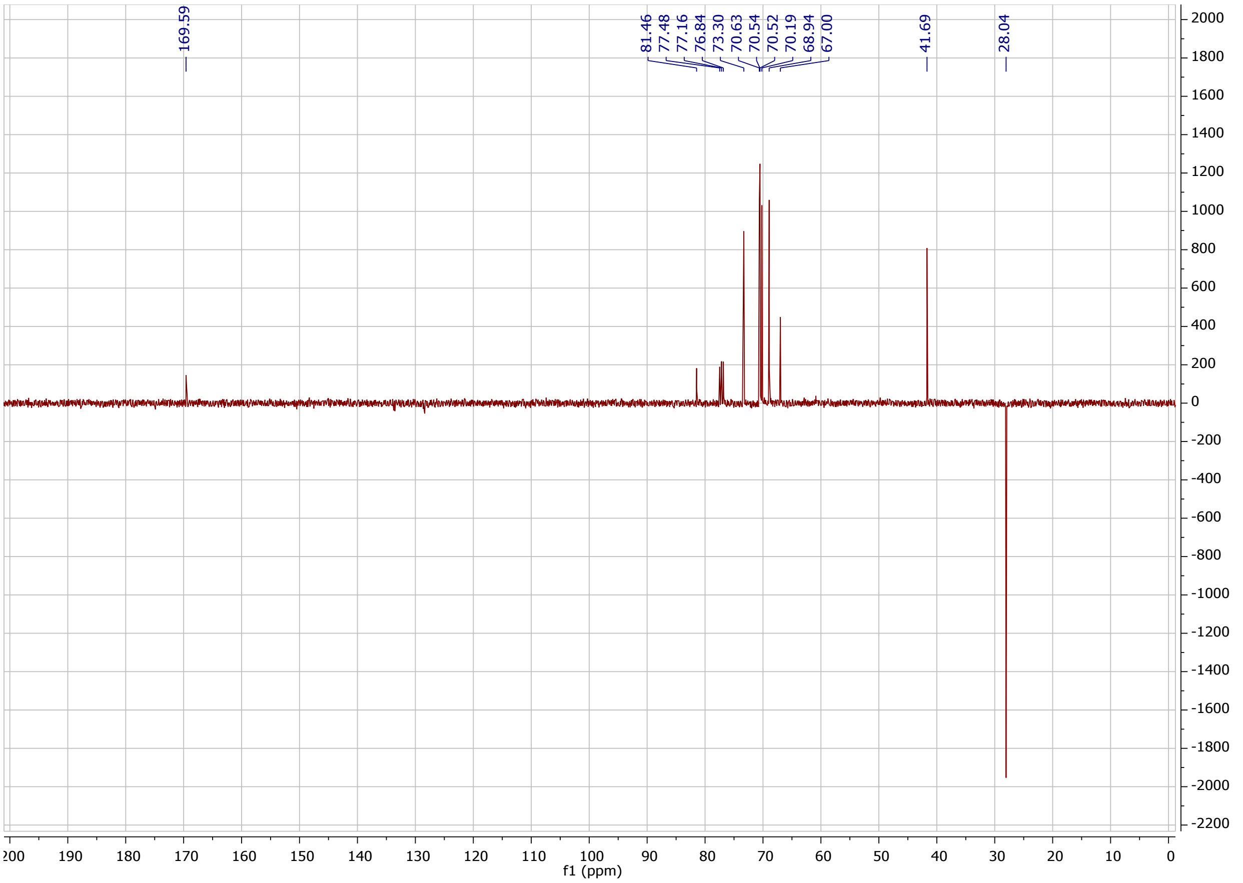

Compound S3 in $CDCl_3$

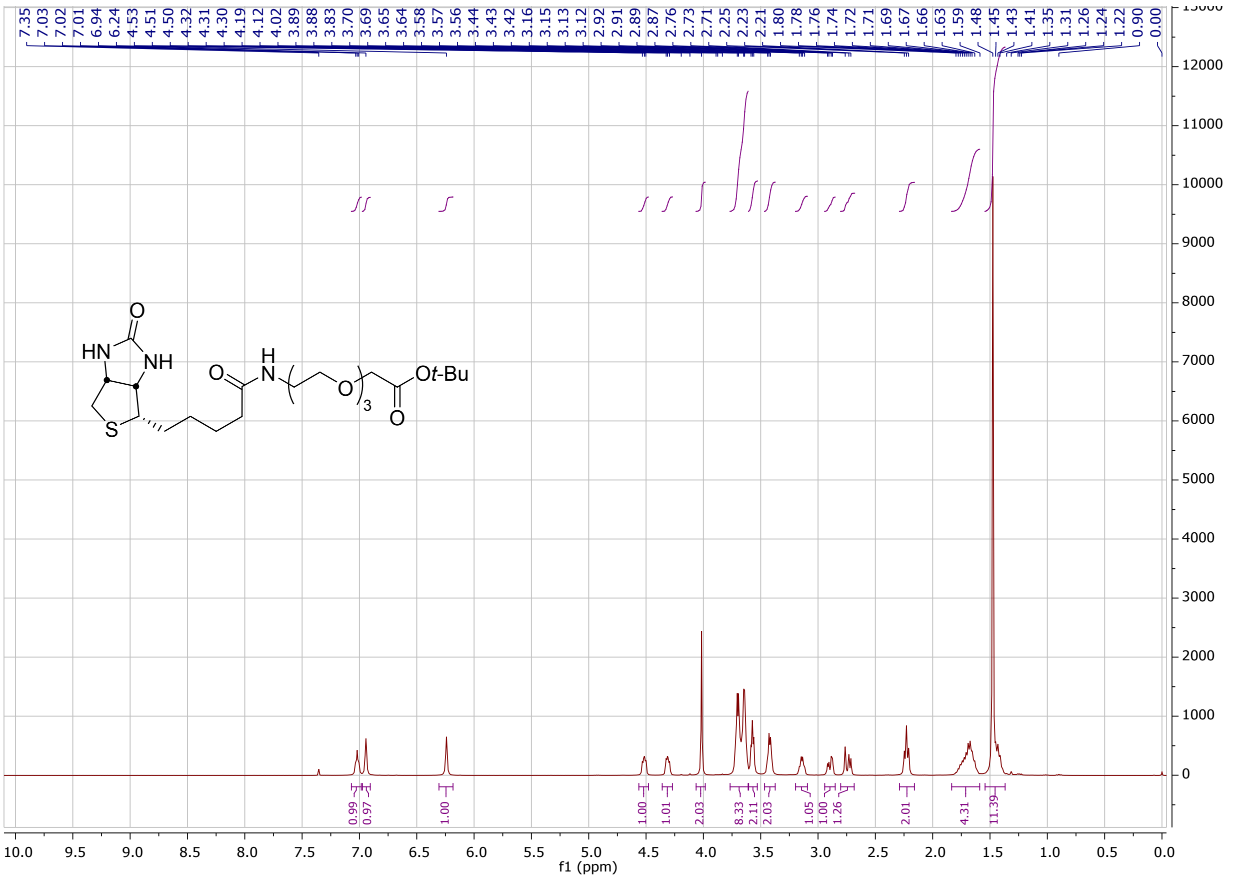

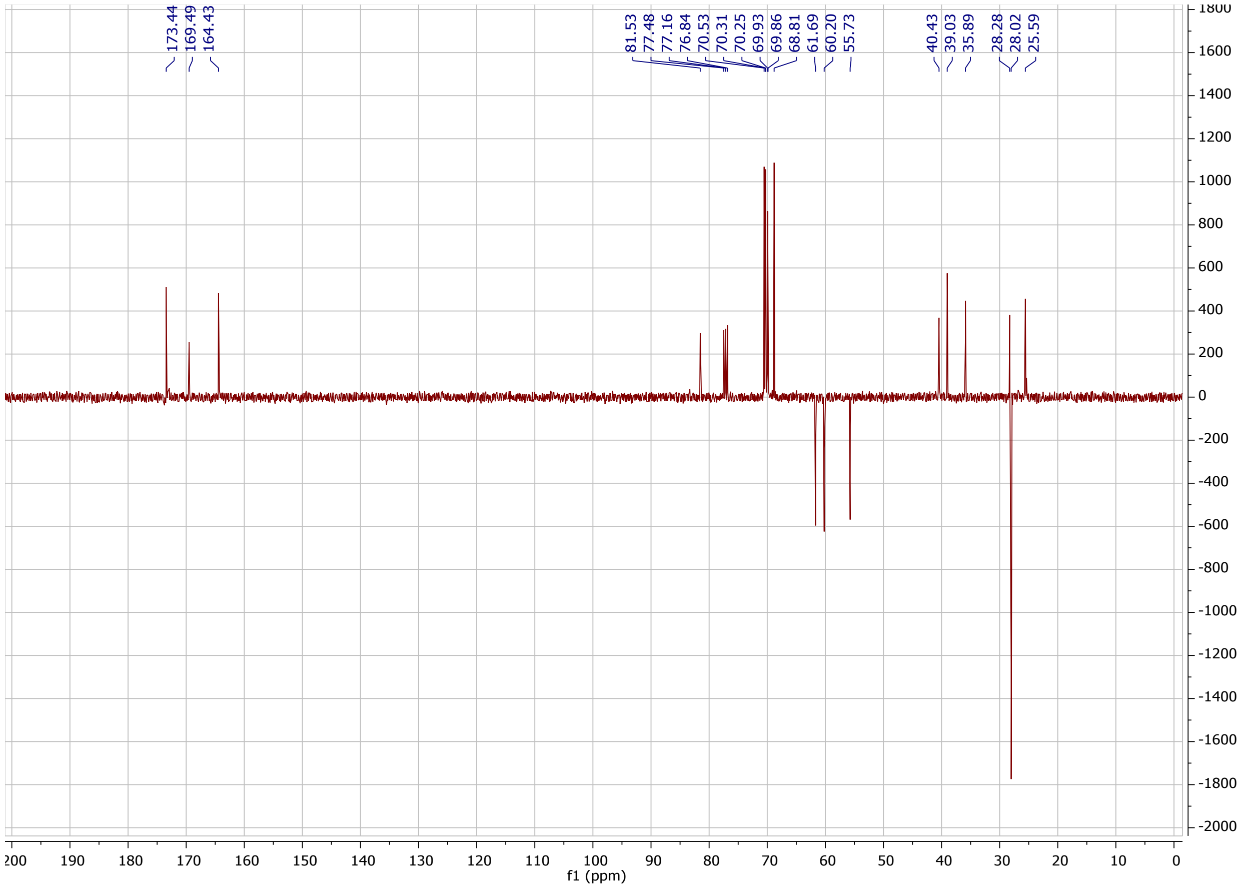

Compound 18 in $CDCl_3$/MeOD

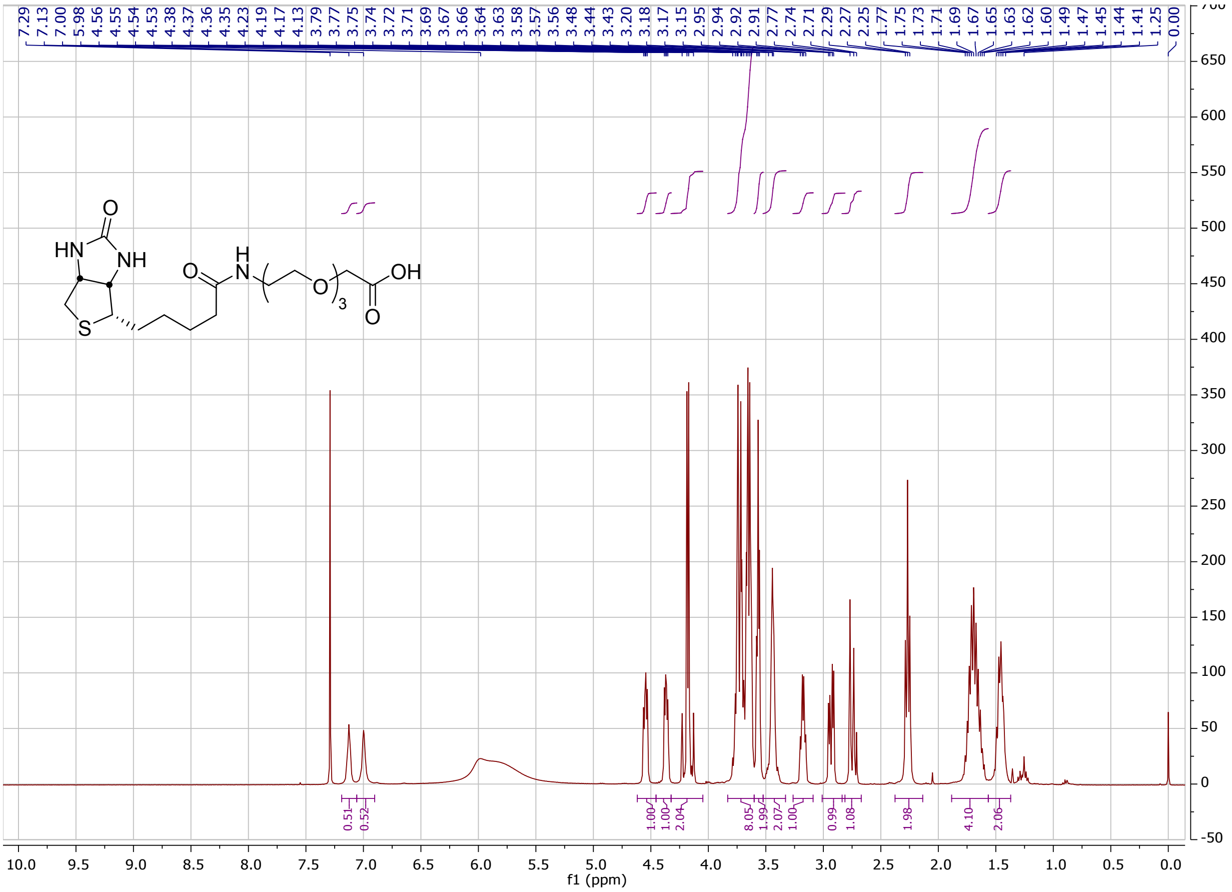

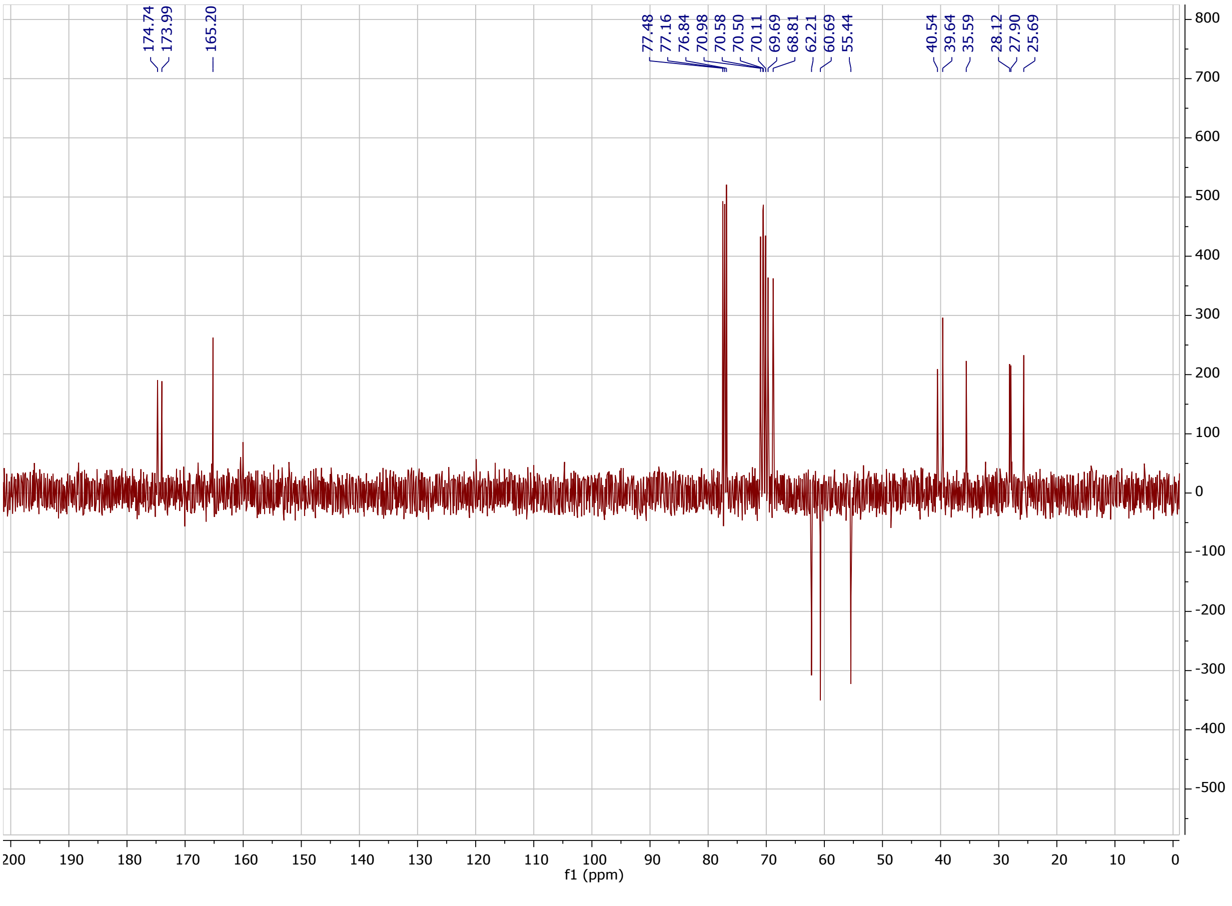

Compound 19 in MeOD

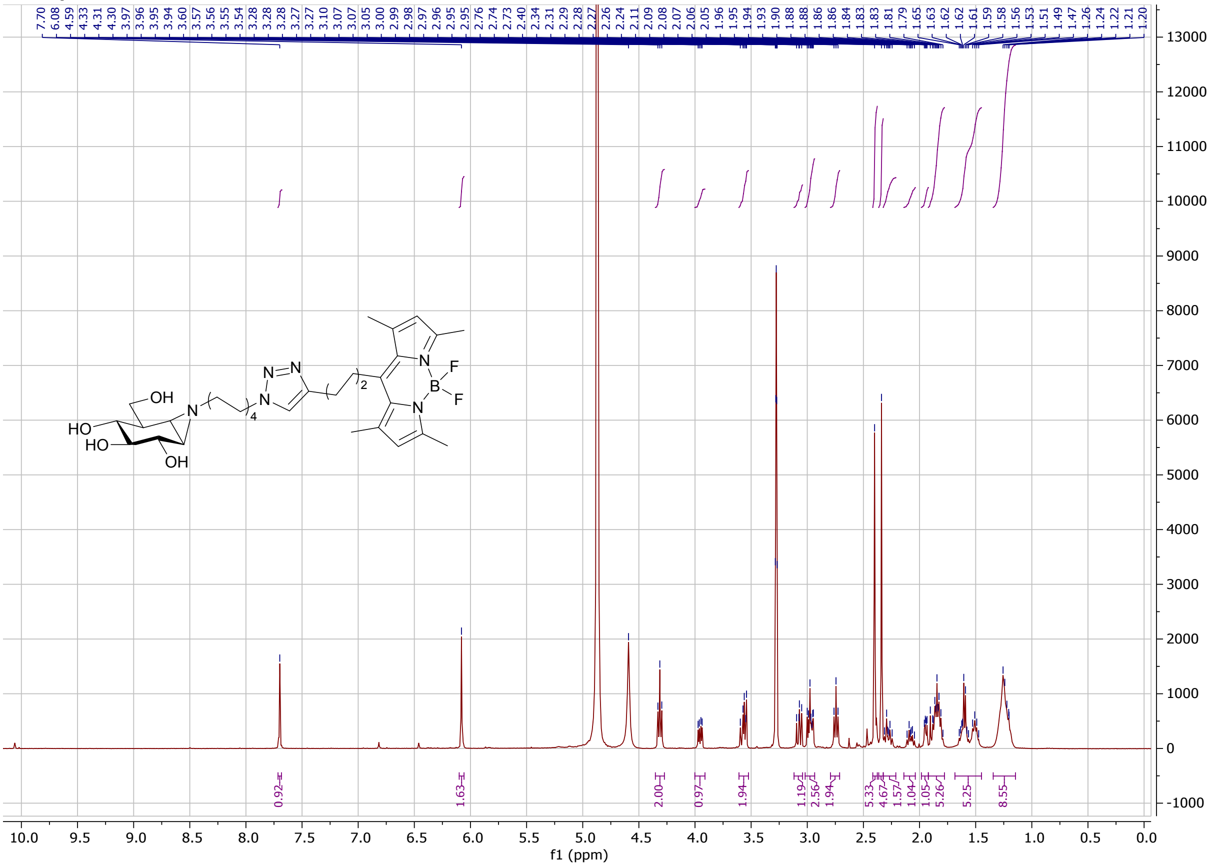

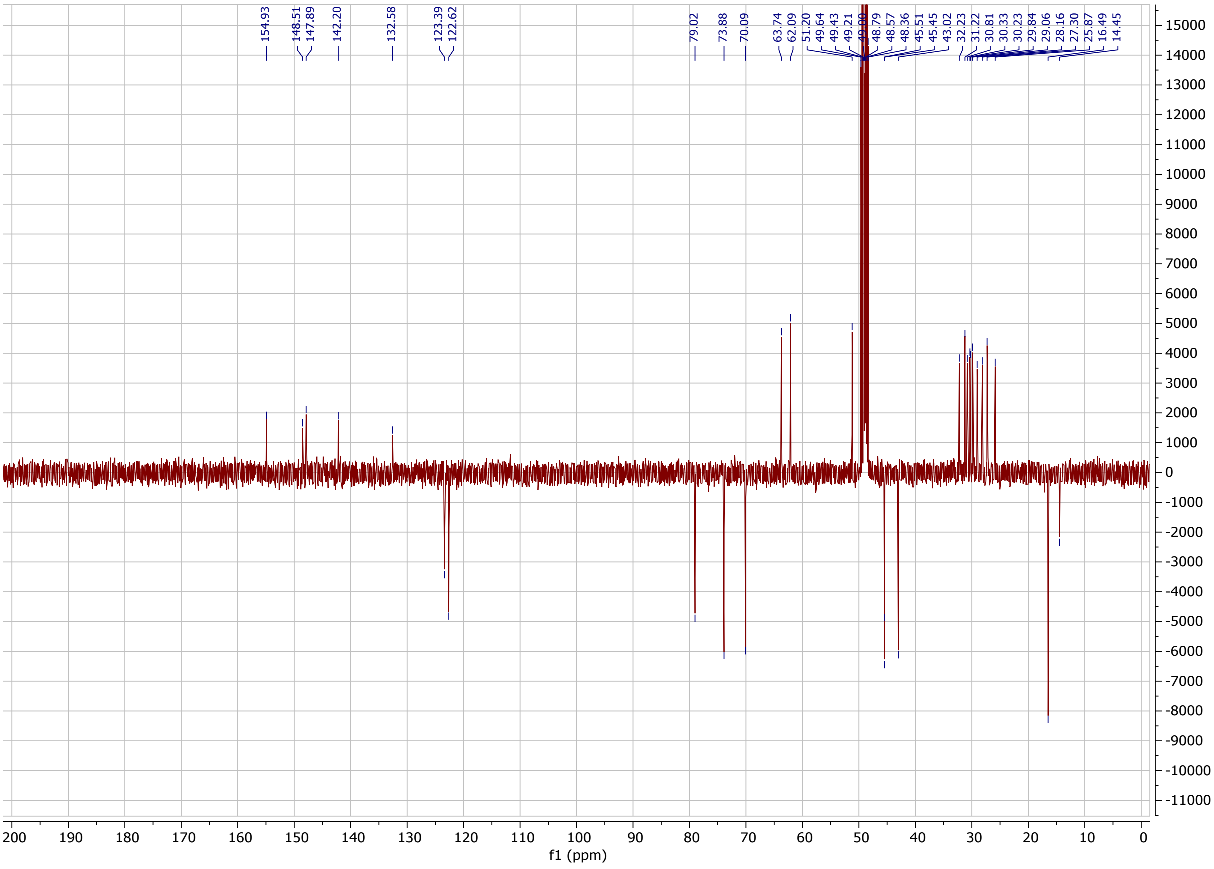

Supplement: CB-001-D0CB00045K-s001 [file CB-001-D0CB00045K-s001.pdf]
